# Time-Ordered Networks Reveal Limitations to Information Flow in Ant Colonies

**DOI:** 10.1371/journal.pone.0020298

**Published:** 2011-05-20

**Authors:** Benjamin Blonder, Anna Dornhaus

**Affiliations:** Department of Ecology and Evolutionary Biology, University of Arizona, Tucson, Arizona, United States of America; Vrije Universiteit, Netherlands

## Abstract

**Background:**

An important function of many complex networks is to inhibit or promote the transmission of disease, resources, or information between individuals. However, little is known about how the temporal dynamics of individual-level interactions affect these networks and constrain their function. Ant colonies are a model comparative system for understanding general principles linking individual-level interactions to network-level functions because interactions among individuals enable integration of multiple sources of information to collectively make decisions, and allocate tasks and resources.

**Methodology/Findings:**

Here we show how the temporal and spatial dynamics of such individual interactions provide upper bounds to rates of colony-level information flow in the ant *Temnothorax rugatulus*. We develop a general framework for analyzing dynamic networks and a mathematical model that predicts how information flow scales with individual mobility and group size.

**Conclusions/Significance:**

Using thousands of time-stamped interactions between uniquely marked ants in four colonies of a range of sizes, we demonstrate that observed maximum rates of information flow are always slower than predicted, and are constrained by regulation of individual mobility and contact rate. By accounting for the ordering and timing of interactions, we can resolve important difficulties with network sampling frequency and duration, enabling a broader understanding of interaction network functioning across systems and scales.

## Introduction

An important function of many complex networks (e.g. HIV infections, power grids, mobile phone calls) is to inhibit or promote the transmission of disease, resources, or information between individuals [1], [2]. These interactions are often critical to determining individual and group-level functions. While social network analysis has provided a powerful framework for understanding the structure of these interaction networks, it is less useful for understanding the temporal dynamics of these networks. With few exceptions [3], [4], [5], it has been hard to study empirical flows of resources or information between individuals, or to compare these dynamics across systems and scales. Our knowledge of dynamic biological interaction networks is particularly poor, perhaps due to the difficulties inherent to quantifying or manipulating large natural systems [6], [7].

Ant colonies are a model comparative system for understanding general principles linking individual-level interactions to group-level functions. Local interactions between individuals via direct antennal contact are known to be functionally important [8]. While empirical knowledge of these interactions networks is limited [9], [10], interactions among individuals enable integration of multiple sources of information to collectively make decisions [11], [12], and allocate tasks and resources [13], [14] and modulation of activity level [15] and energy usage [16]. Previous theoretical network models have linked individual behavior to colony-level oscillations in activity [17], [18] and information flow [19], and previous empirical work has demonstrated how individual mobility [20] and spatial fidelity [21] can influence colony-level functions like decision-making [22]. Nevertheless, a comprehensive and detailed picture of how individual-level interactions influence group-level information flow remains lacking.

**Figure 1:**
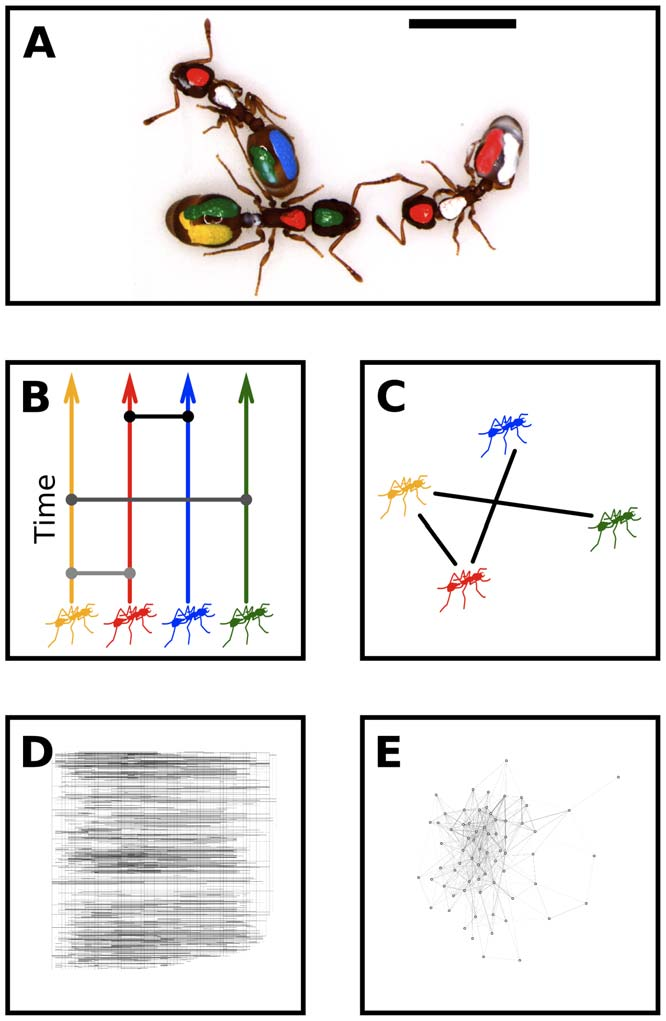
Ant interaction networks. **a**) We used marked colonies of the ant *Temnothorax rugatulus* to study the structure and dynamics of interaction networks. Interactions are a proxy for chemical or tactile communication and are defined as contact between the antenna of one ant and the body of another ant. **b**) Time-ordered networks enable inference about group-level information flow and causality from individual behavior. Individuals are linked to themselves in time and to other individuals during interactions; lines that travel horizontally or upward between nodes represent pathways for information flow. **c**) Time-aggregated networks can be recovered from time-ordered networks by accumulating data along the time axis. **d**) An empirical time-ordered network from a colony with 69 individuals, drawn as in b. **e**) The same data represented as a time-aggregated network. Time-aggregated networks inherently hide more the fundamental dynamic processes.

We developed two broadly applicable tools to understand network dynamics: first, a diffusion model to predict bounds to rates of information flow in groups of different size; and second, a ‘time ordered network’ framework for empirically tracing potential pathways of information flow through dynamic interaction networks. We used the ant *Temnothorax rugatulus* to test the hypothesis that empirical bounds to information flow would reach a theoretical bound based on the mobility of individuals. Colonies of *T. rugatulus* can be kept in artificial transparent nests that closely mimic natural conditions. In four colonies of a range of sizes (n = 6–90 individuals) we obtained complete time-stamped records of all interactions between all individuals for approximately 1800-second intervals. We uniquely identified ants by marking each with colored paints. Interactions, defined here as antenna-body contact between individuals, can convey chemical or tactile information and are a proxy for communication (Fig. 1A). To understand long-term dynamics, each colony was filmed at two time points separated by approximately three weeks (Table S1). To assess mobility of individuals, in half of these filmings we also recorded the position of every individual at every interaction. Using these data we uniformly rejected our hypothesis but were able to determine important mechanisms that limited information flow in these ant colonies.

## Results

We measured information flow by constructing time-ordered networks that preserve the temporal sequence of interaction events, enabling the enumeration of all paths for potential information flow that are causally permitted (i.e. no back-propagation in time; Fig. 1B) [23], [24]. Time-ordered networks are constructed by defining nodes for every individual at every time, and then defining directed links for individuals at sequential times and undirected links between individuals when interactions occurred (Fig. 1B,1D; for images of all time-ordered networks, see Fig. S1). To compare these networks' structure with other complex networks, we also constructed time-aggregated networks by accumulating interactions within variable time windows (Fig. 1C,1E; for images of all time-aggregated networks, see Fig. S2).

Because proximity is defined to be a prerequisite for interaction in this and many other networks [25], we tested the importance of individual mobility (rates and distances of travel) for predicting colony-level information flow and network structure. We constructed a diffusion model in which all individuals interact like kinetic gas particles [26], traveling in straight lines until they bounce off an obstacle or another individual, in which case an interaction is recorded. The model is robust to the vagaries of real motion, such as pauses and deviations from straight paths [26] This model of network dynamics can be specified by five parameters: *m*, the number of individuals; *A*, the area of the region, *D*, the mean interaction radius of an individual, *v*, the mean speed of an individual, and *t*, the time elapsed. The model makes several quantitative predictions about the structure and dynamics of interaction networks of varying size. First, information flow, the number of individuals, *n(t)*, that can be reached by a message from a focal individual after some interval of time, can be predicted using a SI epidemiology framework (details in Methods and [27]):
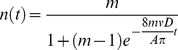
(1)Eq. 1 thus provides a general theoretical bound to rates of information flow based on easily measurable properties of individuals and groups. If there is systematic variation in individual behavior, this model may provide an over- or under-estimate of the true bound on information flow. For example, propagation is fast in networks with scale-free structure where some individuals are much more likely to interact than others [28].

We hypothesized that ant colonies would achieve this bound based on a null expectation of no systematic differences between individuals. In this scenario, individual interactions would promote rapid spread of information throughout the colony, potentially enabling efficient task allocation. In several social insect species, individuals do vary in their interaction rate and task assignments, but the consequences for information flow remain unclear [8], [10]. To test our prediction, we measured maximum information flow on our empirical time-ordered networks by allowing messages to propagate from randomly chosen individuals, measuring the number of individuals reached by a message after a time interval. Our measure provides an empirical upper bound to local rates of communication between individuals (this species of ant may also communicate non-locally with pheromones, and not every interaction is guaranteed to propagate a message). This bounding approach may also be useful for the analysis of many other networks where proximity is important to communication.

**Figure 2:**
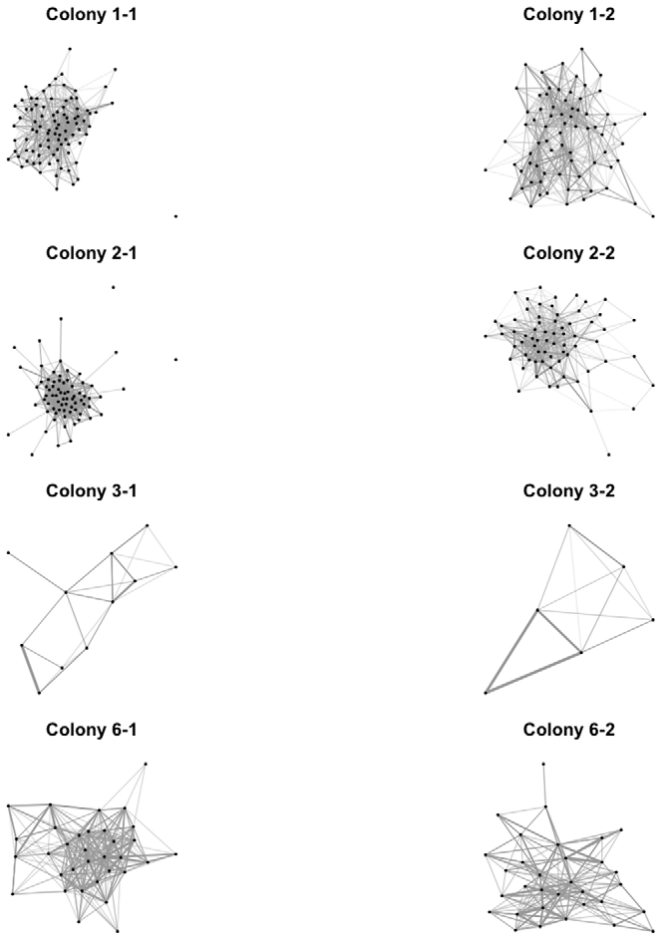
Theoretical and empirical bounds to information flow in ant colonies. We used a diffusion model to predict a theoretical bound to the number of individuals reached over time by a message from a focal individual. The model assumes that individuals interact like in a kinetic gas. We also used empirical time-ordered networks to determine an empirical upper bound to rates of information flow, assuming perfect communication. On rescaled axes determined from Eq. 1, empirical data are predicted to reach theoretical values, falling on the universal 1∶1 line. We found that colony-level information flow is significantly slower than predicted by individual mobility in the diffusion model.

We find that maximum information flow in all our ant networks is significantly slower than predicted by the model at long time scales (Fig. 2; test of slope = 1 of SMA regression on rescaled time series: p<10^−6^ for all colonies and filming sequences, all 95% CIs within 0.15–0.59). However, at short time scales, information flows faster than predicted. To also determine the minimum average time delay between information reaching an individual from any other individual through direct or indirect paths we used vector clock latencies [24]. We found a grand mean latency of 347±12 (s.e.) seconds across all ants and colonies, indicating that individual ants, on average, are no less than six minutes out of touch with other ants in the colony. In sum, individual interactions constrain the structure of ant interaction networks relative to a theoretical expectation, ensuring that information only propagates quickly locally.

**Figure 3:**
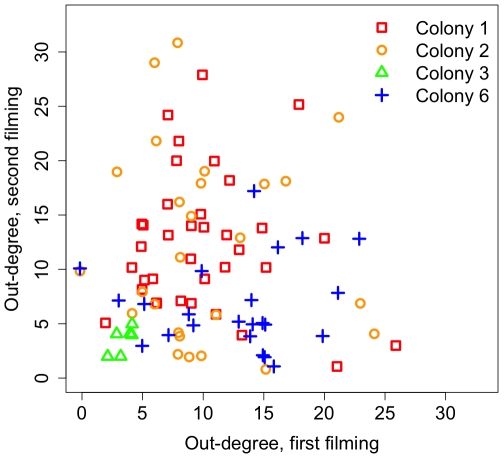
No long-term individual specialization in interaction. We found that individual ants with high degree in time-aggregated networks from over one filming session did not have high degree in subsequent filmings. This indicates that individual ants do not have long-term specialization in interaction, and that the colony-level network structure emerges from the regulated interactions of many individuals.

We tested whether this pattern was the result of the specialization of some individuals on interaction tasks. The model assumed that all individuals are unspecialized, which is equivalent to interaction rate being a Poisson-distributed random variable (Eq. S1 in Methods). Over long time scales, this assumption predicts a null relationship between the numbers of individuals a focal ant touches (out-degree) in two independent filming sequences. Over short time scales, this assumption predicts a one-to-one relationship between the numbers of individuals a focal ant touches and the number it is touched by (in-degree). Using time-aggregated networks, we indeed found no specialization: out-degree for all ants was not related to out-degree between filmings for all colonies (Fig. 3; test of slope = 0 for SMA regression, all p>0.06), and out-and in-degree for all individuals were positively correlated within filmings for all colonies (test of slope = 0 for SMA regression, all p<10^−6^, all slopes within 0.92–1.24). We also tested the hypothesis that only the queen ant might have a preferred or special role in each colony's interaction network. Across colonies in temporal and aggregated networks, we found that, relative to all other ants, the queen did not have a lower latency (mean quantile 35%±9 s.e.), higher out-degree (mean quantile 43%±12 s.e.), or higher betweenness centrality (mean quantile 54%±15 s.e.). These results support the central assumptions of the diffusion model and indicate that individual specialization cannot explain patterns of information flow.

**Figure 4:**
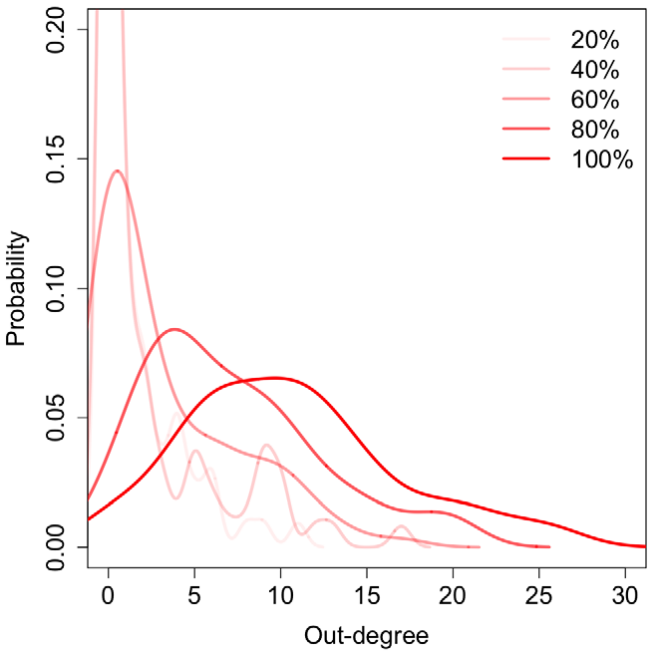
Degree distribution for a representative colony over increasing time-aggregation windows (percentages of available data). The distribution is binomial and the mean degree increases with time. Data are kernel-smoothed to ease interpretation.

**Figure 5:**
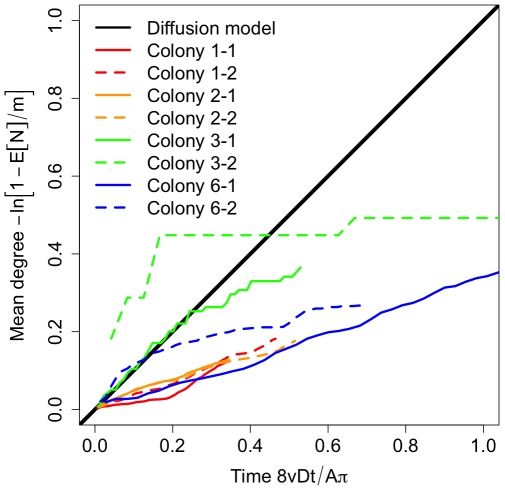
Increase in mean degree with increasing aggregation time across all colonies. Although the overall shape of each distribution is approximated by the binomial-exponential model, degree increases significantly more slowly than predicted by the diffusion model. This is consistent with the limited information flow observed in Fig. 2.

These results for specialization have implications for the topology of the interaction network. Scale-free networks, which characterize many human systems, show consistent individual specialization, with a power-law distribution of high- and low-degree individuals. In contrast, the diffusion model predicts that the out-degree distribution *N(n)* should vary in time, taking the binomial-exponential form
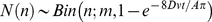
(2)Eq. 2 predicts that average out-degree increases with sampling time and will eventually converge to the maximal value *m*. We found that mean out-degree increases in our ant networks when aggregated over increasingly large time windows, though the increase is slower than predicted (Fig. 4 and Fig. 5 and Fig. S3; test of slope = 0 for SMA regression on transformed data: all p<10^−6^, all slope C.I.s within 0.17–0.70). Moreover, the form of the degree distribution for fully aggregated networks in five of eight cases was consistent with the binomial-exponential model in Eq. 2 (maximum likelihood fits of Pareto and binomial distributions: all ΔAIC>4). This slowly converging degree distribution is inconsistent with the properties of scale-free networks, and indicates that sampling window fundamentally affects the properties of time-aggregated networks.

**Figure 6:**
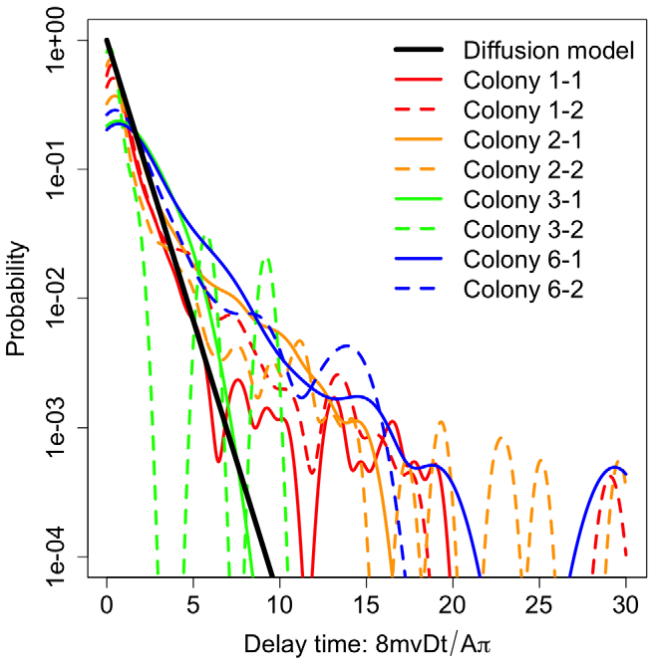
Distribution of time delays between interactions across colonies and repeated filmings. Consistent with many human networks, we found a long-tailed waiting time distribution that was often best fit by a power-law, in contrast to the exponential distribution predicted by the diffusion model (Eq. 3). These long waiting times contribute to limitations to information flow.

We also tested if information flow was limited by long time delays between interactions. Many human networks are known to have heavy-tailed (power-law) delay distributions [29]. The diffusion model predicted an exponential distribution *T(t)* of time delays between interactions:
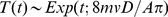
(3)We found that the diffusion model predicted the delay between interactions at short time scales but did not explain the presence of many long time delays across and within colonies (Fig. 6). To assess this long tail, we compared exponential and power law maximum-likelihood fits to these distributions. In three of eight filming sequences, power law fits were preferred over exponential fits (all ΔAIC>23), indicating that long delays may be an important feature characterizing information flow limitation.

**Figure 7:**
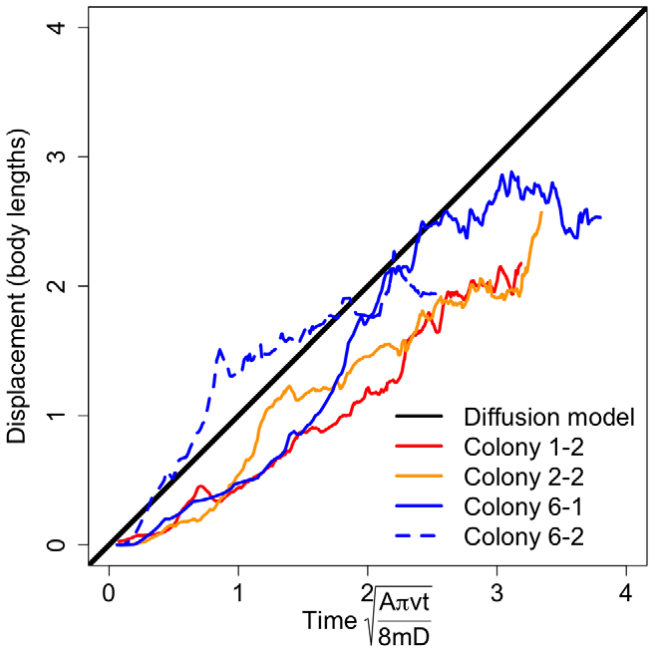
Displacement of individuals over time. As in a diffusion process, the displacement of individuals from their initial position is predicted to increase with the square root of time (Eq. S4). Across colonies, displacements increase more slowly than expected but do scale with model predictions (test of slope = 1 for SMA regression of transformed data: all p<10^−6^, all slopes in 0.67–0.91).

Another possible mechanism for limiting information flow is reduced individual movement speed or spatial fidelity to certain regions. We found that individual spatial displacement as a function of time increased more slowly than predicted by the diffusion model (Fig. 7; see also Figs. S4, S5, S6, test of slope = 1 for SMA regression of transformed data: all p<10^−6^, all slopes in 0.67–0.91). Thus, this study suggests that individual ants limit their mobility, resulting in long delays between interactions and limited information flow. Previous studies of individual spatial fidelity in related species are consistent with this finding and provide support for this mechanism [21].

## Discussion

Our results call for a deeper general understanding of the adaptive significance of different network structures. Variable rates of information flow may control efficient group function [8], [10]. Fast local flow can be adaptive for many common tasks (in ants, including brood care, resource distribution, and grooming) that can be negotiated quickly between individuals in local neighborhoods [30]. The results of these interactions may not be relevant to individuals in other locations, improving task performance in species with spatial fidelity [21]. Some ant species are known to regulate individual contact rate, sometimes by local feedback processes [8], [10], [17]. Slow global flow may limit interaction rates to maximize time available for task completion and reduce the potential for the disease spread [5]. However, network structure and information flow should change under stressful conditions when information must be globally propagated (e.g. famine relief [14] or nest destruction in ants). The temporary emergence of some highly interactive individuals, akin to ‘hubs’ in time-aggregated networks, may play an important role in these situations. Extensions of our model to consider systematic individual variation in interaction rate, and feedbacks between interactions, may be fruitful. For example, in some species of ants, colony-level oscillations in activity may be contingent upon worker interactions with brood [17]. Additionally, we are aware of very few studies [2], [14] that empirically trace the propagation of a known signal or resource under variable conditions. Comparative studies across species and systems using temporal methods are needed to explore the adaptive costs and benefits of network dynamics in different environments [31].

We have provided strong bounds to information flow in ant networks that are set by constrained mobility and regulation of interactions between unspecialized individuals. These results provide a unique perspective on the organization of ant colonies of a range of sizes and contrast strongly with the common ‘scale-free’ nature of many human systems, challenging notions of structural universality in self-organized networks. A dynamic approach using our framework and model will provide important insights into the link between individual behaviors and group function in other biological networks like food webs [32] plants and pollinators [33] and pathogens [34]. Understanding how and why the dynamics of ant networks are different from those of human communication [1], disease [2] or proximity networks [3] will shed light upon general principles that control information flow and network evolution across systems.

## Materials and Methods

### Ant marking and filming

We collected whole colonies of the ant *Temnothorax rugatulus* from the Santa Catalina Mountains, near Tucson, AZ, between 2006 and 2009. Colonies were kept in standard artificial nests consisting of a rectangular cardboard nest chamber sandwiched between two glass plates (7.5 cm×5 cm×0.1 cm). Colonies were kept in plastic boxes (10 cm×10 cm×5 cm) whose sides were coated in Fluon to prevent escape, under laboratory conditions (25°C, 25% humidity). Colonies were given water, 50% w/v sucrose solution, and cockroach parts twice weekly. The size of each artificial nest was determined as a proportion of the mass of the colony. In August 2009 we chose four colonies with a range of number of workers (6–90) for further study.

In order to distinguish individuals, we marked every worker and queen. Each ant was removed from its colony, anesthetized with CO_2_, and given a unique set of marks with acrylic paints applied with a thin wire under a dissecting microscope. Paint of red, yellow, white, green, or blue color was applied in four locations: head, thorax, left gaster, and right gaster. After the paint dried, ants were immediately revived and returned to their colony. This paint-marking technique previously has been shown to have minimal long-term effect on ant behavior. Colonies were given two days to recover after all individuals were marked.

Colonies were filmed in high definition with a digital camcorder (Canon, HV20). Conditions were standardized to mid-afternoon and ambient lighting, with the camera located above the upper glass surface of the nest. Filming occurred for approximately 30 minutes and was repeated under identical conditions approximately three weeks later. Films were converted to MPEG4 videos and stored on a computer for later analysis.

### Data analysis

We recorded all interactions between all individual ants in each video (Data S1). All ants were uniquely identified in each video. Any ant whose paint-marks had fallen off (less than 10%) was given unique temporary identifying codes. An interaction was defined as the antenna of one ant (the initiator) touching any part of another ant (the target). For each interaction we recorded the start time and identities of the initiator and target. In half of the videos we also recorded the position of the head of the target ant when the interaction was initiated. To ensure we recorded the complete set of interactions between individuals, we watched each video repeatedly, focusing on the behavior of only a single individual in each playback. For each video we also measured the area of the nest (A) and the mean body length from mouth to gaster (*l*). We used this information to calculate a mean collision radius *D = l*. We also calculated mean worker speed *v* as the mean of the quotient of the linearly interpolated position displacement between interactions and the time between interactions. In videos where position data was not recorded, we used the grand mean of worker speed in all other videos. Because videos were not all filmed at the same magnification, we scaled all measurements to be in units of mean worker body length (approximately 3 mm).

We constructed time-aggregated and time-ordered interaction networks using the ‘timeordered’ and ‘igraph’ packages in R (http://cran.r-project.org/). The ‘timeordered’ package, which we have recently made public, implements the temporal analyses presented here. Information flow on time-ordered networks was simulated by choosing 1000 focal random events per colony and tracing the number of unique individuals that could be causally reached from the focal random event over a given time interval. Vector clock latencies were calculated with codes in R following the algorithms of Kossinets *et al*. Spatial statistics were computed with the ‘spatstat’ package (http://www.spatstat.org/); model II regressions with the ‘smatr’ package (http://www.bio.mq.edu.au/ecology/SMATR/).

### Diffusion model

Because we defined interactions as requiring physical contact between ants, network structure and dynamics can also be understood by studying spatial mobility patterns. We hypothesized that random movement - kinetic gas dynamics - would be sufficient to explain many patterns of interactions in ant colonies. Such models have been previously used for biological studies of animal mobility, but only recently for the purposes of understanding information flow and collective behavior. Here we have assumed that interaction and information flow occurs only during collisions between individuals. In this model, ants behave like particles in a two-dimensional gas: all individuals have identical sizes and interaction rules, such that they walk in straight lines until they touch another ant or a wall, after which they bounce elastically off the obstacle. This model is particularly useful because it depends only on five independently measurable parameters: *m*, the number of individuals, *A*, the size of the area containing the individuals, *D*, the mean radius of an individual, *v*, the mean speed of an individual, and *t*, the time elapsed.

Following the derivation of Hutchinson & Waser, the total number of interactions *I(i)* for an individual is distributed as:
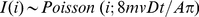
(S1)The expected number of unique interactions experienced by all individuals in the group (*I_G_*) is the product of the mean number of interactions per individual (from Eq. S1) multiplied by the total number of individuals, divided by two to correct for double-counted interactions:
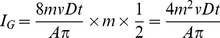
(S2)Eq. S2 demonstrates how colony-level interaction rates, which may control the tempo of decision-making in the colony, depend on physical properties of the colony like its social density and mean individual body size.

The distribution of waiting times between interactions for one individual, *T(t)*, also follows from Eq. S1 as the probability of no touches in time t, or *I(0)*:
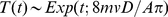
(S3)This distribution characterizes how likely it is for an individual ant to remain ignorant of the actions of the rest of the colony.

The expected resultant displacement *R* of an individual from its starting position is the product of the mean speed, the mean time between collisions (the expected value of T), and the square root of the expected number of interactions, where the square root accounts for the two-dimensional nature of the displacement:
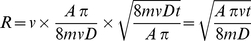
(S4)This displacement function sets the spatial scale of movement and potential territory for individual ants, suggesting a limit on how information can move between different regions of a colony. This equation is approximately valid for 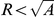
- that is, for times before displacement is limited by the boundary of the area.

The previously-discussed out-degree distribution *N(n)* (the probability of an individual having touched exactly *n* unique other individuals out of *m* total individuals after time *t*) is binomially distributed, with the success probability equal to the probability that one ant has at least one interaction with one other ant in time *t*, or *1 - T(t)* where ρ = 1/A in T(t):
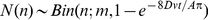
(S5)Therefore the mean degree of an individual ant is the expected value of N(n), or
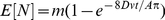
(S6)We can also predict the dynamics of information flow by using a simple SI epidemic model. Suppose that information is stored with each individual and can be propagated in the future if one ant touches another ant (that is, information can propagate only along causally permitted paths through a time-ordered interaction network). Suppose that one ant spontaneously obtains some information; then for a group of *m* total ants, the number of informed ants increases with the product of the mean per-individual contact rate *8mvD/Aπ*, the number of informed individuals *n* and the probability of encountering an uninformed individual *(m–n)/m*:
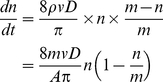
(S7)For the boundary condition *n(0) = 1* (one ant informed) this has the logistic solution
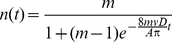
(S8)This equation demonstrates how individual mobility and colony size control how quickly information can be distributed between individuals. Imperfect information transmission due to time delays, etc. would change the form of Eq. S7.

The model makes several other quantitative predictions. First, touches should be distributed completely spatially randomly within the region, because individual ants are found with equal probability in all locations. Second, the number of touches given by an ant should be equal to the number of touches received by that ant, because the mobility model does not distinguish between the initiator and target of an interaction. Third, for long sampling intervals, the diameter of the network (constructed of links between individuals that have touched at least once) should decrease to one, because all ants will eventually have at least one interaction with each other ant. Fourth, an individual's touch rate should be predictive of its touch rate in the future only for short sampling intervals - that is, over long time scales, no ant should maintain a role as a high-functioning or low-functioning communicator, because touch rate is a Poisson-distributed random variable, and measured touch rate is necessarily averaged over some finite time interval. Lastly, the model also suggests that the queen should have no special role with respect to information flow if she moves in the same way as all other ants.

## Supporting Information

Figure S1 Time-aggregated networks for all colonies and filming sessions.
(TIFF)

Figure S2 Time-ordered networks for all colonies and filming sessions.
(TIFF)

Figure S3 Out-degree distributions over time for all colonies and filming sessions. Mean degree increases over time (larger percentages of data aggregated) as predicted by the diffusion model.
(TIFF)

Figure S4 Spatial controls on interactions and information latency. Information latency, the minimum delay time for a message to propagate from one individual to another through direct or indirect paths, increases with distance from the center of the nest (test of slope = 0 for OLS regression: p<0.01 for three of four filmings). Ants located in the center of the nest are relatively better informed, indicating spatial structure to the interaction network not predicted by the diffusion model.
(TIFF)

Figure S5 Distribution of interactions in space and time. Interactions are shown as colored dots. More recent interactions are shown in yellow; less recent ones in red. The nest entrance is located at the top center of each plot. Interactions are clustered (KS test of complete spatial randomness for x- and y- covariates, all p<10^−6^) and appear to propagate in traveling waves through the colony.
(TIFF)

Figure S6 No evidence for positive feedback for interactions. A proposed hypothesis for traveling waves of interactions in colonies is activation of individuals by interaction events (S. Boi, *Coupled oscillators and activity waves in ant colonies*. Proc. R. Soc. Lond. B (1999) 266, 371–378). To test this, we created spike-train time series for each individual ant's record of initiator interactions and target interactions. If the hypothesis were true, we would expect a large cross-correlation between these time series at a positive time lag. We found no such relationship for mean cross-correlations averaged within filmings in any colony (Wilcoxon rank sum test on cross correlations at negative and positive time lags: all p>0.17).
(TIFF)

Table S1 Summary of data collected. Asterisks (*) denote filmings in which spatial data were collected.
(DOC)

Data S1 Complete network data for all colonies.
(PDF)
