# Correction: Time-Ordered Networks Reveal Limitations to Information Flow in Ant Colonies

**DOI:** 10.1371/journal.pone.0101031

**Published:** 2014-06-20

**Authors:** The PLOS ONE Staff

Figure 2 is incorrect. The authors have provided a corrected version here.

**Figure 2:**
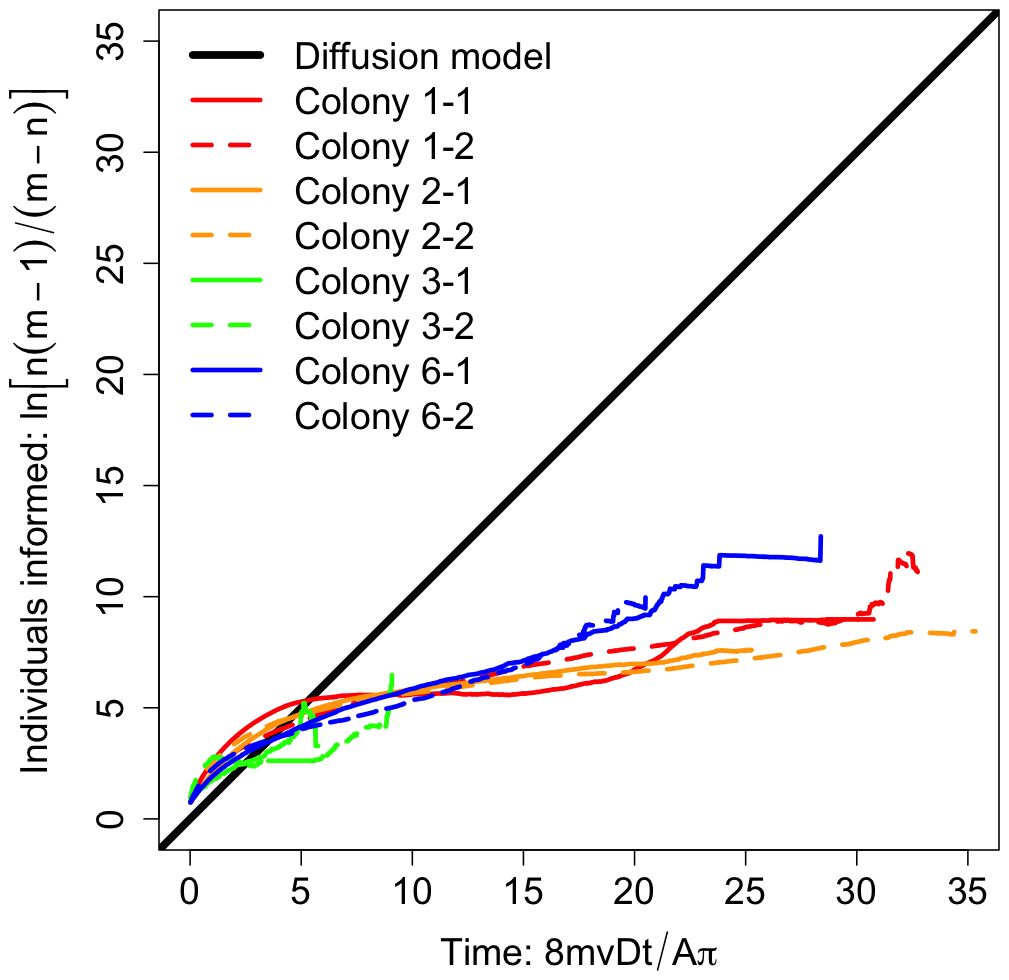
Theoretical and empirical bounds to information flow in ant colonies. We used a diffusion model to predict a theoretical bound to the number of individuals reached over time by a message from a focal individual. The model assumes that individuals interact like in a kinetic gas. We also used empirical time-ordered networks to determine an empirical upper bound to rates of information flow, assuming perfect communication. On rescaled axes determined from Eq. 1, empirical data are predicted to reach theoretical values, falling on the universal 1:1 line. We found that colony-level information flow is significantly slower than predicted by individual mobility in the diffusion model.
