# Supplementary figures and images for: Time-Ordered Networks Reveal Limitations to Information Flow in Ant Colonies
Source: PLoS One. 2011 May 20;6(5):e20298. doi: 10.1371/journal.pone.0020298 (PMC3098866; doi:10.1371/journal.pone.0020298)

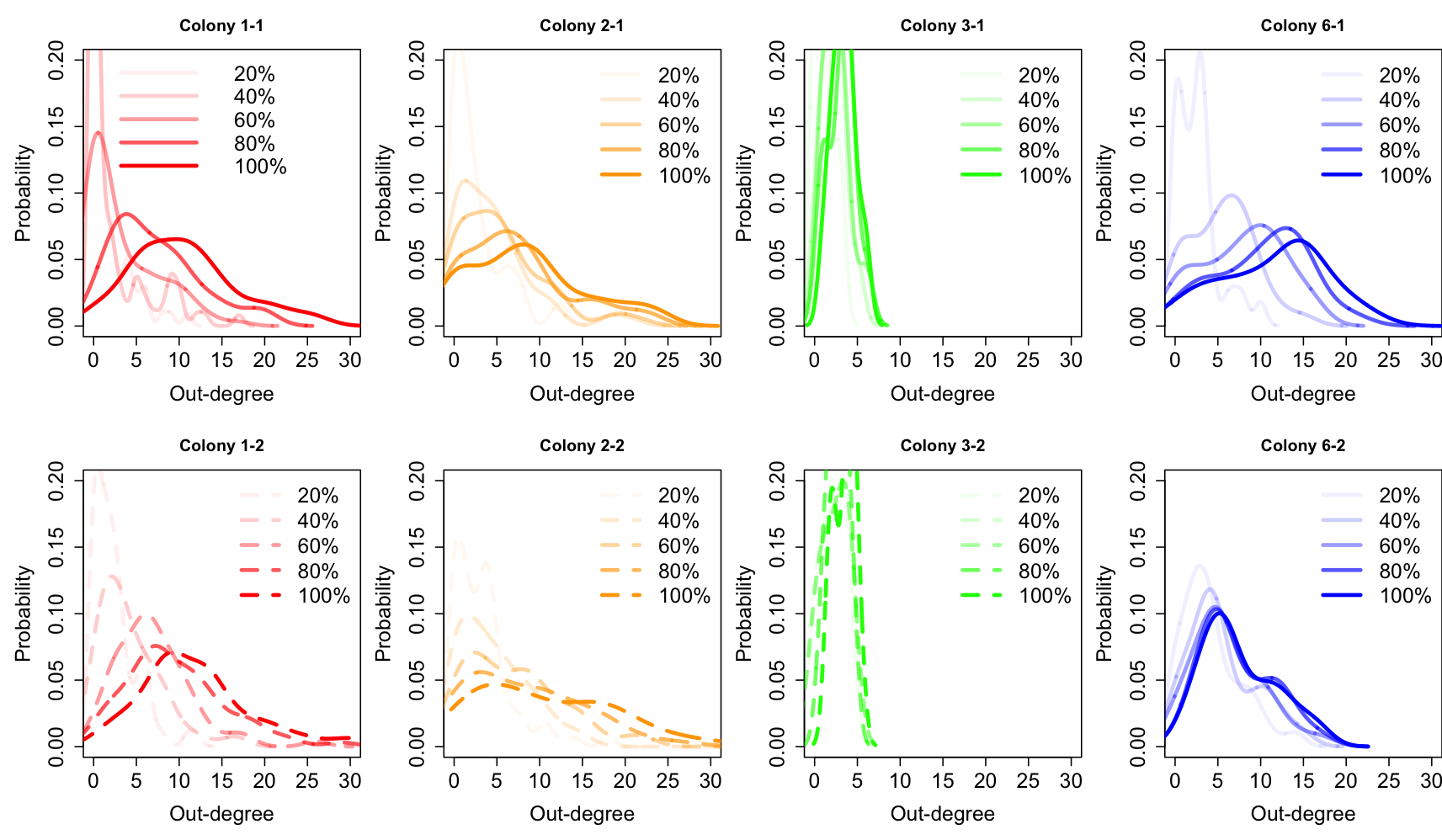

Supplement: Figure S3 — Out-degree distributions over time for all colonies and filming sessions. Mean degree increases over time (larger percentages of data aggregated) as predicted by the diffusion model. (TIFF) [file pone.0020298.s003.tiff]

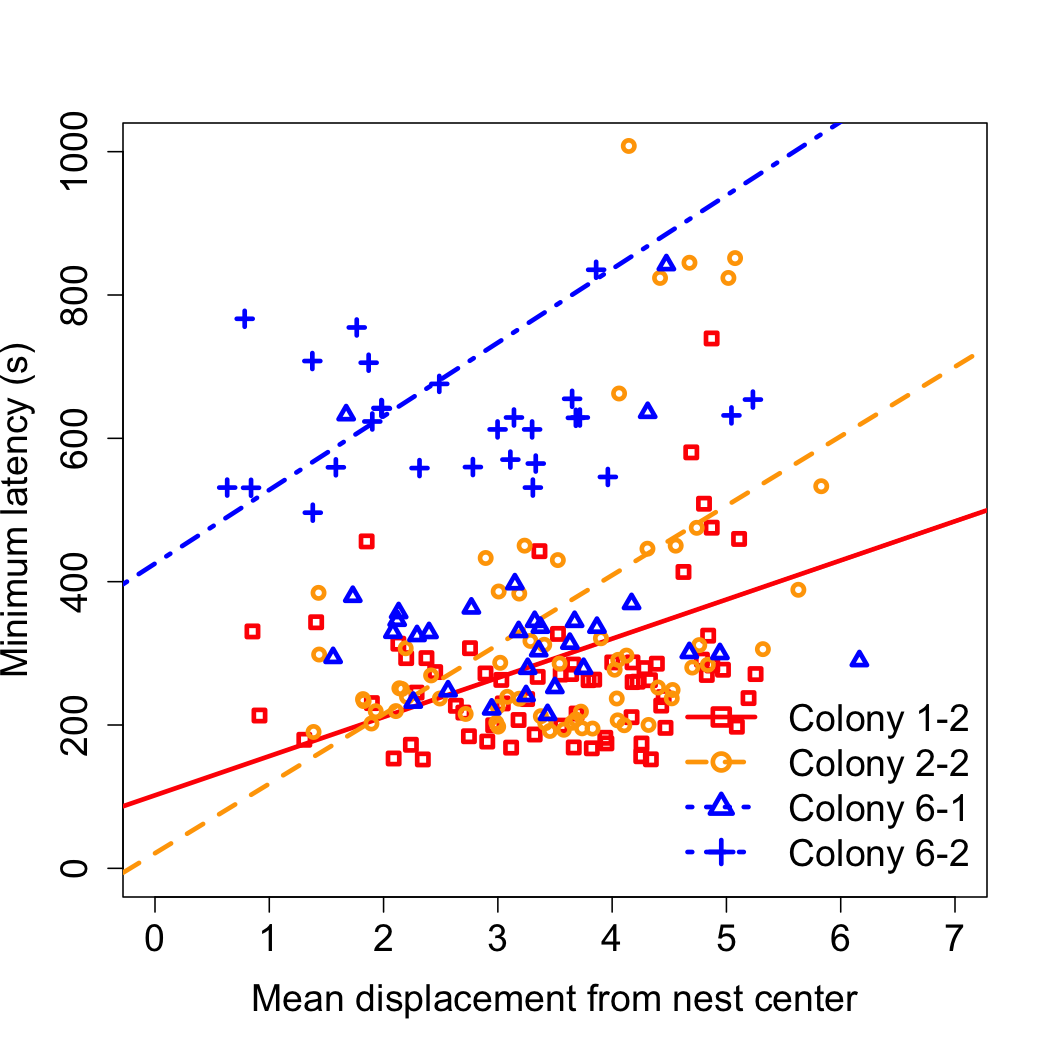

Supplement: Figure S4 — Spatial controls on interactions and information latency. Information latency, the minimum delay time for a message to propagate from one individual to another through direct or indirect paths, increases with distance from the center of the nest (test of slope = 0 for OLS regression: p<0.01 for three of four filmings). Ants located in the center of the nest are relatively better informed, indicating spatial structure to the interaction network not predicted by the diffusion model. (TIFF) [file pone.0020298.s004.tiff]

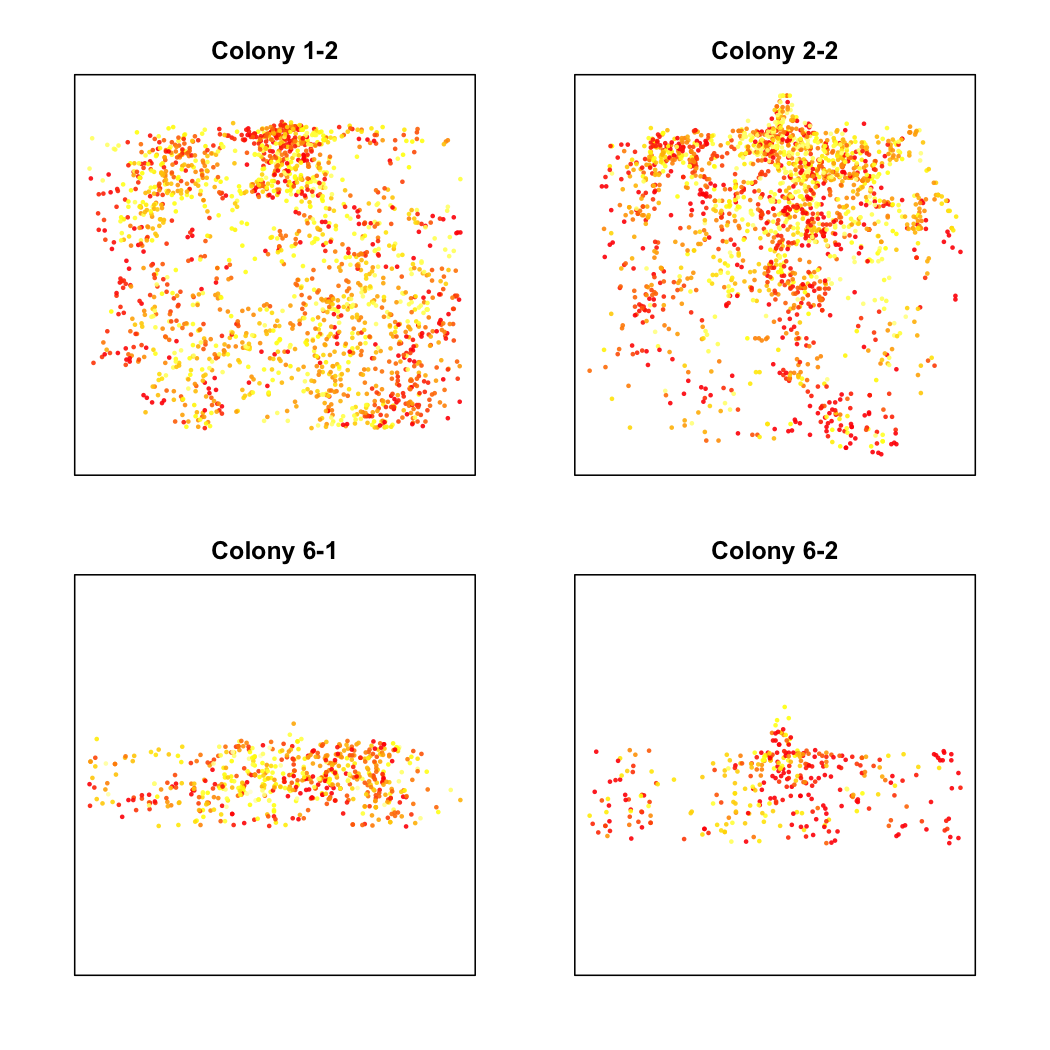

Supplement: Figure S5 — Distribution of interactions in space and time. Interactions are shown as colored dots. More recent interactions are shown in yellow; less recent ones in red. The nest entrance is located at the top center of each plot. Interactions are clustered (KS test of complete spatial randomness for x- and y- covariates, all p<10−6) and appear to propagate in traveling waves through the colony. (TIFF) [file pone.0020298.s005.tiff]

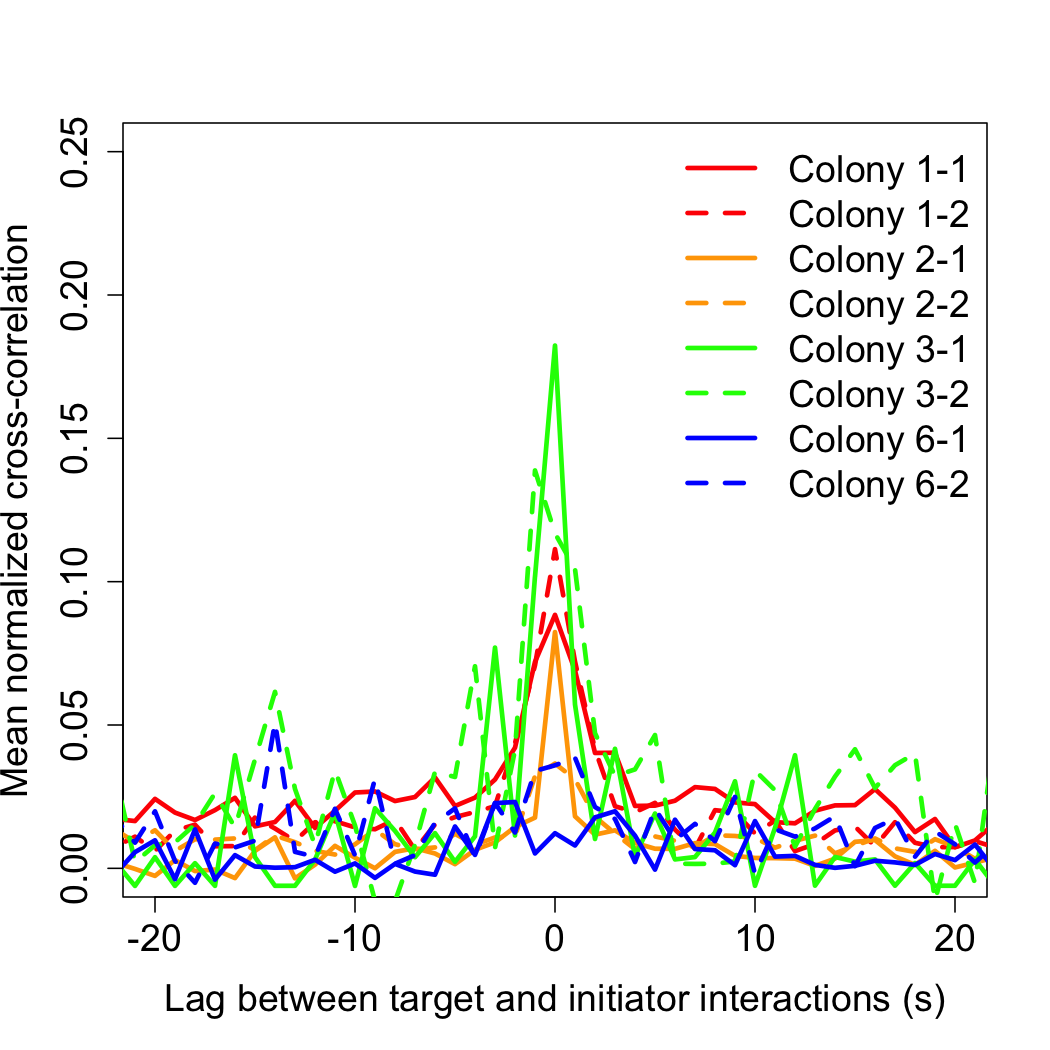

Supplement: Figure S6 — No evidence for positive feedback for interactions. A proposed hypothesis for traveling waves of interactions in colonies is activation of individuals by interaction events (S. Boi, Coupled oscillators and activity waves in ant colonies. Proc. R. Soc. Lond. B (1999) 266, 371–378). To test this, we created spike-train time series for each individual ant's record of initiator interactions and target interactions. If the hypothesis were true, we would expect a large cross-correlation between these time series at a positive time lag. We found no such relationship for mean cross-correlations averaged within filmings in any colony (Wilcoxon rank sum test on cross correlations at negative and positive time lags: all p>0.17). (TIFF) [file pone.0020298.s006.tiff]
