# Supplementary material for: Time-Ordered Networks Reveal Limitations to Information Flow in Ant Colonies
Source: PLoS One. 2011 May 20;6(5):e20298. doi: 10.1371/journal.pone.0020298 (PMC3098866; doi:10.1371/journal.pone.0020298)
Supplement: Table S1 — Summary of data collected. Asterisks (*) denote filmings in which spatial data were collected. (DOC) [file pone.0020298.s007.doc]

| Colony | Filming | Filming time (s) | Total number of interactions | Number of ants *m* | Nest area *A*  (body length^2^) | Social density *ρ* (body length s^-2^) | Mean speed *v*  (body length s^-1^) | Mean radius *D*  (body length) |
| --- | --- | --- | --- | --- | --- | --- | --- | --- |
| 1 | 1 | 1438 | 1911 | 90 | 111.3 | 0.81 | .010 | 1 |
|  | 2* | 1749 | 1820 | 72 | 100.9 | 0.71 | .011 | 1 |
| 2 | 1 | 1438 | 975 | 73 | 110.3 | 0.66 | .010 | 1 |
|  | 2* | 1796 | 1917 | 69 | 98.6 | 0.70 | .011 | 1 |
| 3 | 1 | 1139 | 78 | 11 | 57.1 | 0.19 | .010 | 1 |
|  | 2 | 1425 | 104 | 6 | 18.1 | 0.33 | .010 | 1 |
| 6 | 1* | 1918 | 652 | 33 | 47.6 | 0.69 | .013 | 1 |
|  | 2* | 1755 | 367 | 32 | 43.7 | 0.73 | .007 | 1 |
