# Supplementary material for: Time-Ordered Networks Reveal Limitations to Information Flow in Ant Colonies
Source: PLoS One. 2011 May 20;6(5):e20298. doi: 10.1371/journal.pone.0020298 (PMC3098866; doi:10.1371/journal.pone.0020298)
Supplement: Data S1 — Complete network data for all colonies. (PDF) [file pone.0020298.s008.pdf]

Complete network data
Data files are named in files as x-y-z.csv, where
x: Colony identifier (1,2,3,6)
y: Filming session (1,2)
z: Data type ('data', time-ordered interactions; 'meta', information on colony-level statistics)

Structure of 'meta' files:
Antlist: a list of all ants in the filming, including individuals that have no interactions. Only queen ants have a 'Q' in their name.
InitialPosX/Y: Position in pixels of each ant's head centroid
ColonyArea: Area of nest in pixels^2
AntLength: mean body length of ants in pixels
MinX/Y, MaxX/Y: coordinates in pixels of interaction bounding box. Areas computed from these numbers will be larger than the nest area as it includes all regions in which interactions could occur, e.g. in the nest entrance or immediately outside it.

Structure of 'data' files:
Actor: Name of ant initiating an interaction by extending antenna to contact another ant's body or antenna
Target: Name of the ant being touched by another ant's antenna
ActorPosX/Y: When recorded, the position in pixels of the actor ant's head centroid
Time: Time in seconds since beginning of filming session at which the interaction was initiated

Movies S1-8
Video files for each filming session are named as Colony x mm-dd-yy.m4v, where
x: Colony identifier (1, 2, 3, 6)
mm: Filming month
dd: Filming day
yy: Filming year
For all videos, the video with the oldest date is the '-1' filming session; the newer date is the '-2' filming session.

Actor	Target	Time	ActorPosX	ActorPosY
GBGW	WBYG	1093	0	0
GBGW	WBYG	1104	0	0
GBGW	WBYG	1121	0	0
GBGW	WBYG	1161	0	0
GBGW	_____pale	1214	0	0
GBGW	_____pale	1232	0	0
GBGW	_____pale	1247	0	0
GBGW	WBYG	1294	0	0
GBGW	GR_Y2	1329	0	0
GBGW	YGWW	1415	0	0
WBYG	YGWY	153	0	0
WBYG	YGWY	175	0	0
WBYG	YGWY	950	0	0
WBYG	W___	1008	0	0
WBYG	W___	1028	0	0
WBYG	GBGW	1090	0	0
WBYG	GBGW	1103	0	0
WBYG	YGWY	1127	0	0
WBYG	YYWR	1141	0	0
WBYG	GBGW	1160	0	0
WBYG	GBGW	1170	0	0
WBYG	_____pale	1238	0	0
WBYG	_____pale	1267	0	0
WBYG	_____pale	1310	0	0
WBYG	YYWR	1320	0	0
WBYG	_____pale	1357	0	0
WBYG	YGWW	1408	0	0
_____bot	_____corner	875	0	0
_____bot	_WYG	1051	0	0
_____bot	GGRY	1111	0	0
_____bot	GGRY	1164	0	0
_____bot	_____pale	1290	0	0
_____bot	_____pale	1300	0	0
_____bot	_____pale	1359	0	0
_____bot	YYWR	1438	0	0
YYWR	_____bot	278	0	0
YYWR	_____bot	1048	0	0
YYWR	_____bot	1065	0	0
YYWR	W___	1080	0	0
YYWR	WBYG	1084	0	0
YYWR	GGWY	1118	0	0
YYWR	WBYG	1140	0	0
YYWR	GGRY	1238	0	0
YYWR	_____pale	1245	0	0
YYWR	GGRY	1265	0	0
YYWR	W___	1288	0	0
YYWR	_____bot	1425	0	0
YYWR	GRBR	1435	0	0

YGWY	WBYG	182	0	0
YGWY	WBYG	1066	0	0
YGWY	W___	1112	0	0
YGWY	GGRY	1234	0	0
YGWY	GGRY	1264	0	0
YGWY	YYWR	1318	0	0
YGWY	GR_Y2	1435	0	0
YGWY	GR_Y2	1438	0	0
W___	WR__	994	0	0
W___	GGRY	1005	0	0
W___	YGWY	1048	0	0
W___	WR__	1115	0	0
W___	GGRY	1146	0	0
W___	_____brood	1235	0	0
W___	GRBR	1299	0	0
W___	GRBR	1310	0	0
WR__	GG_W	980	0	0
WR__	GG_W	1037	0	0
WR__	GGWY	1099	0	0
WR__	_____pale	1151	0	0
WR__	_WYW	1160	0	0
WR__	_____pale	1163	0	0
WR__	_____pale	1170	0	0
WR__	GR_Y2	1209	0	0
WR__	GGWW	1273	0	0
WR__	Q	1288	0	0
WR__	GG_W	1298	0	0
WR__	Q	1305	0	0
WR__	GG_W	1312	0	0
WR__	YYGG	1352	0	0
WR__	_____brood	1375	0	0
GGRY	W___	994	0	0
GGRY	GG_W	1000	0	0
GGRY	GG_W	1002	0	0
GGRY	W___	1004	0	0
GGRY	GGWY	1018	0	0
GGRY	YYGG	1047	0	0
GGRY	_WYG	1099	0	0
GGRY	GGWY	1105	0	0
GGRY	W___	1120	0	0
GGRY	YGWY	1168	0	0
GGRY	_____brood	1212	0	0
GGRY	WBYG	1216	0	0
GGRY	YGWY	1234	0	0
GGRY	YYWR	1260	0	0
GGRY	YGWY	1263	0	0
GGRY	_____bot	1277	0	0
GGRY	YGWW	1288	0	0
GGRY	_____bot	1295	0	0

GGRY	_____bot	1303	0	0
GGRY	_____pale	1306	0	0
GGRY	_____pale	1332	0	0
GBGR	GGWY	1032	0	0
GBGR	WR__	1055	0	0
GBGR	WR__	1067	0	0
GBGR	GGWY	1088	0	0
GBGR	W___	1120	0	0
GBGR	_____brood	1158	0	0
GBGR	_____pale	1188	0	0
GBGR	YYRB	1238	0	0
GBGR	YWW_	1424	0	0
GBGR	WBGG	1426	0	0
YYRB	WRRY	527	0	0
YYRB	WRRY	568	0	0
YYRB	GWRG	688	0	0
YYRB	_____corner	888	0	0
YYRB	GGWY	1055	0	0
YYRB	GGW_	1114	0	0
YYRB	GGW_	1123	0	0
YYRB	_____pale	1184	0	0
YYRB	GBGR	1255	0	0
YYRB	GR_Y2	1374	0	0
YYRB	GR_Y2	1404	0	0
YYRB	GR_Y2	1414	0	0
YYRB	GR_Y2	1429	0	0
YYRB	YGWW	1434	0	0
YYY_	YYGG	915	0	0
YYY_	GY__	996	0	0
YYY_	GY__	1128	0	0
WRRY	YYGG	527	0	0
WRRY	YYRB	572	0	0
WRRY	GWRG	593	0	0
WRRY	GWRG	606	0	0
WRRY	YWW_	704	0	0
WRRY	YYGG	732	0	0
WRRY	YYGG	819	0	0
WRRY	WR__	833	0	0
WRRY	YYGG	838	0	0
WRRY	WR__	844	0	0
WRRY	GRWG	891	0	0
WRRY	_____pale	953	0	0
WRRY	_____pale	968	0	0
WRRY	_R__	975	0	0
WRRY	GRGY	980	0	0
WRRY	YWW_	992	0	0
WRRY	_R__	1025	0	0
WRRY	Q	1075	0	0
WRRY	YWW_	1080	0	0

WRRY	YWW_	1100	0	0
WRRY	Q	1160	0	0
WRRY	_R__	1200	0	0
WRRY	GWRG	1220	0	0
WRRY	YWW_	1235	0	0
WRRY	GR_Y2	1254	0	0
WRRY	GG_W	1417	0	0
YYGG	GBGR	5	0	0
YYGG	GGWY	11	0	0
YYGG	GGWY	35	0	0
YYGG	WRWR	75	0	0
YYGG	WRWR	188	0	0
YYGG	WRWR	207	0	0
YYGG	GWRG	224	0	0
YYGG	WRWR	267	0	0
YYGG	WRRY	408	0	0
YYGG	WRRY	529	0	0
YYGG	YWW_	607	0	0
YYGG	YWW_	668	0	0
YYGG	WRRY	732	0	0
YYGG	YGWW	740	0	0
YYGG	WRRY	744	0	0
YYGG	YYGGmid	754	0	0
YYGG	YWW_	760	0	0
YYGG	YWW_	781	0	0
YYGG	GWRG	808	0	0
YYGG	____brood	812	0	0
YYGG	GRWG	822	0	0
YYGG	____brood	824	0	0
YYGG	_R__	828	0	0
YYGG	GGRR	832	0	0
YYGG	YWW_	892	0	0
YYGG	_R__	893	0	0
YYGG	YYY_	914	0	0
YYGG	GWRG	924	0	0
YYGG	GWRG	965	0	0
YYGG	GGWY	975	0	0
YYGG	YY__	1007	0	0
YYGG	GGRY	1049	0	0
YYGG	_WYG	1069	0	0
YYGG	GYGG	1077	0	0
YYGG	YYGGmid	1112	0	0
YYGG	__BB	1120	0	0
YYGG	__BB	1125	0	0
YYGG	GRYY	1137	0	0
YYGG	YYGGmid	1144	0	0
YYGG	_Y__	1157	0	0
YYGG	GRYY	1175	0	0
YYGG	GYGG	1194	0	0

YYGG	GYGG	1207	0	0
YYGG	_WYG	1208	0	0
YYGG	YGWW	1275	0	0
YYGG	GGRY	1387	0	0
WRWR	YYGG	77	0	0
WRWR	YWW_	159	0	0
WRWR	YWW_	172	0	0
WRWR	YYGG	188	0	0
WRWR	YYGG	207	0	0
WRWR	YWW_	225	0	0
WRWR	GRWG	246	0	0
WRWR	GRWG	261	0	0
WRWR	YWW_	267	0	0
WRWR	GRWG	272	0	0
WRWR	YWW_	305	0	0
WRWR	YWW_	407	0	0
WRWR	GGRR	408	0	0
WRWR	YWW_	678	0	0
WRWR	WRBB	696	0	0
WRWR	YYGG	744	0	0
WRWR	YYGGmid	811	0	0
WRWR	WRRY	833	0	0
WRWR	GGWY	874	0	0
WRWR	_WWY	895	0	0
WRWR	_WWY	910	0	0
WRWR	_WWY	915	0	0
WRWR	GRGY	994	0	0
WRWR	____bm	1217	0	0
WRWR	____bm	1219	0	0
WRWR	GGWW	1274	0	0
WRWR	GGRR	1300	0	0
WRWR	WG_R	1312	0	0
WRWR	GGRR	1317	0	0
WRWR	WGGB	1411	0	0
GY__	WRRY	140	0	0
GY__	WRRY	242	0	0
GY__	WRRY	315	0	0
GY__	WRRY	566	0	0
GY__	WRRY	595	0	0
GY__	GWRG	601	0	0
GY__	GWRG	750	0	0
GY__	GWRG	817	0	0
GY__	GWRG	960	0	0
GY__	YYY_	998	0	0
GY__	GWRG	1074	0	0
GY__	YYY_	1135	0	0
GY__	G___small	1153	0	0
GY__	G___small	1282	0	0
GY__	_WWY	1364	0	0

G___big	G___small	80	0	0
G___big	G___small	140	0	0
G___big	G___small	158	0	0
G___big	_R__	198	0	0
G___big	YY_W	1027	0	0
G___small	YW__	7	0	0
G___small	YW__	19	0	0
G___small	G___big	125	0	0
G___small	G___big	141	0	0
G___small	_R__	370	0	0
G___small	G___big	398	0	0
G___small	YY_W	1024	0	0
G___small	GY__	1154	0	0
G___small	GY__	1245	0	0
G___small	G___big	1275	0	0
G___small	GY__	1279	0	0
G___small	_WWY	1303	0	0
G___small	_WWY	1325	0	0
G___small	YYY_	1349	0	0
G___small	_WWY	1344	0	0
_R__	G___big	199	0	0
_R__	G___small	369	0	0
_R__	GWRG	383	0	0
_R__	GY__	389	0	0
_R__	GRWG	413	0	0
_R__	GRWG	415	0	0
_R__	GRWG	750	0	0
_R__	GRWG	781	0	0
_R__	GRWG	807	0	0
_R__	YWW_	831	0	0
_R__	GGRR	837	0	0
_R__	WRRY	872	0	0
_R__	GRWG	881	0	0
_R__	_WWY	890	0	0
_R__	GRWG	903	0	0
_R__	WRRY	908	0	0
_R__	YGWW	920	0	0
_R__	YGWW	938	0	0
_R__	YW__	966	0	0
_R__	____pale	976	0	0
_R__	____corner	979	0	0
_R__	WGGB	995	0	0
_R__	____brood	998	0	0
_R__	_WWY	1031	0	0
_R__	WRRY	1067	0	0
_R__	_WWY	1097	0	0
_R__	GR_Y2	1100	0	0
_R__	_WWY	1115	0	0
_R__	_WYW	1147	0	0

_R__	GR_Y2	1176	0	0
_R__	WRRY	1179	0	0
_R__	Q	1191	0	0
_R__	_WWY	1212	0	0
_R__	__W_	1219	0	0
_R__	G_GW	1238	0	0
_R__	YWW_	1260	0	0
_R__	WWBG	1286	0	0
_R__	GWRG	1345	0	0
_R__	____corner	1435	0	0
_R__	__W_	1438	0	0
GWRG	GRWG	60	0	0
GWRG	YYGG	225	0	0
GWRG	YWW_	570	0	0
GWRG	GY__	600	0	0
GWRG	WRRY	607	0	0
GWRG	YYRB	652	0	0
GWRG	WRRY	658	0	0
GWRG	YYRB	688	0	0
GWRG	YYRB	714	0	0
GWRG	GRWG	876	0	0
GWRG	YYGG	925	0	0
GWRG	GGWY	1048	0	0
GWRG	GY__	1074	0	0
GWRG	____pale	1178	0	0
GWRG	_R__	1181	0	0
GWRG	WRRY	1298	0	0
GWRG	GG_W	1337	0	0
GWRG	YYGGmid	1367	0	0
GWRG	YYGGmid	1374	0	0
YYYY	GRWG	58	0	0
YYYY	GWRG	261	0	0
YYYY	GGRR	414	0	0
YYYY	_R__	597	0	0
YYYY	GBGR	1331	0	0
____brood	GRWG	10	0	0
____brood	WRWR	93	0	0
____brood	GRWG	350	0	0
____brood	GRWG	375	0	0
____brood	GRWG	796	0	0
____brood	YYGG	813	0	0
____brood	YYGG	825	0	0
____brood	YYGG	860	0	0
____brood	G_GW	987	0	0
____brood	_WWY	1048	0	0
____brood	YYYY	1085	0	0
____brood	GWRG	1118	0	0
____brood	Q	1151	0	0
____brood	____pale	1169	0	0

_____brood	_R__	1190	0	0
_____brood	GG_W	1210	0	0
_____brood	W___	1234	0	0
_____brood	GR_Y2	1301	0	0
_____brood	GWRG	1369	0	0
_____brood	_R__	1376	0	0
_____brood	WBGG	1381	0	0
_____brood	YGWW	1438	0	0
YWW_	GRWG	247	0	0
YWW_	WRWR	267	0	0
YWW_	GGRR	277	0	0
YWW_	WRWR	283	0	0
YWW_	GGRR	388	0	0
YWW_	_R__	560	0	0
YWW_	GWRG	568	0	0
YWW_	YYGG	590	0	0
YWW_	YYGG	608	0	0
YWW_	GGRR	629	0	0
YWW_	__W_	640	0	0
YWW_	YYGGmid	648	0	0
YWW_	WRWR	660	0	0
YWW_	WRRY	703	0	0
YWW_	YYGG	709	0	0
YWW_	GGRR	778	0	0
YWW_	_R__	834	0	0
YWW_	WRRY	1081	0	0
YWW_	WRRY	1088	0	0
YWW_	WRRY	1109	0	0
YWW_	_WWY	1157	0	0
YWW_	GBGR	1321	0	0
YWW_	_R__	1431	0	0
WWBG	_RYG	1031	0	0
WWBG	GYYY	1050	0	0
WWBG	G_GW	1195	0	0
WWBG	G_GW	1199	0	0
WWBG	GYYY	1236	0	0
WWBG	_RYG	1240	0	0
WWBG	G_GW	1253	0	0
WWBG	_R__	1288	0	0
WWBG	G_GW	1297	0	0
WWBG	Y__W	1316	0	0
WWBG	W___	1350	0	0
_____topleft	WG_R	12	0	0
_____topleft	GYYY	40	0	0
_____topleft	WRR_	92	0	0
_____topleft	WG_R	140	0	0
_____topleft	__W_	154	0	0
_____topleft	_W__	158	0	0
_____topleft	GR_Y	180	0	0

_____topleft	WG_R	261	0	0
_____topleft	G_GW	291	0	0
_____topleft	YWGW	335	0	0
_____topleft	YGWW	378	0	0
_____topleft	_W__	416	0	0
_____topleft	_W__	1020	0	0
_____topleft	WG_R	1034	0	0
_____topleft	Y_WY	1047	0	0
_____topleft	_WGG	1119	0	0
_____topleft	Y_WY	1211	0	0
_____topleft	_WGG	1262	0	0
_____topleft	WG_R	1281	0	0
_____topleft	WG_R	1300	0	0
_____topleft	WG_R	1355	0	0
_____topleft	WG_R	1378	0	0
WG_R	YWGW	3	0	0
WG_R	_____topleft	15	0	0
WG_R	YWGW	19	0	0
WG_R	GRWG	32	0	0
WG_R	GGRR	34	0	0
WG_R	YYGGmid	38	0	0
WG_R	G_GW	44	0	0
WG_R	YGWW	69	0	0
WG_R	YGWW	103	0	0
WG_R	Y_WY	212	0	0
WG_R	YYGGmid	219	0	0
WG_R	Y_WY	233	0	0
WG_R	GR_Y	267	0	0
WG_R	GR_Y	330	0	0
WG_R	YYGGmid	360	0	0
WG_R	Y_WY	333	0	0
WG_R	_W__	372	0	0
WG_R	__W_	429	0	0
WG_R	YGWW	437	0	0
WG_R	Y_WY	508	0	0
WG_R	WRR_	566	0	0
WG_R	GR_Y2	607	0	0
WG_R	Y_WY	618	0	0
WG_R	_W__	676	0	0
WG_R	GGRR	693	0	0
WG_R	Y_WY	824	0	0
WG_R	GR_Y2	863	0	0
WG_R	WRBB	870	0	0
WG_R	Y_WY	890	0	0
WG_R	YYGGmid	917	0	0
WG_R	YYGG	990	0	0
WG_R	Y_WY	1004	0	0
WG_R	Y_WY	1088	0	0
WG_R	Y_WY	1120	0	0

WG_R	____topleft	1163	0	0
WG_R	____topleft	1211	0	0
WG_R	Y_WY	1227	0	0
WG_R	WGGB	1250	0	0
WG_R	Y_WY	1265	0	0
WG_R	____topleft	1290	0	0
WG_R	____topleft	1317	0	0
WG_R	____bm	1333	0	0
WG_R	____topleft	1375	0	0
WG_R	Y_WY	1385	0	0
WG_R	GR_Y2	1413	0	0
YWGW	GGRR	5	0	0
YWGW	GGRR	23	0	0
YWGW	G_GW	27	0	0
YWGW	G_GW	41	0	0
YWGW	WG_R	45	0	0
YWGW	YYGGmid	54	0	0
YWGW	Y_WY	59	0	0
YWGW	Y_WY	73	0	0
YWGW	G_GW	134	0	0
YWGW	Y_WY	170	0	0
YWGW	GGRR	190	0	0
YWGW	GGRR	210	0	0
YWGW	Y_WY	223	0	0
YWGW	Y_WY	300	0	0
YWGW	GR_Y2	340	0	0
YWGW	W___	440	0	0
YWGW	_W__	451	0	0
YWGW	G_GW	458	0	0
YWGW	_W__	480	0	0
YWGW	_W__	600	0	0
YWGW	____corner	1080	0	0
G_R_	GRWG	11	0	0
G_R_	GRWG	28	0	0
G_R_	____brood	34	0	0
G_R_	____topleft	41	0	0
G_R_	____brood	58	0	0
G_R_	GWRG	82	0	0
G_R_	GYYY	258	0	0
G_R_	GGRR	333	0	0
G_R_	GRWG	352	0	0
G_R_	GGRR	356	0	0
G_R_	YWW_	382	0	0
G_R_	_R__	405	0	0
G_R_	GWRG	411	0	0
G_R_	YYYY	420	0	0
G_R_	YWW_	431	0	0
G_R_	YYGG	459	0	0
G_R_	GYYY	464	0	0

G_R_	YWW_	546	0	0
G_R_	YYGG	577	0	0
G_R_	YWW_	622	0	0
G_R_	YWW_	637	0	0
G_R_	____brood	650	0	0
G_R_	YWW_	773	0	0
G_R_	YYGGmid	792	0	0
G_R_	____brood	809	0	0
G_R_	YYGG	833	0	0
G_R_	_R__	841	0	0
G_R_	YGWW	873	0	0
G_R_	YYGGmid	885	0	0
G_R_	WGGB	904	0	0
G_R_	WGGB	919	0	0
G_R_	Q	970	0	0
G_R_	WGWB	1000	0	0
G_R_	GR_Y2	1081	0	0
G_R_	____pale	1156	0	0
G_R_	_WYW	1160	0	0
G_R_	YYGW	1298	0	0
G_R_	WGGB	1314	0	0
G_R_	WRWR	1320	0	0
G_R_	WG_R	1331	0	0
G_R_	_R__	1353	0	0
G_R_	____bm	1360	0	0
GRWG	WRR_	5	0	0
GRWG	G_R_	14	0	0
GRWG	WRR_	19	0	0
GRWG	WG_R	28	0	0
GRWG	G_R_	35	0	0
GRWG	GWRG	59	0	0
GRWG	WRBB	79	0	0
GRWG	Y_WY	93	0	0
GRWG	YYGGmid	94	0	0
GRWG	Y_WY	105	0	0
GRWG	YWW_	144	0	0
GRWG	__W_	153	0	0
GRWG	GGWY	190	0	0
GRWG	WRWR	210	0	0
GRWG	YWW_	247	0	0
GRWG	____brood	350	0	0
GRWG	WRR_	369	0	0
GRWG	_R__	414	0	0
GRWG	_R__	781	0	0
GRWG	____brood	793	0	0
GRWG	_R__	804	0	0
GRWG	_R__	823	0	0
GRWG	____brood	829	0	0
GRWG	GWRG	849	0	0

GRWG	WRRY	861	0	0
GRWG	WRRY	875	0	0
GRWG	_R__	880	0	0
GRWG	G_R_	890	0	0
GRWG	YGWW	897	0	0
GRWG	____pale	913	0	0
GRWG	YYGGmid	920	0	0
GRWG	G_R_	935	0	0
GRWG	WGGB	948	0	0
GRWG	WRWR	960	0	0
GRWG	Q	962	0	0
GRWG	YYRG	967	0	0
GRWG	____brood	974	0	0
GRWG	_R__	980	0	0
GRWG	WRRY	983	0	0
GRWG	____brood	985	0	0
GRWG	____brood	989	0	0
GRWG	WG_R	992	0	0
GRWG	____corner	1000	0	0
GRWG	____corner	1240	0	0
GRWG	____corner	1280	0	0
GRWG	YYGGmid	1304	0	0
GRWG	____corner	1306	0	0
WRR_	GGRR	1	0	0
WRR_	GRWG	14	0	0
WRR_	G_R_	25	0	0
WRR_	GRWG	39	0	0
WRR_	GGRR	47	0	0
WRR_	WG_R	95	0	0
WRR_	GGRR	97	0	0
WRR_	GGRR	265	0	0
WRR_	GGRR	270	0	0
WRR_	GRWG	369	0	0
WRR_	GGRR	378	0	0
WRR_	YGWW	521	0	0
WRR_	YGWW	528	0	0
WRR_	G_GW	533	0	0
WRR_	G_GW	543	0	0
WRR_	YYGGmid	557	0	0
WRR_	Y_WY	563	0	0
WRR_	GR_Y2	570	0	0
WRR_	GGRR	572	0	0
WRR_	GGRR	580	0	0
WRR_	YWGW	584	0	0
WRR_	G_GW	590	0	0
WRR_	GGRR	595	0	0
WRR_	G_GW	601	0	0
WRR_	G_GW	617	0	0
GGRR	WRR_	4	0	0

GGRR	YWGW	6	0	0
GGRR	GRWG	11	0	0
GGRR	WRR_	15	0	0
GGRR	GRWG	20	0	0
GGRR	WG_R	23	0	0
GGRR	WG_R	34	0	0
GGRR	____topleft	41	0	0
GGRR	WRR_	47	0	0
GGRR	YWGW	64	0	0
GGRR	G_GW	66	0	0
GGRR	WRR_	74	0	0
GGRR	WRR_	80	0	0
GGRR	YYGGmid	90	0	0
GGRR	Y_WY	174	0	0
GGRR	Y_WY	185	0	0
GGRR	YWGW	190	0	0
GGRR	WRR_	267	0	0
GGRR	Y_WY	280	0	0
GGRR	Y_WY	348	0	0
GGRR	YYGGmid	360	0	0
GGRR	YWGW	375	0	0
GGRR	WRR_	379	0	0
GGRR	____topleft	390	0	0
GGRR	YWW_	401	0	0
GGRR	WRBB	413	0	0
GGRR	GR__	424	0	0
GGRR	GR_Y2	425	0	0
GGRR	G_W_	434	0	0
GGRR	_WGG	437	0	0
GGRR	G_GW	443	0	0
GGRR	YGWW	462	0	0
GGRR	YGWW	468	0	0
GGRR	YWWW	474	0	0
GGRR	_W__	567	0	0
GGRR	WRR_	572	0	0
GGRR	WRR_	587	0	0
GGRR	WRR_	596	0	0
GGRR	WRR_	607	0	0
GGRR	WRR_	619	0	0
GGRR	Y_WY	692	0	0
GGRR	Y_WY	699	0	0
GGRR	Y_WY	805	0	0
GGRR	Y_WY	827	0	0
GGRR	WG_R	844	0	0
GGRR	Y_WY	853	0	0
GGRR	YY_W	868	0	0
GGRR	Y_WY	877	0	0
GGRR	Y_WY	889	0	0
GGRR	WG_R	904	0	0

GGRR	_W__	911	0	0
GGRR	Y_WY	926	0	0
GGRR	_W__	940	0	0
GGRR	G_GW	1017	0	0
GGRR	Y__W	1034	0	0
GGRR	G_GW	1060	0	0
GGRR	G_GW	1072	0	0
GGRR	_W__	1081	0	0
GGRR	_W__	1120	0	0
GGRR	_W__	1148	0	0
GGRR	YYGGmid	1167	0	0
GGRR	____topleft	1233	0	0
GGRR	____topleft	1290	0	0
GGRR	WWBG	1316	0	0
GGRR	Y__W	1350	0	0
GGRR	G_GW	1375	0	0
G_GW	YWGW	28	0	0
G_GW	WG_R	44	0	0
G_GW	Y__W	53	0	0
G_GW	YWGW	127	0	0
G_GW	Y__W	134	0	0
G_GW	YWGW	154	0	0
G_GW	____topleft	283	0	0
G_GW	Y__W	294	0	0
G_GW	Y__W	360	0	0
G_GW	YGWW	361	0	0
G_GW	Y__W	372	0	0
G_GW	Y__W	405	0	0
G_GW	Y__W	448	0	0
G_GW	YY_W	466	0	0
G_GW	GGRR	497	0	0
G_GW	_W__	502	0	0
G_GW	YGWW	509	0	0
G_GW	YGWW	518	0	0
G_GW	WRR_	530	0	0
G_GW	WRR_	586	0	0
G_GW	WRR_	603	0	0
G_GW	WRR_	617	0	0
G_GW	_W__	861	0	0
G_GW	YWGW	873	0	0
G_GW	_W__	897	0	0
G_GW	YWGW	920	0	0
G_GW	YY_W	993	0	0
G_GW	Y__W	1033	0	0
G_GW	Y__W	1044	0	0
G_GW	GGRR	1063	0	0
G_GW	GGRR	1074	0	0
G_GW	YWGW	1127	0	0
G_GW	WWBG	1197	0	0

G_GW	YWGW	1208	0	0
G_GW	WWBG	1211	0	0
G_GW	_W__	1220	0	0
G_GW	_R__	1274	0	0
G_GW	_WGG	1295	0	0
G_GW	GGRR	1309	0	0
G_GW	____topleft	1374	0	0
Y__W	G_GW	448	0	0
Y__W	G_GW	1044	0	0
YYGGmid	Y_WY	13	0	0
YYGGmid	G_GW	29	0	0
YYGGmid	G_R_	37	0	0
YYGGmid	WG_R	40	0	0
YYGGmid	WRBB	56	0	0
YYGGmid	Y_WY	66	0	0
YYGGmid	GRWG	79	0	0
YYGGmid	WRBB	110	0	0
YYGGmid	WRBB	223	0	0
YYGGmid	WRBB	411	0	0
YYGGmid	WRR_	557	0	0
YYGGmid	WRBB	580	0	0
YYGGmid	WRBB	585	0	0
YYGGmid	WRWR	737	0	0
YYGGmid	GGRR	797	0	0
YYGGmid	WRWR	818	0	0
YYGGmid	WRRY	821	0	0
YYGGmid	GGRR	854	0	0
YYGGmid	WGWB	869	0	0
YYGGmid	WRBB	878	0	0
YYGGmid	WG_R	918	0	0
YYGGmid	GRWG	924	0	0
YYGGmid	WG_R	951	0	0
YYGGmid	WG_R	960	0	0
YYGGmid	WGGB	1031	0	0
YYGGmid	Y_WY	1140	0	0
YYGGmid	YY_W	1147	0	0
YYGGmid	_W__	1152	0	0
YYGGmid	YWGW	1184	0	0
YYGGmid	WG_R	1197	0	0
YYGGmid	_WGG	1204	0	0
YYGGmid	_W__	1242	0	0
YYGGmid	YWGW	1261	0	0
YYGGmid	_WGG	1285	0	0
YYGGmid	WRR_	1340	0	0
YYGGmid	GWRG	1366	0	0
YYGGmid	GWRG	1373	0	0
YYGGmid	__W_	1438	0	0
Y_WY	WRBB	0	0	0
Y_WY	YYGGmid	7	0	0

Y_WY	WRBB	18	0	0
Y_WY	WRBB	38	0	0
Y_WY	WRBB	50	0	0
Y_WY	GRWG	102	0	0
Y_WY	YWGW	170	0	0
Y_WY	GGRR	186	0	0
Y_WY	WG_R	191	0	0
Y_WY	WG_R	216	0	0
Y_WY	WG_R	229	0	0
Y_WY	YWGW	304	0	0
Y_WY	GGRR	351	0	0
Y_WY	____topleft	358	0	0
Y_WY	GR__	412	0	0
Y_WY	GGRR	453	0	0
Y_WY	GR__	464	0	0
Y_WY	YGWW	473	0	0
Y_WY	WG_R	523	0	0
Y_WY	WRBB	526	0	0
Y_WY	____pale	567	0	0
Y_WY	YGWW	672	0	0
Y_WY	WG_R	688	0	0
Y_WY	GGRR	702	0	0
Y_WY	WG_R	727	0	0
Y_WY	_W__	803	0	0
Y_WY	GGRR	807	0	0
Y_WY	GGRR	854	0	0
Y_WY	_W__	860	0	0
Y_WY	GGRR	881	0	0
Y_WY	WG_R	882	0	0
Y_WY	WRBB	900	0	0
Y_WY	WG_R	914	0	0
Y_WY	GGRR	928	0	0
Y_WY	_W__	980	0	0
Y_WY	WG_R	997	0	0
Y_WY	WG_R	1076	0	0
Y_WY	WG_R	1110	0	0
Y_WY	_W__	1179	0	0
Y_WY	____topleft	1211	0	0
Y_WY	WG_R	1230	0	0
Y_WY	WG_R	1258	0	0
Y_WY	WG_R	1315	0	0
Y_WY	WG_R	1383	0	0
WRBB	Y_WY	0	0	0
WRBB	Y_WY	24	0	0
WRBB	Y_WY	35	0	0
WRBB	YYGGmid	517	0	0
WRBB	YYGGmid	578	0	0
WRBB	YYGGmid	580	0	0
WRBB	YYGGmid	715	0	0

WRBB	YWWW	722	0	0
WRBB	WGGB	826	0	0
WRBB	WG_R	868	0	0
WRBB	WG_R	870	0	0
WRBB	____corner	910	0	0
WRBB	____corner	965	0	0
WRBB	GR__	1013	0	0
WRBB	GR_Y2	1081	0	0
WRBB	GR_Y2	1104	0	0
WRBB	_WGG	1122	0	0
WRBB	WYGG	1154	0	0
WRBB	WYGG	1264	0	0
WRBB	WGBB	1266	0	0
WRBB	WGBB	1305	0	0
WRBB	RWWG	1314	0	0
WRBB	WBGW	1324	0	0
WRBB	RWWG	1327	0	0
WRBB	GR__	1364	0	0
__W_	GRWG	151	0	0
__W_	____topleft	155	0	0
__W_	WRBB	168	0	0
__W_	_W__	230	0	0
__W_	_W__	264	0	0
__W_	WG_R	374	0	0
__W_	WG_R	430	0	0
__W_	YYGGmid	505	0	0
__W_	Y_WY	528	0	0
__W_	YGWW	546	0	0
__W_	WRR_	558	0	0
__W_	GGRR	575	0	0
__W_	GGRR	596	0	0
__W_	YGWW	622	0	0
__W_	GGRR	631	0	0
__W_	YGWW	634	0	0
__W_	YGWW	646	0	0
__W_	_W__	831	0	0
__W_	_W__	841	0	0
__W_	_R__	860	0	0
__W_	YY_W	990	0	0
__W_	Q	1183	0	0
__W_	Q	1190	0	0
__W_	Q	1200	0	0
__W_	GGWY	1201	0	0
__W_	_R__	1219	0	0
__W_	Q	1233	0	0
__W_	WRRY	1400	0	0
__W_	WRRY	1425	0	0
GGWY	YYGG	35	0	0
GGWY	GRWG	190	0	0

GGWY	GRWG	192	0	0
GGWY	WRWR	875	0	0
GGWY	_WWY	908	0	0
GGWY	GG_W	960	0	0
GGWY	YYGG	975	0	0
GGWY	YYGG	980	0	0
GGWY	GGWW	1008	0	0
GGWY	GGRY	1015	0	0
GGWY	GG_W	1024	0	0
GGWY	WRRY	1026	0	0
GGWY	GBGR	1033	0	0
GGWY	GWRG	1040	0	0
GGWY	GWRG	1047	0	0
GGWY	GWRG	1052	0	0
GGWY	YYRB	1055	0	0
GGWY	GBGR	1060	0	0
GGWY	WRRY	1066	0	0
GGWY	Q	1074	0	0
GGWY	WRRY	1077	0	0
GGWY	Q	1086	0	0
GGWY	W___	1092	0	0
GGWY	YYGG	1095	0	0
GGWY	GGWW	1113	0	0
GGWY	YYWR	1118	0	0
GGWY	YYGG	1125	0	0
GGWY	_WWW	1158	0	0
GGWY	YY_R	1167	0	0
GGWY	_WYW	1170	0	0
GGWY	__W_	1200	0	0
GGWY	Q	1213	0	0
GGWY	WGWB	1225	0	0
GGWY	_W__	1231	0	0
GGWY	GGRR	1232	0	0
GGWY	WGBB	1241	0	0
GGWY	____almost	1249	0	0
GGWY	_W_Y	1267	0	0
GGWY	_W_Y	1339	0	0
GGWY	RWWG	1365	0	0
GGWY	_WYW	1411	0	0
GGWY	_WYW	1434	0	0
GGWY	____topright	1435	0	0
G_W_	YY_W	40	0	0
G_W_	YY_W	68	0	0
G_W_	YY_W	99	0	0
G_W_	YY_W	251	0	0
G_W_	WBGW	312	0	0
G_W_	WBGW	477	0	0
G_W_	GR__	601	0	0
G_W_	GR__	813	0	0

G_W_	WBGW	1239	0	0
G_W_	WBGW	1245	0	0
G_W_	WBGW	1327	0	0
G_W_	WBGW	1360	0	0
G_W_	WRBB	1362	0	0
G_W_	GR__	1377	0	0
G_W_	GGWY	1393	0	0
G_W_	_W_Y	1407	0	0
WBGW	G_W_	44	0	0
WBGW	G_W_	102	0	0
WBGW	G_W_	333	0	0
WBGW	YY_W	954	0	0
WBGW	WYGG	998	0	0
WBGW	_WGG	1042	0	0
WBGW	____almost	1120	0	0
WBGW	G_W_	1230	0	0
WBGW	G_W_	1245	0	0
WBGW	G_W_	1248	0	0
WBGW	WRBB	1367	0	0
YY_W	G_W_	5	0	0
YY_W	G_W_	83	0	0
YY_W	G_W_	147	0	0
YY_W	G_W_	248	0	0
YY_W	G_W_	389	0	0
YY_W	G_W_	638	0	0
YY_W	G_W_	720	0	0
YY_W	G_W_	773	0	0
YY_W	G_W_	854	0	0
YY_W	G_W_	905	0	0
YY_W	GR__	954	0	0
YY_W	WRBB	967	0	0
YY_W	WG_R	979	0	0
YY_W	YYGGmid	981	0	0
YY_W	G_GW	995	0	0
YY_W	GYYY	1011	0	0
YY_W	_RYG	1015	0	0
YY_W	G___small	1024	0	0
YY_W	G___big	1027	0	0
YY_W	GGWY	1156	0	0
YY_W	GGWY	1333	0	0
YY_W	GGWY	1438	0	0
GR__	Y_WY	415	0	0
GR__	GGRR	436	0	0
GR__	Y_WY	448	0	0
GR__	Y_WY	464	0	0
GR__	_WGG	480	0	0
GR__	G_GW	491	0	0
GR__	WRR_	572	0	0
GR__	G_W_	578	0	0

GR__	G_W_	587	0	0
GR__	G_W_	960	0	0
GR__	_WGG	1082	0	0
GR__	_WGG	1088	0	0
GR__	_WGG	1103	0	0
GR__	WRBB	1164	0	0
GR__	WRBB	1170	0	0
GR__	WRBB	1290	0	0
GR__	WYGG	1304	0	0
GR__	_W__	1310	0	0
GR__	WRBB	1317	0	0
GR__	G_W_	1335	0	0
GR__	WRBB	1365	0	0
GR__	G_W_	1370	0	0
GR__	G_W_	1386	0	0
GR__	Y_WY	1410	0	0
GR__	WG_R	1418	0	0
YGWW	WG_R	68	0	0
YGWW	WG_R	71	0	0
YGWW	WG_R	87	0	0
YGWW	WG_R	97	0	0
YGWW	WG_R	109	0	0
YGWW	WG_R	154	0	0
YGWW	GR__	212	0	0
YGWW	GR__	227	0	0
YGWW	GR__	270	0	0
YGWW	WG_R	349	0	0
YGWW	G_GW	359	0	0
YGWW	Y_WY	369	0	0
YGWW	WG_R	374	0	0
YGWW	_W__	422	0	0
YGWW	_W__	428	0	0
YGWW	WG_R	437	0	0
YGWW	YWGW	451	0	0
YGWW	G_GW	455	0	0
YGWW	_W__	461	0	0
YGWW	_W__	467	0	0
YGWW	_W__	484	0	0
YGWW	YWGW	500	0	0
YGWW	WRR_	522	0	0
YGWW	YWGW	534	0	0
YGWW	WG_R	541	0	0
YGWW	YYGGmid	545	0	0
YGWW	GGRR	548	0	0
YGWW	__W_	622	0	0
YGWW	__W_	632	0	0
YGWW	YYGGmid	651	0	0
YGWW	YYGGmid	660	0	0
YGWW	Y_WY	671	0	0

YGWW	Y_WY	737	0	0
YGWW	__W_	740	0	0
YGWW	GGRR	741	0	0
YGWW	_W__	845	0	0
YGWW	____corner	937	0	0
YGWW	_WWY	1066	0	0
YGWW	G_GW	1149	0	0
YGWW	YWGW	1167	0	0
YGWW	RWGY	1187	0	0
YGWW	Y_WY	1200	0	0
YGWW	Q	1204	0	0
YGWW	_R__	1220	0	0
YGWW	____bm	1240	0	0
YGWW	_R__	1246	0	0
YGWW	RWGY	1255	0	0
YGWW	GRBR	1274	0	0
YGWW	GGRY	1284	0	0
YGWW	_Y__	1296	0	0
YGWW	__BB	1300	0	0
YGWW	____right	1315	0	0
YGWW	__BB	1346	0	0
YGWW	__BB	1370	0	0
YGWW	_Y__	1374	0	0
YGWW	_WYG	1377	0	0
YGWW	_WYG	1382	0	0
YGWW	_WYG	1391	0	0
YGWW	W___	1407	0	0
YGWW	YYRB	1413	0	0
YGWW	GGWY	1422	0	0
YGWW	YYRB	1432	0	0
YGWW	_W__	1438	0	0
GR_Y	____topleft	140	0	0
GR_Y	_W__	144	0	0
GR_Y	____topleft	156	0	0
GR_Y	WG_R	170	0	0
GR_Y	____pale	200	0	0
GR_Y	____pale	243	0	0
GR_Y	GGRR	426	0	0
GR_Y	____pale	530	0	0
GR_Y	GR__	553	0	0
GR_Y	____pale	584	0	0
GR_Y	WG_R	595	0	0
GR_Y	WG_R	609	0	0
GR_Y	WG_R	624	0	0
GR_Y	GR__	676	0	0
GR_Y	WG_R	865	0	0
GR_Y	WBGW	912	0	0
GR_Y	RWGY	963	0	0
GR_Y	WGBB	1072	0	0

GR_Y	WRBB	1082	0	0
GR_Y	WRBB	1097	0	0
GR_Y	WRBB	1105	0	0
GR_Y	GGWY	1242	0	0
GR_Y	WYGG	1290	0	0
_____pale	WGBB	206	0	0
_____pale	Y_WY	568	0	0
_____pale	WGBB	623	0	0
_____pale	Y_WY	630	0	0
_____pale	WRBB	724	0	0
_____pale	WGWB	873	0	0
_____pale	_____corner	912	0	0
_____pale	_WWY	927	0	0
_____pale	_WWY	945	0	0
_____pale	WRRY	954	0	0
_____pale	YYGG	975	0	0
_____pale	GGWY	997	0	0
_____pale	_R__	1025	0	0
_____pale	Q	1032	0	0
_____pale	Q	1034	0	0
_____pale	Q	1070	0	0
_____pale	GG_W	1113	0	0
_____pale	GGWW	1123	0	0
_____pale	RWGY	1145	0	0
_____pale	_WYW	1153	0	0
_____pale	_R__	1158	0	0
_____pale	GR_Y2	1162	0	0
_____pale	_W__	1176	0	0
_____pale	YYRB	1182	0	0
_____pale	GBGR	1186	0	0
_____pale	GGRY	1210	0	0
_____pale	GBGW	1213	0	0
_____pale	WBYG	1238	0	0
_____pale	GGRY	1242	0	0
_____pale	YYWR	1248	0	0
_____pale	GGRY	1258	0	0
_____pale	YYWR	1275	0	0
_____pale	YYWR	1286	0	0
_____pale	GGRY	1294	0	0
_____pale	YYWR	1309	0	0
_____pale	GGRY	1332	0	0
_____pale	YYWR	1354	0	0
_____pale	_____bot	1358	0	0
_WGG	WYGG	913	0	0
_WGG	WBGW	914	0	0
_WGG	RWGY	979	0	0
_WGG	WYGG	990	0	0
_WGG	WRBB	995	0	0
_WGG	RWGY	1000	0	0

_WGG	RWGY	1008	0	0
_WGG	WYGG	1013	0	0
_WGG	WYGG	1016	0	0
_WGG	GR__	1022	0	0
_WGG	WYGG	1040	0	0
_WGG	RWWG	1042	0	0
_WGG	WYGG	1046	0	0
_WGG	____topright	1049	0	0
_WGG	WGBB	1077	0	0
_WGG	GR__	1080	0	0
_WGG	GR__	1086	0	0
_WGG	Y_WY	1089	0	0
_WGG	Y_WY	1094	0	0
_WGG	WG_R	1106	0	0
_WGG	Y_WY	1109	0	0
_WGG	____topleft	1118	0	0
_WGG	WRBB	1122	0	0
_WGG	G_W_	1157	0	0
_WGG	G_W_	1167	0	0
_WGG	YYGGmid	1200	0	0
_WGG	Y_WY	1206	0	0
_WGG	YYGGmid	1226	0	0
_WGG	YYGGmid	1235	0	0
_WGG	_W__	1240	0	0
_WGG	WG_R	1261	0	0
_WGG	____topleft	1272	0	0
_WGG	G_GW	1287	0	0
_WGG	GBGR	1373	0	0
WYGG	WGWB	897	0	0
WYGG	_WGG	913	0	0
WYGG	____almost	934	0	0
WYGG	WBGW	949	0	0
WYGG	____almost	989	0	0
WYGG	WBGW	991	0	0
WYGG	_WGG	998	0	0
WYGG	RWGY	1000	0	0
WYGG	_WGG	1013	0	0
WYGG	____almost	1029	0	0
WYGG	RWGY	1033	0	0
WYGG	RWGY	1044	0	0
WYGG	WGWB	1051	0	0
WYGG	GGW_	1063	0	0
WYGG	YYGW	1068	0	0
WYGG	GGW_	1110	0	0
WYGG	GGGG	1123	0	0
WYGG	RWGY	1137	0	0
WYGG	RWGY	1148	0	0
WYGG	WRBB	1095	0	0
WYGG	WRBB	1170	0	0

WYGG	GR__	1301	0	0
WYGG	_W__	1388	0	0
WYGG	YYGGmid	1396	0	0
WYGG	G_W_	1406	0	0
_____topright	_____almost	985	0	0
_____topright	_____almost	1054	0	0
_____topright	_W_Y	1269	0	0
_____topright	_W_Y	1280	0	0
_____topright	_W_Y	1324	0	0
_____topright	_W_Y	1345	0	0
_____topright	_____almost	1412	0	0
_____almost	GGW_	999	0	0
_____almost	RWWG	1026	0	0
_____almost	GGW_	1030	0	0
_____almost	RWWG	1034	0	0
_____almost	RWWG	1039	0	0
_____almost	RWWG	1046	0	0
_____almost	WYGG	1050	0	0
_____almost	WGWB	1057	0	0
_____almost	YYGW	1064	0	0
_____almost	WGBB	1075	0	0
_____almost	_____topright	1109	0	0
_____almost	WBGW	1114	0	0
_____almost	WBGW	1224	0	0
_____almost	RWWG	1231	0	0
_____almost	RWWG	1250	0	0
_____almost	GGWY	1258	0	0
_____almost	_W_Y	1267	0	0
_____almost	_W_Y	1275	0	0
_____almost	GGWY	1298	0	0
_____almost	_W_Y	1342	0	0
_____almost	RWWG	1360	0	0
_____almost	_____topright	1419	0	0
_____almost	_WYW	1432	0	0
RWGY	WGBB	931	0	0
RWGY	WGBB	945	0	0
RWGY	WRBB	966	0	0
RWGY	_WGG	989	0	0
RWGY	_WGG	992	0	0
RWGY	WYGG	1000	0	0
RWGY	RWWG	1004	0	0
RWGY	WGWB	1010	0	0
RWGY	WGWB	1025	0	0
RWGY	WGGB	1098	0	0
RWGY	WGGB	1105	0	0
RWGY	G_R_	1154	0	0
RWGY	WG_R	1160	0	0
RWGY	_R__	1165	0	0
RWGY	GR_Y2	1175	0	0

RWGY	WRWR	1197	0	0
RWGY	Q	1308	0	0
RWWG	GGYW	1019	0	0
RWWG	GGGG	1027	0	0
RWWG	_____almost	1033	0	0
RWWG	_____almost	1040	0	0
RWWG	GGGG	1057	0	0
RWWG	GGGG	1062	0	0
RWWG	_____almost	1080	0	0
RWWG	GGGG	1114	0	0
RWWG	_____almost	1229	0	0
RWWG	_____almost	1250	0	0
RWWG	YYGG	1256	0	0
RWWG	WRBB	1312	0	0
RWWG	WRBB	1328	0	0
RWWG	_W_Y	1362	0	0
RWWG	_W_Y	1370	0	0
RWWG	WGBB	1401	0	0
RWWG	GGGG	1412	0	0
RWWG	_WYW	1417	0	0
RWWG	G___	1426	0	0
RWWG	WGBB	1438	0	0
GGW_	_____almost	993	0	0
GGW_	_____topright	1030	0	0
GGW_	YYGW	1041	0	0
GGW_	YYGW	1053	0	0
GGW_	WYGG	1070	0	0
GGW_	GGGG	1078	0	0
GGW_	GGGG	1081	0	0
GGW_	YYGW	1104	0	0
GGW_	_W_Y	1206	0	0
GGW_	YYGW	1210	0	0
GGW_	GBG_	1213	0	0
GGW_	GBG_	1222	0	0
GGW_	_WYW	1230	0	0
GGW_	WBGG	1238	0	0
GGW_	WBGG	1265	0	0
GGW_	WBGG	1279	0	0
GGW_	GBG_	1342	0	0
GGW_	YY_R	1357	0	0
YYGW	GGW_	1034	0	0
YYGW	GGW_	1041	0	0
YYGW	_____almost	1045	0	0
YYGW	GGGG	1050	0	0
YYGW	GGGG	1054	0	0
YYGW	GGW_	1057	0	0
YYGW	_WYW	1085	0	0
YYGW	WBGW	1106	0	0
YYGW	G___	1164	0	0

YYGW	G_R_	1295	0	0
YYGW	WGBB	1304	0	0
YYGW	GGW_	1307	0	0
YYGW	WBGG	1318	0	0
YYGW	GGW_	1324	0	0
YYGW	_WYW	1341	0	0
YYGW	_WYW	1351	0	0
YYGW	GGGG	1417	0	0
YYGW	GGGG	1435	0	0
GGGG	GGW_	1046	0	0
GGGG	GGYW	1052	0	0
GGGG	YYGW	1057	0	0
GGGG	RWWG	1062	0	0
GGGG	GGYW	1070	0	0
GGGG	GGW_	1079	0	0
GGGG	GGWY	1262	0	0
GGGG	_WYW	1359	0	0
GGGG	_WYW	1368	0	0
GGGG	_W_Y	1392	0	0
GGGG	RWWG	1418	0	0
GGGG	RWWG	1425	0	0
GGYW	WGBB	1032	0	0
GGYW	WGWB	1064	0	0
GGYW	WGWB	1252	0	0
GGYW	WGWB	1262	0	0
GGYW	WGBB	1278	0	0
GGYW	WGWB	1290	0	0
GGYW	WBGG	1308	0	0
GGYW	G___	1393	0	0
GGYW	WGWB	1415	0	0
WGBB	GGYW	942	0	0
WGBB	GGYW	952	0	0
WGBB	WGWB	1065	0	0
WGBB	GR_Y2	1070	0	0
WGBB	WRBB	1072	0	0
WGBB	WGWB	1106	0	0
WGBB	WGWB	1201	0	0
WGBB	GGYW	1216	0	0
WGBB	WYGG	1240	0	0
WGBB	WYGG	1306	0	0
WGBB	GGWY	1315	0	0
_WYW	GGYW	1085	0	0
_WYW	YYGW	1095	0	0
_WYW	WGBB	1121	0	0
_WYW	WGGB	1130	0	0
_WYW	_R__	1148	0	0
_WYW	____pale	1151	0	0
_WYW	____pale	1154	0	0
_WYW	WRWR	1159	0	0

_WYW	GGWY	1170	0	0
_WYW	G___	1200	0	0
_WYW	GBG_	1215	0	0
_WYW	GGW_	1228	0	0
_WYW	WBGW	1303	0	0
_WYW	YYGW	1343	0	0
_WYW	YYGW	1351	0	0
_WYW	GGGG	1360	0	0
_WYW	____almost	1402	0	0
_WYW	RWWG	1406	0	0
_WYW	G___	1425	0	0
_WYW	____almost	1434	0	0
_W__	____topleft	155	0	0
_W__	____topleft	170	0	0
_W__	WG_R	192	0	0
_W__	__W_	260	0	0
_W__	WG_R	366	0	0
_W__	WG_R	372	0	0
_W__	YGWW	395	0	0
_W__	YGWW	407	0	0
_W__	YWGW	414	0	0
_W__	____topleft	420	0	0
_W__	GGRR	566	0	0
_W__	Y__W	1010	0	0
_W__	____topleft	1031	0	0
_W__	YWGW	1055	0	0
_W__	YWGW	1063	0	0
_W__	Y__W	1116	0	0
_W__	Y_WY	1136	0	0
_W__	Y_WY	1149	0	0
_W__	GR__	1152	0	0
_W__	_WGG	1160	0	0
_W__	Y_WY	1180	0	0
_W__	Y_WY	1210	0	0
_W__	WG_R	1225	0	0
_W__	YYGW	1226	0	0
_W__	YYGW	1230	0	0
_W__	YYGW	1228	0	0
_W__	_WGG	1254	0	0
_W__	G_W_	1262	0	0
_W__	GR__	1308	0	0
_W__	GGRR	1353	0	0
_W__	GR__	1380	0	0
_W__	WYGG	1388	0	0
_W__	Y_WY	1393	0	0
_W__	GR__	1395	0	0
_W__	GR__	1409	0	0
WGGB	_WWY	734	0	0
WGGB	WRBB	826	0	0

WGGB	____pale	845	0	0
WGGB	WGWB	882	0	0
WGGB	GR_Y2	931	0	0
WGGB	GGRR	942	0	0
WGGB	____pale	947	0	0
WGGB	YYGGmid	970	0	0
WGGB	_WWY	1002	0	0
WGGB	_WWY	1018	0	0
WGGB	GR_Y2	1026	0	0
WGGB	YYGGmid	1032	0	0
WGGB	GGRR	1095	0	0
WGGB	RWGY	1098	0	0
WGGB	YYGGmid	1114	0	0
WGGB	_WYW	1132	0	0
WGGB	GR_Y2	1183	0	0
WGGB	WGWB	1197	0	0
WGGB	WGBB	1198	0	0
WGGB	GR_Y2	1244	0	0
WGGB	GGRR	1274	0	0
WGGB	GGRR	1310	0	0
WGGB	WRWR	1411	0	0
_WWY	YWWW	696	0	0
_WWY	WGGB	734	0	0
_WWY	Q	738	0	0
_WWY	GRGY	833	0	0
_WWY	YY_R	841	0	0
_WWY	GRGY	870	0	0
_WWY	GGWW	884	0	0
_WWY	WRWR	895	0	0
_WWY	____corner	914	0	0
_WWY	GRWG	950	0	0
_WWY	WGGB	994	0	0
_WWY	WGGB	1005	0	0
_WWY	_WYW	1018	0	0
_WWY	_R__	1026	0	0
_WWY	_R__	1031	0	0
_WWY	YGWW	1039	0	0
_WWY	YGWW	1050	0	0
_WWY	____corner	1063	0	0
_WWY	_R__	1079	0	0
_WWY	WRRY	1081	0	0
_WWY	YWW_	1084	0	0
_WWY	WRRY	1091	0	0
_WWY	__W_	1110	0	0
_WWY	YWW_	1173	0	0
_WWY	WRRY	1234	0	0
_WWY	YYYY	1262	0	0
_WWY	G___small	1342	0	0
_WWY	WBGG	1410	0	0

YWWW	WRBB	716	0	0
YWWW	____pale	760	0	0
YWWW	YYGGmid	783	0	0
YWWW	YYGGmid	789	0	0
YWWW	YYGGmid	808	0	0
YWWW	WRRY	809	0	0
YWWW	____pale	818	0	0
YWWW	GGRR	829	0	0
YWWW	__W_	831	0	0
YWWW	Y_WY	857	0	0
YWWW	G_GW	862	0	0
YWWW	GGRR	922	0	0
YWWW	____corner	939	0	0
YWWW	_WWY	1022	0	0
YWWW	_WYW	1054	0	0
YWWW	_WWY	1114	0	0
YWWW	__W_	1120	0	0
YWWW	YWGW	1150	0	0
YWWW	YWGW	1165	0	0
Q	G___	945	0	0
Q	G___	980	0	0
Q	GR_Y2	990	0	0
Q	G___	1000	0	0
Q	YY_R	1005	0	0
Q	Y___	1014	0	0
Q	GGWW	1027	0	0
Q	____pale	1037	0	0
Q	GGWW	1063	0	0
Q	GG_W	1069	0	0
Q	GGWY	1074	0	0
Q	WRRY	1077	0	0
Q	GR_Y2	1079	0	0
Q	_R__	1084	0	0
Q	_WYW	1090	0	0
Q	WRRY	1159	0	0
Q	YGWW	1172	0	0
Q	WWBG	1178	0	0
Q	_WYW	1184	0	0
Q	_R__	1191	0	0
Q	YGWW	1203	0	0
Q	_WYW	1206	0	0
Q	GGWY	1215	0	0
Q	WRRY	1256	0	0
Q	WRWR	1283	0	0
Q	_R__	1286	0	0
Q	RWGY	1306	0	0
Q	GGWW	1321	0	0
Q	YY_R	1348	0	0
Q	YY_R	1355	0	0

G___	Q	944	0	0
G___	GRGY	974	0	0
G___	Q	982	0	0
G___	GRGY	1137	0	0
G___	YYGW	1197	0	0
G___	YYGG	1201	0	0
G___	YYGW	1206	0	0
G___	GRGY	1216	0	0
G___	GRGY	1330	0	0
G___	WBGG	1334	0	0
GBG_	YYGW	1102	0	0
GBG_	YYGW	1108	0	0
GBG_	GG_W	1156	0	0
GBG_	WBGG	1179	0	0
GBG_	YYGW	1207	0	0
GBG_	YYGW	1219	0	0
GBG_	GG_W	1222	0	0
GBG_	GG_W	1226	0	0
GBG_	WGWB	1236	0	0
GBG_	WGWB	1250	0	0
GBG_	WGWB	1305	0	0
GBG_	WGWB	1325	0	0
GBG_	YYGW	1360	0	0
_W_Y	WBGG	941	0	0
_W_Y	WBGG	954	0	0
_W_Y	WBGG	1037	0	0
_W_Y	WBGG	1070	0	0
_W_Y	GGGR	1074	0	0
_W_Y	YY_R	1078	0	0
_W_Y	WBGG	1085	0	0
_W_Y	_____right	1110	0	0
_W_Y	WBGG	1138	0	0
_W_Y	GG_W	1205	0	0
_W_Y	RWWG	1263	0	0
_W_Y	_____almost	1266	0	0
_W_Y	WBGW	1273	0	0
_W_Y	_____topright	1295	0	0
_W_Y	_____topright	1338	0	0
_W_Y	GGGG	1353	0	0
_W_Y	RWWG	1363	0	0
_W_Y	RWWG	1368	0	0
_W_Y	_____almost	1372	0	0
_W_Y	GGGG	1387	0	0
_W_Y	WYGG	1402	0	0
_W_Y	GR__	1408	0	0
_W_Y	_____almost	1410	0	0
_W_Y	GR__	1414	0	0
_W_Y	WBGW	1426	0	0
_W_Y	_____topright	1433	0	0

GR_Y2	YY_R	940	0	0
GR_Y2	Q	942	0	0
GR_Y2	YY_R	954	0	0
GR_Y2	Q	1000	0	0
GR_Y2	WRWR	1015	0	0
GR_Y2	_WWY	1022	0	0
GR_Y2	WGGB	1026	0	0
GR_Y2	____pale	1041	0	0
GR_Y2	_R__	1070	0	0
GR_Y2	Q	1078	0	0
GR_Y2	Q	1084	0	0
GR_Y2	_R__	1099	0	0
GR_Y2	WGGB	1102	0	0
GR_Y2	WGGB	1109	0	0
GR_Y2	_WWY	1111	0	0
GR_Y2	_R__	1176	0	0
GR_Y2	____brood	1184	0	0
GR_Y2	WRRY	1207	0	0
GR_Y2	YY_R	1312	0	0
GR_Y2	YGWY	1315	0	0
GR_Y2	GBGW	1321	0	0
GR_Y2	YGWY	1356	0	0
GR_Y2	YYRB	1372	0	0
GR_Y2	YYY_	1390	0	0
GR_Y2	YYRB	1405	0	0
GR_Y2	YGWW	1415	0	0
GR_Y2	YGWY	1430	0	0
GR_Y2	YGWW	1437	0	0
Y___	YY_R	903	0	0
Y___	GR_Y2	909	0	0
Y___	YY_R	916	0	0
Y___	YYGGright	937	0	0
Y___	YY_R	947	0	0
Y___	GRWG	969	0	0
Y___	GGWY	1192	0	0
Y___	Q	1339	0	0
Y___	Q	1345	0	0
Y___	Q	1435	0	0
Y___	YY_R	1438	0	0
YY_R	YYRG	840	0	0
YY_R	YYY_	916	0	0
YY_R	YYY_	932	0	0
YY_R	YYGGright	946	0	0
YY_R	GR_Y2	951	0	0
YY_R	GR_Y2	956	0	0
YY_R	GR_Y2	960	0	0
YY_R	YYGGright	1186	0	0
YY_R	GBG_	1210	0	0
YY_R	GR_Y2	1232	0	0

YY_R	GR_Y2	1302	0	0
YY_R	G___	1311	0	0
YY_R	YYGGright	1404	0	0
YY_R	Q	1409	0	0
YY_R	Q	1430	0	0
YY_R	Y___	1438	0	0
GRGY	_WWY	763	0	0
GRGY	_WWY	813	0	0
GRGY	_WWY	835	0	0
GRGY	_WWY	870	0	0
GRGY	GRWG	958	0	0
GRGY	GRWG	970	0	0
GRGY	Q	1022	0	0
GRGY	Q	1030	0	0
GRGY	GGRR	1044	0	0
GRGY	G___	1218	0	0
GRGY	G___	1248	0	0
GRGY	G___	1280	0	0
GRGY	YY_R	1304	0	0
GRGY	WBGG	1347	0	0
GRGY	GGWW	1398	0	0
GRGY	GGWW	1412	0	0
GGWW	WGWB	856	0	0
GGWW	GRBR	870	0	0
GGWW	GG_W	884	0	0
GGWW	YY__	1041	0	0
GGWW	YYGG	1046	0	0
GGWW	YY__	1059	0	0
GGWW	Q	1062	0	0
GGWW	Q	1071	0	0
GGWW	GG_W	1074	0	0
GGWW	GG_W	1079	0	0
GGWW	____pale	1122	0	0
GGWW	_R__	1272	0	0
GGWW	_R__	1278	0	0
GGWW	Y___	1312	0	0
GGWW	Q	1334	0	0
GGWW	Q	1345	0	0
GGWW	Q	1403	0	0
GGWW	GRGY	1422	0	0
GG_W	GGWW	869	0	0
GG_W	GGWW	885	0	0
GG_W	GGWY	963	0	0
GG_W	GGWY	969	0	0
GG_W	YYGG	976	0	0
GG_W	_R__	986	0	0
GG_W	GGRY	999	0	0
GG_W	GGWY	1023	0	0
GG_W	W___	1040	0	0

GG_W	Q	1061	0	0
GG_W	Q	1068	0	0
GG_W	GGWW	1075	0	0
GG_W	GGWW	1080	0	0
GG_W	GRBR	1214	0	0
GG_W	____brood	1222	0	0
GG_W	GRBR	1241	0	0
GG_W	RWGY	1260	0	0
GG_W	RWGY	1280	0	0
GG_W	_R__	1287	0	0
GG_W	WRRY	1318	0	0
GG_W	GWRG	1334	0	0
GG_W	WRWR	1415	0	0
GG_W	_R__	1438	0	0
GRBR	____bm	815	0	0
GRBR	GGWW	871	0	0
GRBR	GGWW	1207	0	0
GRBR	GG_W	1212	0	0
GRBR	GG_W	1218	0	0
GRBR	RWGY	1230	0	0
GRBR	RWGY	1266	0	0
GRBR	W___	1309	0	0
GRBR	YY_R	1434	0	0
YY__	WGWB	822	0	0
YY__	GRBR	862	0	0
YY__	_WYG	877	0	0
YY__	_WYG	887	0	0
YY__	_WYG	926	0	0
YY__	GGWW	1000	0	0
YY__	GGWY	1018	0	0
YY__	GGWW	1058	0	0
YY__	GRBR	1076	0	0
YY__	YYGGright	1089	0	0
YY__	YYGGright	1100	0	0
YY__	GYGG	1112	0	0
YY__	_WYG	1128	0	0
YY__	GYGG	1183	0	0
YY__	_WYG	1240	0	0
YY__	_WYG	1271	0	0
YY__	_WYG	1333	0	0
YY__	_WYG	1435	0	0
_WYG	GG_W	824	0	0
_WYG	YY__	848	0	0
_WYG	YY__	889	0	0
_WYG	GGRY	1073	0	0
_WYG	GGRY	1089	0	0
_WYG	GGRY	1097	0	0
_WYG	GYGG	1174	0	0
_WYG	____bot	1181	0	0

_WYG	YY__	1217	0	0
_WYG	YGWW	1298	0	0
_WYG	GGGR	1326	0	0
_WYG	YY__	1336	0	0
WBGG	GGW_	927	0	0
WBGG	_W_Y	940	0	0
WBGG	_W_Y	954	0	0
WBGG	____right	968	0	0
WBGG	_W_Y	1006	0	0
WBGG	_W_Y	1070	0	0
WBGG	_W_Y	1090	0	0
WBGG	GGGR	1206	0	0
WBGG	____right	1215	0	0
WBGG	GGGR	1227	0	0
WBGG	GGW_	1239	0	0
WBGG	_WYW	1241	0	0
WBGG	GGW_	1279	0	0
WBGG	GBG_	1282	0	0
WBGG	WGWB	1294	0	0
WBGG	_WYW	1302	0	0
WBGG	GGYW	1308	0	0
WBGG	WGBB	1322	0	0
WBGG	G___	1334	0	0
WBGG	WRWR	1353	0	0
WBGG	YYGGmid	1372	0	0
WBGG	WRRY	1395	0	0
WBGG	_WWY	1409	0	0
WBGG	YYYY	1430	0	0
WBGG	_WWY	1435	0	0
____right	GGW_	875	0	0
____right	GGW_	905	0	0
____right	WBGG	975	0	0
____right	YYGGright	1040	0	0
____right	GGGR	1081	0	0
____right	WBGG	1219	0	0
____right	YYGGright	1242	0	0
____right	YYGGright	1326	0	0
YYGGright	____right	863	0	0
YYGGright	GGGR	871	0	0
YYGGright	GGW_	880	0	0
YYGGright	GGW_	886	0	0
YYGGright	WBGG	985	0	0
YYGGright	GGGR	1005	0	0
YYGGright	WBGG	1018	0	0
YYGGright	GGW_	1045	0	0
YYGGright	_WWW	1068	0	0
YYGGright	__BB	1091	0	0
YYGGright	_Y__	1100	0	0
YYGGright	__BB	1102	0	0

YYGGright	YY__	1119	0	0
YYGGright	GGWY	1125	0	0
YYGGright	_WWW	1146	0	0
YYGGright	GGGR	1149	0	0
YYGGright	GGWY	1183	0	0
YYGGright	YY_R	1193	0	0
YYGGright	GGGR	1240	0	0
YYGGright	____right	1241	0	0
YYGGright	WBGG	1279	0	0
YYGGright	GGW_	1309	0	0
YYGGright	____right	1363	0	0
GRYY	Y___	941	0	0
GRYY	_WWW	975	0	0
GRYY	GGGR	999	0	0
GRYY	GGGR	1062	0	0
GRYY	Y___	1132	0	0
GRYY	YYGGright	1138	0	0
GRYY	Y___	1156	0	0
GRYY	_WWW	1265	0	0
GRYY	GGGR	1374	0	0
GRYY	YY_R	1388	0	0
GGGR	YYGGright	870	0	0
GGGR	GGW_	881	0	0
GGGR	WBGG	934	0	0
GGGR	WBGG	986	0	0
GGGR	GRYY	1001	0	0
GGGR	YYGGright	1006	0	0
GGGR	____right	1053	0	0
GGGR	GRYY	1063	0	0
GGGR	YYGGright	1197	0	0
GGGR	GRYY	1264	0	0
GGGR	_WWW	1288	0	0
GGGR	_WYG	1323	0	0
GGGR	_WYG	1326	0	0
GGGR	GRYY	1381	0	0
GGGR	YYRG	1425	0	0
_WWW	GGGR	846	0	0
_WWW	GRYY	856	0	0
_WWW	YYGGright	949	0	0
_WWW	GRYY	965	0	0
_WWW	GGGR	977	0	0
_WWW	GYGG	1009	0	0
_WWW	__BB	1018	0	0
_WWW	YYGGright	1069	0	0
_WWW	YY_R	1088	0	0
_WWW	YYGGright	1147	0	0
_WWW	GGWY	1157	0	0
_WWW	YYGGright	1169	0	0
_WWW	__BB	1224	0	0

_WWW	GYGG	1229	0	0
_WWW	Y___	1250	0	0
_WWW	GYGG	1334	0	0
_WWW	__BB	1349	0	0
_WWW	GRYY	1378	0	0
GYGG	GGW_	801	0	0
GYGG	_____corner	820	0	0
GYGG	_Y__	848	0	0
GYGG	_____corner	859	0	0
GYGG	__BB	883	0	0
GYGG	_Y__	886	0	0
GYGG	__BB	1008	0	0
GYGG	GGW_	1038	0	0
GYGG	YYGGmid	1079	0	0
GYGG	YYWR	1102	0	0
GYGG	YY__	1118	0	0
GYGG	_WYG	1158	0	0
GYGG	Y___	1173	0	0
GYGG	YYGGmid	1192	0	0
GYGG	Y___	1207	0	0
GYGG	_WWW	1229	0	0
GYGG	__BB	1238	0	0
GYGG	__BB	1266	0	0
GYGG	GGGR	1270	0	0
GYGG	_____right	1304	0	0
GYGG	_WWW	1326	0	0
GYGG	_____right	1423	0	0
_____corner	YYRG	772	0	0
_____corner	GYGG	781	0	0
_____corner	GGW_	797	0	0
_____corner	__BB	804	0	0
_____corner	__BB	811	0	0
_____corner	_WWW	821	0	0
_____corner	_Y__	824	0	0
_____corner	__BB	830	0	0
_____corner	GYGG	845	0	0
_____corner	_Y__	851	0	0
_____corner	__BB	856	0	0
_____corner	_Y__	865	0	0
_____corner	_____bm	869	0	0
_____corner	_Y__	872	0	0
_____corner	YGWY	885	0	0
_____corner	GBGW	893	0	0
_____corner	YYRB	896	0	0
_____corner	GRWG	906	0	0
_____corner	_____pale	910	0	0
_____corner	_____topleft	934	0	0
_____corner	YWGW	945	0	0
_____corner	YWGW	1170	0	0

_____corner	GGRR	1366	0	0
_____corner	YWGW	1401	0	0
___BB	YYRG	797	0	0
___BB	_____corner	809	0	0
___BB	YYRG	811	0	0
___BB	_____corner	813	0	0
___BB	GYGG	882	0	0
___BB	GYGG	1002	0	0
___BB	_WWW	1016	0	0
___BB	YYGGright	1101	0	0
___BB	YYGGright	1109	0	0
___BB	_Y__	1125	0	0
___BB	GGWY	1150	0	0
___BB	_WWW	1196	0	0
___BB	GYGG	1269	0	0
___BB	YGWW	1301	0	0
___BB	YGWW	1347	0	0
___BB	GYGG	1352	0	0
_____bm	WGWB	803	0	0
_____bm	WGWB	807	0	0
_____bm	___BB	808	0	0
_____bm	GGW_	844	0	0
_____bm	YYRG	848	0	0
_____bm	_Y__	868	0	0
_____bm	GRBR	873	0	0
_____bm	YYRG	1001	0	0
_____bm	YYRG	1060	0	0
_____bm	GRBR	1095	0	0
_____bm	WRWR	1216	0	0
_____bm	WRWR	1224	0	0
_____bm	RWGY	1232	0	0
_____bm	_R__	1235	0	0
_____bm	YGWW	1237	0	0
_____bm	WG_R	1248	0	0
_____bm	WGGB	1259	0	0
_____bm	WG_R	1266	0	0
_____bm	Q	1270	0	0
_____bm	_WGG	1276	0	0
_____bm	YYGGmid	1279	0	0
_____bm	GGRR	1288	0	0
_____bm	WWBG	1311	0	0
_____bm	GGRR	1320	0	0
_____bm	YWGW	1329	0	0
_____bm	YWGW	1333	0	0
_____bm	_____corner	1350	0	0
_____bm	YWGW	1360	0	0
_____bm	_____corner	1380	0	0
_____bm	YWGW	1410	0	0
_____bm	YWGW	1417	0	0

WGWB	YYRG	786	0	0
WGWB	YYRG	792	0	0
WGWB	__BB	795	0	0
WGWB	__BB	800	0	0
WGWB	____bm	804	0	0
WGWB	YY__	812	0	0
WGWB	GGW_	822	0	0
WGWB	__BB	843	0	0
WGWB	_Y__	845	0	0
WGWB	____corner	846	0	0
WGWB	____corner	850	0	0
WGWB	GRYY	851	0	0
WGWB	_WYW	856	0	0
WGWB	YYGGmid	863	0	0
WGWB	____pale	866	0	0
WGWB	WRBB	869	0	0
WGWB	____pale	876	0	0
WGWB	WGGB	881	0	0
WGWB	WBGW	891	0	0
WGWB	GR_Y2	910	0	0
WGWB	G_R_	997	0	0
WGWB	WGBB	1025	0	0
WGWB	RWWG	1038	0	0
WGWB	WYGG	1048	0	0
WGWB	RWWG	1056	0	0
WGWB	GGYW	1067	0	0
WGWB	WGBB	1197	0	0
WGWB	YYGW	1221	0	0
WGWB	GBG_	1227	0	0
WGWB	YYGW	1236	0	0
WGWB	GGYW	1265	0	0
WGWB	GBG_	1291	0	0
WGWB	GGYW	1294	0	0
WGWB	YYGW	1299	0	0
WGWB	GBG_	1305	0	0
YYRG	GGW_	773	0	0
YYRG	WGWB	789	0	0
YYRG	____corner	798	0	0
YYRG	GGW_	801	0	0
YYRG	_Y__	802	0	0
YYRG	____corner	811	0	0
YYRG	YY_R	838	0	0
YYRG	GRYY	860	0	0
YYRG	_WWW	878	0	0
YYRG	GRBR	923	0	0
YYRG	____bm	1002	0	0
YYRG	GRYY	1114	0	0
YYRG	GRYY	1140	0	0
YYRG	_WWW	1256	0	0

YYRG	GGGR	1335	0	0
YYRG	GGGR	1414	0	0
YYRG	GGGR	1426	0	0
GGW_	YYRG	772	0	0
GGW_	____corner	779	0	0
GGW_	_Y__	782	0	0
GGW_	_Y__	793	0	0
GGW_	____corner	798	0	0
GGW_	_Y__	801	0	0
GGW_	YYRG	803	0	0
GGW_	YYRG	812	0	0
GGW_	_WYG	821	0	0
GGW_	_WYG	837	0	0
GGW_	YY__	842	0	0
GGW_	____bm	845	0	0
GGW_	_Y__	852	0	0
GGW_	GYGG	857	0	0
GGW_	____right	865	0	0
GGW_	GGGR	873	0	0
GGW_	YYGGright	879	0	0
GGW_	____right	882	0	0
GGW_	YYGGright	886	0	0
GGW_	WBGG	927	0	0
GGW_	GGGR	1000	0	0
GGW_	GYGG	1020	0	0
GGW_	GYGG	1037	0	0
GGW_	_WYG	1054	0	0
GGW_	YYWR	1080	0	0
GGW_	GBGW	1102	0	0
GGW_	YYRB	1115	0	0
GGW_	YYY_	1126	0	0
GGW_	YY_W	1156	0	0
GGW_	G___big	1200	0	0
GGW_	YY_W	1222	0	0
GGW_	YY_W	1334	0	0
_Y__	YYRG	785	0	0
_Y__	GGW_	794	0	0
_Y__	GGW_	802	0	0
_Y__	____corner	824	0	0
_Y__	WGWB	846	0	0
_Y__	GGW_	853	0	0
_Y__	GGW_	860	0	0
_Y__	GYGG	1038	0	0
_Y__	GYGG	1053	0	0
_Y__	YYGGright	1100	0	0
_Y__	__BB	1126	0	0
_Y__	YYGG	1157	0	0
_Y__	YGWW	1296	0	0
_Y__	_WYG	1307	0	0

_Y__	YGWW	1374	0	0

Antlist	InitialPosX	InitialPosY	ColonyArea	AntLength	MinX	MinY	MaxX	MaxY
GBGW	169	476	384887	58.8	19	0	721	575
WBYG	226	544						
YGWY	277	526						
GBGR	240	468						
YYRB	177	369						
WRRY	162	320						
YYY_	115	327						
GY__	136	304						
GWRG	168	282						
_R__	100	281						
G___big	56	245						
G___small	85	216						
YYYY	149	233						
YW__	99	190						
GYYY	87	69						
WWBG	54	77						
_RYG	45	60						
GRWG	220	146						
G_R_	182	109						
WRR_	233	92						
YWGW	245	63						
GGRR	270	104						
G_GW	355	38						
Y__W	364	1						
YWW_	228	236						
YYGG	277	363						
Y_WY	346	207						
WRBB	331	210						
WRWR	258	295						
YYGGmid	332	198						
WR__	298	435						
W___	307	488						
YYWR	351	541						
GGRY	356	458						
YY__	421	520						
_WYG	426	561						
GGWY	360	390						
GG_W	445	431						
GGWW	474	417						
GRBR	508	459						
YYRG	609	529						
_Y__	615	541						
GGW_	670	127						
GYGG	642	509						
__BB	578	506						
GRYY	579	414						
YY_R	529	421						
GRGY	470	361						
YWWW	436	307						
_WWW	599	431						
GGGR	625	431						
YYGGright	656	427						
Y___	553	366						
_WWY	479	290						
Q	539	273						
GR_Y2	604	377						
WBGG	675	377						
_W_Y	692	357						
GBG_	651	270						
G___	591	304						
_WYW	616	227						
WGGB	506	243						
_W__	416	219						
GR_Y	444	152						
YGWW	434	115						
GR__	547	77						
RWGY	606	130						
G_W_	590	30						
YY_W	613	38						
_WGG	590	100						
WGBB	601	188						
GGYW	625	174						
RWWG	645	161						
GGGG	670	193						
YYGW	700	150						
WYGG	631	98						
GGW_2	616	543						
WG_R	188	56						
_____topleft	139	88						
_____bot	317	576						
__W_	387	249						
_____pale	513	213						
_____brood	150	151						
WGWB	575	539						
_____bm	549	508						
_____corner	607	495						
_____right	690	435						
_____topright	661	113						
_____almost	652	120						
WBGW	647	30						

Actor	Target	ActorPosX	ActorPosY	Time
_W_R	_WGY	276	66	82
_W_R	WRRY	264	46	268
_W_R	WRRY	264	85	283
_W_R	WRRY	266	88	285
_W_R	WRRY	256	88	289
_W_R	_GWY	251	89	348
_W_R	WRRY	279	126	359
_W_R	_GWY	277	122	637
_W_R	_GWY	278	119	656
_W_R	_WGY	300	109	700
_W_R	_GWY	251	71	722
_W_R	W_WR	286	115	844
_W_R	W_WR	287	108	892
_W_R	G_RG	287	105	937
_W_R	YY__	297	104	945
_W_R	YY_W	288	100	975
_W_R	YY_W	281	103	1039
_W_R	_WWY	284	104	1132
_W_R	YY__	283	106	1153
_W_R	YY__	281	102	1203
_W_R	YY__	270	101	1412
_W_R	____hidden	281	90	1448
_W_R	_GWY	271	124	1458
_W_R	Y___	235	129	1494
_W_R	____hidden	252	103	1496
_W_R	W___	343	43	1592
_W_R	____hidden	314	50	1606
_W_R	_WRW	317	44	1647
_WRW	WRRY	204	132	87
_WRW	WRRY	174	111	145
_WRW	YY__	186	189	269
_WRW	_GWY	185	164	370
_WRW	_GWY	250	59	770
_WRW	_GWY	274	75	1192
_WRW	W___	313	75	1282
_WRW	_W_R	286	79	1285
_WRW	YY__	316	45	1432
_WRW	YY__	299	33	1454
_WRW	_W_R	292	35	1504
_WRW	W___	396	40	1528
_WRW	_RWG	428	40	1540
_WRW	_W__	423	75	1563
_WRW	G_RG	429	55	1619
_WRW	_W_R	347	37	1642
_WRW	____hidden	307	54	1650
_WRW	YY__	176	50	1720
_WRW	____hidden	172	96	1738
Y___	GR_W	195	190	87

Y___	_WRW	193	134	335
Y___	_GWY	186	135	353
Y___	W_WR	328	115	440
Y___	_W_R	283	136	454
Y___	WRRY	260	200	461
Y___	_WRW	194	174	488
Y___	GR_W	234	183	848
Y___	YY__	224	173	1404
Y___	_GWY	220	170	1498
Y___	GR_W	219	176	1621
WRRY	GR_W	240	187	121
WRRY	_WRW	201	105	142
WRRY	Y___	214	127	273
WRRY	_W_R	246	108	285
WRRY	_W_R	239	101	287
WRRY	_W_R	283	150	357
WRRY	Y___	259	202	461
WRRY	GGGG	289	283	503
WRRY	GGGG	287	351	589
WRRY	WGGB	311	442	636
WRRY	GBGR	334	426	660
WRRY	YYRR	289	433	702
WRRY	GGGG	293	356	758
WRRY	GB__	288	352	787
WRRY	GGGG	316	332	855
WRRY	GGGG	298	339	916
WRRY	GB__	283	339	939
WRRY	GG_W	308	334	1655
WRRY	GB__	272	337	1680
WRRY	YYRR	293	328	1687
WRRY	GB__	266	321	1708
GR_W	____hidden	228	236	48
GR_W	Y___	206	218	85
GR_W	WRRY	246	212	121
GR_W	____hidden	254	222	123
GR_W	Y___	251	185	139
GR_W	_R__	199	343	403
GR_W	_R__	191	382	449
GR_W	GYG_	231	378	727
GR_W	YY__	244	347	752
GR_W	GB__	273	317	755
GR_W	____hidden	235	213	827
GR_W	Y___	252	213	842
GR_W	_WWY	224	204	1110
GR_W	Y___	232	199	1344
GR_W	Y___	220	205	1600
GR_W	Y___	221	194	1623
GR_W	YYRB	250	205	1684
GR_W	_WWY	478	135	1719

GR_W	YGWY	511	137	1728
____hidden	GR_W	232	248	49
____hidden	YY__	218	269	241
____hidden	YYG_	234	273	250
____hidden	YY__	219	263	272
____hidden	YYG_	224	266	354
____hidden	GR_W	221	267	389
____hidden	YYG_	213	289	426
____hidden	WRRY	250	210	470
____hidden	Y___	196	204	511
____hidden	Y___	273	148	933
____hidden	_W_R	278	129	945
____hidden	YY__	250	164	963
____hidden	YY__	235	275	1023
____hidden	Y___	261	185	1040
____hidden	_W_R	239	106	1049
____hidden	_WRW	240	88	1164
____hidden	_W_R	249	110	1178
____hidden	_WRW	263	71	1338
____hidden	YY__	279	65	1480
____hidden	_W_R	234	88	1495
____hidden	_WRW	244	41	1504
____hidden	_GWY	253	147	1529
____hidden	YY_W	307	109	1569
____hidden	W_WR	337	114	1578
____hidden	W___	333	84	1600
____hidden	_W_R	318	70	1606
____hidden	YY__	297	75	1623
____hidden	_W_R	314	69	1632
____hidden	_WRW	295	63	1650
____hidden	YY_W	257	76	1660
____hidden	YYRB	268	154	1686
____hidden	Y___	241	147	1702
____hidden	_WRW	192	114	1739
W_WR	YYGG	328	189	50
W_WR	_W__	379	137	143
W_WR	Y___	371	134	410
W_WR	YYGG	374	141	426
W_WR	_W__	373	133	465
W_WR	YY__	369	132	680
W_WR	YY__	345	87	810
W_WR	_W_R	320	119	890
W_WR	YY_W	340	135	939
W_WR	_Y__	362	124	1118
W_WR	_Y__	371	116	1470
W_WR	YY__	330	94	1476
W_WR	YY_W	364	113	1565
W_WR	G_RG	380	111	1594
W_WR	YYRB	349	120	1606

W_WR	YYRB	323	128	1614
W_WR	YYRB	289	130	1631
W_WR	YYRB	254	166	1664
W_WR	YY_W	240	100	1689
W_WR	YY_W	153	126	1698
YYGG	W_WR	328	198	50
YYGG	YYRB	321	200	178
YYGG	WRRY	297	175	358
YYGG	YYRB	325	193	401
YYGG	GG_W	354	171	416
YYGG	GGGG	289	210	437
YYGG	GRBR	300	293	509
YYGG	GGGG	296	328	574
YYGG	GY_W	370	310	868
YYGG	GBGR	358	402	1033
YYGG	YYRR	344	467	1130
YYGG	GGGG	408	441	1244
YYGG	YY__	406	444	1285
YYGG	YY__	415	439	1318
YYGG	_YGG	397	464	1339
YYGG	_YGG	331	455	1427
YYGG	GRY_	325	409	1511
YYGG	WGGB	357	467	1572
YYGG	GRY_	288	410	1702
YYGG	GRY_	311	372	1731
YYGG	GRY_	321	415	1745
YYRB	____white	364	269	50
YYRB	GG_W	380	246	119
YYRB	YYGG	327	234	170
YYRB	GB__	347	229	625
YYRB	GRBR	348	206	655
YYRB	GRBR	365	263	723
YYRB	YY_W	355	199	1121
YYRB	GG_W	351	198	1440
YYRB	Q	366	247	1553
YYRB	GGWW	303	250	1559
YYRB	YY__	272	150	1593
YYRB	W_WR	341	130	1605
YYRB	YY__	287	120	1609
YYRB	Y___	241	171	1630
____white	YYRB	342	279	54
____white	GRBR	387	262	740
____white	GRBR	383	264	954
GB__	WRRY	295	313	509
GB__	GGGG	310	283	532
GB__	YYGG	330	291	554
GB__	YYRB	322	236	625
GB__	GRBR	283	241	645
GB__	GGGG	294	317	755

GB__	WRRY	255	312	940
GB__	YYG_	256	318	1035
GB__	_WWY	271	306	1092
GB__	GYG_	268	309	1218
GB__	WRRY	269	320	1682
GB__	WRRY	271	312	1711
GRBR	YYGG	345	332	527
GRBR	____mid	397	332	554
GRBR	YYGG	326	348	583
GRBR	YYGW	348	363	588
GRBR	YYRB	341	230	654
GRBR	YYRB	366	247	723
GRBR	____white	385	242	738
GRBR	____mid	367	293	950
GRBR	GGGG	363	288	983
GRBR	_WWY	367	286	1089
GRBR	GGW_	371	291	1300
GRBR	_GWY	372	292	1554
YYG_	____hidden	251	301	113
YYG_	GYG_	248	297	306
YYG_	____hidden	223	277	354
YYG_	____hidden	227	271	382
YYG_	GR_W	219	306	390
YYG_	GYG_	242	347	406
YYG_	GR_W	234	343	736
YYG_	_R__	193	360	817
YYG_	GYG_	236	380	940
YYG_	GB__	237	324	1033
YYG_	GR_W	205	313	1475
YYGW	YY__	258	368	180
YYGW	GRBR	304	358	209
YYGW	GRBR	367	382	589
YYGW	GGGG	364	384	651
YYGW	GGGG	365	381	772
YYGW	____mid	376	372	860
YYGW	YYYY	470	394	931
YYGW	GRY_	508	436	950
YYGW	GGRG	517	442	960
YYGW	GGRG	520	440	969
YYGW	____still	576	304	979
YYGW	YGWR	570	288	1010
YYGW	YYRG	553	251	1055
YYGW	GR_Y	526	296	1080
YYGW	YGWR	531	295	1081
YYGW	YGWR	550	321	1085
YYGW	YYRG	487	195	1227
YYGW	YYRG	486	112	1237
YYGW	GR_Y	547	236	1583
YYGW	GY__	528	236	1588

YYGW	YYBR	586	278	1707
YYGW	GGW_	465	312	1717
YYGW	YYBR	459	358	1721
YYGW	GGRG	511	355	1724
_R__	GR_W	178	391	450
GYG_	YYRR	228	386	4
GYG_	YYGW	233	384	9
GYG_	YYGW	215	376	25
GYG_	_R__	195	378	108
GYG_	YY__	227	369	234
GYG_	YYG_	216	317	295
GYG_	YYG_	224	315	306
GYG_	YYG_	239	367	407
GYG_	GR_W	203	376	504
GYG_	YYRR	227	394	525
GYG_	YYG_	246	389	905
GYG_	WRRY	287	391	920
GYG_	YYG_	255	382	940
GYG_	_R__	198	371	1083
GYG_	GB__	251	331	1217
GYG_	YYG_	241	333	1219
GYG_	GB__	252	336	1243
GYG_	WRRY	269	371	1275
GYG_	WGGB	277	417	1316
GYG_	GRY_	278	408	1319
GYG_	WGGB	282	415	1331
YYRR	WGGB	254	440	35
YYRR	WGGB	259	444	98
YYRR	WGGB	267	451	109
YYRR	GYG_	251	364	518
YYRR	GYG_	247	386	525
YYRR	WGGB	302	480	685
YYRR	GYG_	271	427	900
YYRR	_WWY	300	434	958
YYRR	WGGB	313	470	1036
YYRR	GBGR	313	447	1091
YYRR	YYGG	335	466	1130
YYRR	GGWW	333	434	1168
YYRR	GGWW	329	381	1196
YYRR	____still	354	385	1211
YYRR	G__Y	394	351	1476
YYRR	GGWW	364	365	1602
YYRR	WRRY	320	332	1687
WGGB	YYRR	282	452	35
WGGB	YYRR	285	448	108
WGGB	_YGG	315	481	317
WGGB	GBGR	343	473	682
WGGB	_WWY	337	478	941
WGGB	GBGR	346	473	1010

WGGB	YYRR	337	488	1038
WGGB	YYRR	322	480	1044
WGGB	GGWW	257	466	1185
WGGB	YYGG	314	446	1448
WGGB	_YGG	333	472	1506
WGGB	_YGG	319	488	1552
WGGB	YYGG	340	477	1574
YY__	GBGR	347	463	11
YY__	GY_W	442	426	65
YY__	GY_W	433	374	76
YY__	G__Y	544	337	83
YY__	GRY_	543	317	86
YY__	GBGR	373	457	116
YY__	GBGR	364	432	124
YY__	GBGR	362	414	172
YY__	YYGW	293	383	176
YY__	GYG_	233	389	192
YY__	____hidden	213	285	241
YY__	_WRW	209	208	265
YY__	____hidden	268	241	275
YY__	WRRY	264	138	281
YY__	W_WR	324	127	285
YY__	_GWY	318	99	287
YY__	_GWY	303	87	317
YY__	_GWY	285	94	324
YY__	G___	429	58	347
YY__	_WYG	437	73	357
YY__	G___	422	58	360
YY__	YWWW	360	61	364
YY__	YWWW	322	72	384
YY__	_GWY	246	117	398
YY__	WRRY	275	135	414
YY__	_W_R	311	115	463
YY__	_GWY	254	95	493
YY__	_GWY	265	97	541
YY__	W_WR	342	99	591
YY__	_WGY	370	65	620
YY__	W_WR	367	109	680
YY__	_WGY	360	94	697
YY__	_GWY	324	69	710
YY__	_GWY	320	73	724
YY__	W_WR	347	72	821
YY__	_GWY	323	77	832
YY__	_W_R	298	90	845
YY__	_GWY	288	68	862
YY__	YWWW	333	101	941
YY__	_W_R	306	121	950
YY__	____hidden	260	184	963
YY__	GB__	270	291	990

YY__	YY_W	275	250	1025
YY__	YY_W	282	156	1037
YY__	W_WR	306	130	1044
YY__	_GWY	313	92	1048
YY__	YY_W	299	133	1117
YY__	W_WR	327	91	1129
YY__	W_WR	333	119	1138
YY__	W_WR	337	122	1145
YY__	_WWY	270	134	1150
YY__	Y___	250	155	1156
YY__	_WWY	259	159	1160
YY__	GY_W	256	162	1164
YY__	_W_R	262	131	1199
YY__	_GWY	249	153	1230
YY__	GR_W	248	154	1400
YY__	_GWY	258	151	1405
YY__	_W_R	261	89	1413
YY__	GY_W	283	88	1425
YY__	_WRW	313	70	1432
YY__	W_WR	348	62	1444
YY__	YY_W	306	111	1470
YY__	W_WR	312	97	1475
YY__	GY_W	308	76	1478
YY__	_WRW	293	66	1482
YY__	_W_R	273	77	1511
YY__	GY_W	223	120	1562
YY__	Y___	231	154	1586
YY__	YYBR	265	125	1594
YY__	YYBR	266	99	1609
YY__	YY_W	247	88	1614
YY__	_W_R	277	47	1633
YY__	W_WR	351	86	1649
YY__	_Y__	396	145	1673
YY__	G_RG	359	73	1682
YY__	_WRW	209	66	1720
GBGR	YY__	354	437	12
GBGR	YY__	366	453	115
GBGR	YY__	347	421	124
GBGR	YY__	353	430	134
GBGR	_YGG	369	463	328
GBGR	_YGG	373	468	408
GBGR	WRRY	357	444	675
GBGR	WGGB	358	455	682
GBGR	_WWY	368	453	939
GBGR	GRGY	361	449	1048
GBGR	GRGY	349	442	1062
GBGR	GGGG	335	384	1081
GBGR	YYRR	322	416	1092
GBGR	____still	385	395	1123

GBGR	GGGG	376	378	1128
GBGR	YGWR	571	322	1145
GBGR	GR_Y	591	277	1156
GBGR	____new	597	256	1167
GBGR	G___right	631	248	1185
GBGR	YGWR	590	282	1201
GBGR	GBG_	564	310	1241
GBGR	YGWR	566	294	1253
GBGR	YYYY	526	405	1275
GBGR	_GYW	527	379	1293
GBGR	YYYY	536	408	1304
GBGR	WR__	545	424	1323
GBGR	GRY_	636	344	1350
GBGR	GBG_	628	336	1355
GBGR	GRY_	637	337	1357
GBGR	WR__	566	431	1387
GBGR	_GYW	568	379	1395
GBGR	_GYW	560	365	1410
GBGR	GBG_	591	352	1550
GBGR	_GYW	571	369	1555
GBGR	YYYY	511	325	1567
GBGR	GY__	496	244	1584
GBGR	YYYY	513	280	1598
GBGR	____mid	513	373	1654
GBGR	YYRR	369	378	1671
GBGR	YYRR	341	360	1702
GBGR	GGGG	290	261	1736
Y_B_	YYGW	405	368	919
Y_B_	YYGG	383	366	931
Y_B_	____mid	431	361	938
Y_B_	_WGY	468	374	947
Y_B_	YYYY	514	380	997
Y_B_	____mid	537	379	1020
Y_B_	____mid	598	352	1056
Y_B_	WR__	553	449	1124
Y_B_	_W_W	578	469	1201
Y_B_	_W_W	580	438	1295
Y_B_	_W_W	576	458	1327
Y_B_	GGGG	503	408	1663
Y_B_	_GWY	499	419	1730
____mid	GY_W	395	332	366
____mid	YYGG	398	335	632
____mid	YYGG	397	328	802
____mid	GGW_	404	312	930
____mid	GGW_	421	318	937
____mid	GRBR	382	313	947
____mid	GGW_	413	309	954
____mid	G__Y	454	335	962
____mid	YYYY	542	396	981

_____mid	YYYY	545	418	989
_____mid	_W_W	593	438	1037
_____mid	GGWW	614	373	1054
_____mid	GGRG	633	351	1058
_____mid	YGWR	619	296	1063
_____mid	YYGW	580	346	1085
_____mid	G__Y	558	377	1090
_____mid	G__Y	541	396	1094
_____mid	GRGY	548	393	1144
_____mid	GGRG	595	401	1154
_____mid	GRGY	564	402	1158
_____mid	YYYY	489	401	1178
_____mid	G__Y	446	342	1207
_____mid	GGW_	430	331	1215
_____mid	GY_W	496	323	1227
_____mid	GGW_	431	310	1302
_____mid	G__Y	422	350	1346
_____mid	G__Y	419	349	1707
GY_W	YY__	453	378	74
GY_W	YY__	473	374	79
GY_W	YY__	414	421	123
GY_W	_WWY	418	412	976
GY_W	Y_B_	446	384	995
GY_W	_____mid	497	397	1033
GY_W	_____mid	553	416	1041
GY_W	YYBR	602	466	1053
GY_W	WR__	529	452	1098
GY_W	YY__	268	187	1162
GY_W	YGWY	560	129	1197
GY_W	GBGR	601	252	1205
GY_W	GGW_	489	265	1222
GY_W	_____mid	500	303	1228
GY_W	GRGY	326	402	1315
GY_W	G__Y	400	390	1344
GY_W	_GWY	413	397	1584
GY_W	GBGR	477	364	1654
GGW_	G__Y	446	305	636
GGW_	_____mid	437	304	881
GGW_	_____mid	441	302	931
GGW_	_____mid	449	315	1311
GGW_	_____white	441	310	1569
GYY_	GGGR	460	215	89
GYY_	GGGR	459	220	113
GYY_	Q	460	214	785
GYY_	GGGR	467	241	827
GYY_	Q	463	220	1345
GYY_	GGGR	458	223	1388
GYY_	Q	464	237	1432
GYY_	GGGR	483	213	1565

GYY_	YYRG	482	228	1573
GYY_	GGGR	479	214	1593
GGGR	GYY_	439	221	102
GGGR	GYY_	443	226	810
GGGR	Q	423	233	1537
GGGR	YYGW	494	275	1635
GGGR	GGW_	483	290	1640
GGGR	_GWY	488	308	0
GGGR	GRGY	354	399	1705
GGGR	____mid	387	344	1719
GGGR	GGW_	438	334	1733
Q	GYY_	461	191	695
Q	_Y__	462	197	787
Q	GYY_	465	202	794
Q	GGW_	470	198	796
Q	GGGR	447	201	1377
Q	GYY_	449	200	1407
Q	_Y__	411	180	1489
Q	GGGR	404	213	1538
Q	____white	409	253	1551
Q	YYRB	384	235	1555
Q	GG_W	368	167	1570
Q	GGGR	457	202	1616
Q	GYY_	452	187	1637
Q	GYY_	486	157	1658
Q	_WWY	519	117	1666
Q	YGWY	610	149	1679
Q	_GWY	608	151	1681
Q	YGWY	612	179	1686
Q	_GWY	669	159	1693
Q	GR_Y	610	245	1707
Q	YYGW	605	258	1709
Q	GBG_	585	284	1712
Q	GGRG	516	301	1723
GG_W	Q	393	189	1430
GG_W	Q	388	187	1511
GG_W	Q	403	178	1578
GG_W	_Y__	389	159	1594
GG_W	GRBR	340	234	1629
GG_W	WRRY	329	303	1654
GG_W	WRRY	336	301	1666
GG_W	GRBR	344	285	1669
YYRG	GR_Y	487	273	695
YYRG	GGW_	485	271	762
YYRG	GGW_right	488	265	797
YYRG	GR_Y	520	281	838
YYRG	____new	545	222	875
YYRG	____new	556	211	890
YYRG	_W__	519	188	1015

YYRG	_W__	522	189	1022
YYRG	YYGW	506	198	1203
YYRG	YYGW	518	186	1227
YYRG	GR_Y	506	147	1237
YYRG	_Y__	526	196	1308
YYRG	GYY_	513	207	1550
YYRG	WRR_	518	203	1579
YYRG	GGW_right	515	202	1581
YYRG	GYY_	510	209	1625
GR_Y	GRGY	535	295	350
GR_Y	G__Y	495	309	654
GR_Y	G__Y	499	316	690
GR_Y	YYRG	494	288	695
GR_Y	GGW_right	506	292	706
GR_Y	GGW_right	518	284	716
GR_Y	YYRG	497	301	723
GR_Y	GRGY	527	312	765
GR_Y	GRGY	541	292	789
GR_Y	GGW_right	550	274	799
GR_Y	____still	566	278	809
GR_Y	GBG_	563	282	814
GR_Y	YYRG	539	296	838
GR_Y	____still	528	326	980
GR_Y	Y_B_	529	363	987
GR_Y	YYYY	538	405	990
GR_Y	YYGW	539	336	996
GR_Y	_WGY	519	322	1006
GR_Y	YYGW	532	298	1012
GR_Y	____new	524	294	1014
GR_Y	____new	551	246	1136
GR_Y	GBGR	580	241	1158
GR_Y	GGW_right	539	141	1209
GR_Y	_WYG	480	80	1246
GR_Y	_W__	463	84	1251
GR_Y	_Y__	434	105	1264
GR_Y	WRR_	453	102	1268
GR_Y	YYRG	543	160	1285
GR_Y	____new	592	239	1296
GR_Y	YYGW	592	258	1299
GR_Y	YGWR	592	263	1301
GR_Y	____new	577	230	1336
GR_Y	YGWY	561	231	1580
GR_Y	WRR_	567	230	1581
GR_Y	YGWY	568	242	1703
_Y__	__W_	492	208	102
_Y__	Q	485	210	781
_Y__	GGW_right	485	203	785
_Y__	GGW_right	490	191	823
_Y__	GY__	590	179	833

_Y__	____new	587	199	834
_Y__	YGWY	582	159	847
_Y__	GY__	610	201	884
_Y__	YYRG	536	157	898
_Y__	YY_W	500	140	903
_Y__	YY_W	460	128	924
_Y__	W_WR	407	133	1100
_Y__	_WWY	410	131	1245
_Y__	GR_Y	418	120	1266
_Y__	WRR_	443	119	1278
_Y__	_WWY	435	131	1282
_Y__	_WGY	457	136	1292
_Y__	YYRG	502	175	1308
_Y__	W_WR	402	134	1466
_Y__	W_WR	400	130	1516
_Y__	G_RG	399	123	1540
_Y__	W_WR	388	138	1588
__W_	_Y__	485	162	88
__W_	_WGY	490	164	337
__W_	_Y__	498	167	786
__W_	GGW_right	503	179	791
__W_	W___	468	100	819
__W_	GGW_right	505	93	828
__W_	YY_W	474	121	835
__W_	_WWY	441	127	844
__W_	YY_W	443	137	873
__W_	WBYG	582	63	896
__W_	YWWW	713	55	915
__W_	WBYG	617	53	1005
__W_	_WGY	632	140	1213
__W_	YGWY	625	128	1219
__W_	WBYG	657	94	1489
__W_	WRR_	539	107	1737
YY_W	_WWY	445	136	45
YY_W	_WGY	450	140	53
YY_W	_W__	459	132	57
YY_W	_W__	505	112	77
YY_W	YWWW	460	69	292
YY_W	WBYG	458	117	327
YY_W	G___	436	92	343
YY_W	YY__	435	117	357
YY_W	_WYG	428	123	469
YY_W	YWWW	428	118	525
YY_W	_WGY	437	123	613
YY_W	_WGY	434	122	744
YY_W	W___	433	120	792
YY_W	W___	431	123	812
YY_W	GGW_right	504	93	831
YY_W	W___	470	105	849

YY_W	_WWY	457	128	857
YY_W	G_RG	351	100	933
YY_W	W_WR	344	110	938
YY_W	G_RG	366	90	948
YY_W	_GWY	309	79	956
YY_W	____hidden	267	169	982
YY_W	Y___	248	186	1004
YY_W	YY__	254	247	1016
YY_W	_W_R	292	130	1037
YY_W	YYRB	351	161	1122
YY_W	_WWY	278	183	1152
YY_W	GY_W	285	174	1165
YY_W	_WWY	290	133	1205
YY_W	_GWY	311	134	1213
YY_W	W_WR	327	139	1464
YY_W	_W_R	297	136	1476
YY_W	GGWW	312	141	1505
YY_W	W_WR	347	153	1529
YY_W	W_WR	345	144	1567
YY_W	____hidden	320	136	1573
YY_W	_WRW	211	67	1684
YY_W	_WRW	216	61	1702
YY_W	_WRW	199	60	1742
_WWY	YY_W	425	125	27
_WWY	YY_W	422	129	59
_WWY	G_RG	423	130	60
_WWY	YY_W	428	136	131
_WWY	Q	429	139	815
_WWY	W___	419	131	833
_WWY	__W_	420	120	845
_WWY	W_WR	345	144	885
_WWY	YYGG	356	355	897
_WWY	Y_B_	394	366	899
_WWY	YYRR	317	455	905
_WWY	WGGB	352	481	945
_WWY	YYRR	332	423	958
_WWY	YYGG	377	374	984
_WWY	GGGG	340	340	1055
_WWY	GRBR	338	287	1086
_WWY	GR_W	259	205	1111
_WWY	YY__	270	157	1118
_WWY	YY_W	320	131	1124
_WWY	_W_R	290	119	1130
_WWY	YY__	288	113	1142
_WWY	____hidden	257	133	1149
_WWY	YY_W	281	151	1152
_WWY	GY_W	350	110	1168
_WWY	G_RG	364	75	1181
_WWY	W___	390	27	1194

_WWY	_GWY	343	104	1203
_WWY	W___	350	99	1204
_WWY	YY_W	311	129	1206
_WWY	YY__	270	151	1210
_WWY	GGWW	279	173	1213
_WWY	_Y__	393	149	1240
_WWY	GG_W	396	154	1277
_WWY	_Y__	418	123	1282
_WWY	____hidden	426	116	1286
_WWY	G_RG	429	121	1290
_WWY	WRR_	452	115	1297
_WWY	_WYG	503	118	1476
_WWY	YGWY	526	145	1480
_WWY	_W__	525	142	1482
_WWY	WRR_	494	126	1496
_WWY	_WYG	484	108	1519
_WWY	WRR_	449	115	1528
_WWY	_WYG	459	95	1552
_WWY	WRR_	508	116	1564
_WWY	_WYG	527	119	1622
_WWY	WRR_	532	102	1626
_WWY	_WYG	536	81	1655
_WWY	_WYG	522	98	1664
_WWY	_WYG	502	77	1677
_WWY	WRR_	508	129	1708
_WYG	YY_W	486	136	13
_WYG	_WGY	483	118	25
_WYG	YY_W	494	131	42
_WYG	_W__	496	84	49
_WYG	_WGY	489	83	52
_WYG	_W__	497	84	55
_WYG	_WGY	510	83	61
_WYG	WB__	513	60	65
_WYG	_WGY	508	66	67
_WYG	_W__	498	79	80
_WYG	_WGY	493	109	104
_WYG	_W__	497	81	123
_WYG	_W__	497	75	137
_WYG	G_RG	508	59	167
_WYG	G_RG	503	75	301
_WYG	WB__	487	60	305
_WYG	G_RG	499	60	320
_WYG	WRR_	468	57	341
_WYG	YY_W	476	70	348
_WYG	_W__	471	99	354
_WYG	G___	471	99	360
_WYG	_W__	454	77	384
_WYG	YWWW	405	52	406
_WYG	YWWW	417	48	444

_WYG	_W__	467	82	492
_WYG	YWWW	464	86	495
_WYG	WB__	503	57	524
_WYG	_W__	500	57	534
_WYG	YWWW	492	85	672
_WYG	G_RG	501	69	744
_WYG	_W__	489	60	758
_WYG	WB__	494	51	769
_WYG	_W__	479	58	791
_WYG	W___	464	75	842
_WYG	_WGY	509	68	880
_WYG	GGW_right	598	57	916
_WYG	WB__	483	52	980
_WYG	GGW_right	492	56	1025
_WYG	_W__	486	57	1165
_WYG	_W__	495	55	1187
_WYG	GY_W	491	49	1238
_WYG	GGW_right	485	43	1250
_WYG	WRR_	472	34	1259
_WYG	Y_BW	481	36	1275
_WYG	Y_BW	485	54	1292
_WYG	GGW_right	518	56	1430
_WYG	_W__	493	87	1437
_WYG	WRR_	493	106	1480
_WYG	_WWY	484	110	1530
_WYG	WRR_	475	117	1537
_WYG	_W__	478	96	1572
_WYG	G_RG	476	104	1577
_WYG	_W__	475	95	1588
_WYG	WRR_	492	111	1613
_WYG	_WWY	513	109	1622
_WYG	WRR_	518	92	1624
_WYG	_WWY	520	87	1655
_WYG	_WWY	483	75	1677
_WYG	Y_BW	467	54	1698
_WYG	WRR_	488	75	1707
_WYG	Y_BW	488	59	1726
_WYG	G_RG	467	102	1744
_W__	_WYG	478	67	48
_W__	_RWG	463	62	52
_W__	_WYG	474	59	54
_W__	WB__	476	54	60
_W__	_RWG	468	51	61
_W__	G_RG	463	58	64
_W__	G___	467	46	68
_W__	_RWG	450	70	72
_W__	G___	451	56	74
_W__	WB__	581	51	103
_W__	_WYG	473	50	108

_W__	WB__	456	43	136
_W__	G___	455	44	226
_W__	_RWG	460	42	245
_W__	_GWY	457	39	251
_W__	WB__	474	48	260
_W__	_GWY	471	46	265
_W__	_RWG	465	38	269
_W__	G_RG	496	51	299
_W__	YWWW	463	65	302
_W__	YWWW	446	69	319
_W__	YWWW	432	54	322
_W__	G___	407	53	325
_W__	YWWW	444	50	330
_W__	WB__	484	62	336
_W__	WB__	484	49	347
_W__	_RWG	483	53	349
_W__	_RWG	473	41	361
_W__	_WYG	471	63	381
_W__	_RWG	470	52	397
_W__	WB__	481	53	427
_W__	_RWG	456	46	461
_W__	WB__	468	30	534
_W__	_WYG	483	54	559
_W__	WB__	473	37	561
_W__	_WGY	469	39	659
_W__	YWWW	469	38	662
_W__	WB__	473	36	668
_W__	_WGY	316	58	683
_W__	_GWY	305	79	685
_W__	_WGY	329	55	692
_W__	WB__	462	47	699
_W__	WB__	459	41	705
_W__	_RWG	454	38	712
_W__	WB__	456	39	845
_W__	_WYG	460	37	862
_W__	_WYG	496	56	876
_W__	_WYG	494	58	883
_W__	W___	463	32	945
_W__	YY__	472	109	971
_W__	GGW_right	502	123	973
_W__	GGW_right	526	125	1064
_W__	_WYG	485	97	1109
_W__	W___	461	82	1138
_W__	W___	461	50	1150
_W__	_WYG	464	56	1164
_W__	_WYG	461	58	1234
_W__	G_RG	437	48	1246
_W__	_WYG	461	39	1250
_W__	WRR_	459	47	1258

_W__	G_RG	473	63	1269
_W__	WRR_	459	70	1275
_W__	_WYG	458	85	1534
_W__	WRR_	458	80	1541
_W__	_WGY	461	92	1572
_W__	G_RG	437	96	1579
_W__	_WYG	459	94	1588
_W__	WRR_	472	81	1652
_W__	WRR_	435	69	1664
_W__	_WYG	445	63	1715
_W__	_RWG	446	50	1729
_W__	G_RG	455	89	1735
_W__	_WYG	457	73	1744
G___	_RWG	413	35	50
G___	_RWG	423	43	62
G___	_W__	440	44	67
G___	_RWG	412	45	86
G___	_W__	416	45	135
G___	_W__	421	48	227
G___	_GWY	408	52	250
G___	_GWY	425	48	258
G___	_GWY	369	36	272
G___	YWWW	414	40	287
G___	YWWW	422	43	310
G___	_W__	383	46	325
G___	YWWW	388	49	335
G___	WRR_	421	45	338
G___	_RWG	453	44	346
G___	YY__	441	62	349
G___	WB__	459	45	358
G___	YY__	440	55	360
G___	_WYG	456	50	368
G___	_RWG	464	25	373
WRR_	_RWG	452	6	336
WRR_	G___	431	50	339
WRR_	G_RG	444	44	1260
WRR_	_W__	439	65	1265
WRR_	GR_Y	464	90	1267
WRR_	GY_W	475	116	1277
WRR_	_Y__	487	117	1296
WRR_	GGRG	479	125	1346
WRR_	_WWY	479	122	1464
WRR_	_WYG	477	129	1485
WRR_	_WWY	472	130	1497
WRR_	_WWY	478	121	1518
WRR_	GGRG	449	122	1526
WRR_	GGRG	441	125	1533
WRR_	_WYG	452	130	1546
WRR_	_WWY	471	145	1549

WRR_	YGWY	558	150	1569
WRR_	GGRG	530	168	1576
WRR_	YYRG	536	179	1579
WRR_	GR_Y	546	188	1581
WRR_	_WWY	556	113	1586
WRR_	_WYG	528	102	1588
WRR_	_WWY	543	95	1599
WRR_	_WYG	511	110	1612
WRR_	Y_BW	514	61	1621
WRR_	GGW_right	529	51	1628
WRR_	Y_BW	532	49	1634
WRR_	_RWG	437	42	1662
WRR_	WB__	435	35	1683
WRR_	WB__	444	42	1696
WRR_	Y_BW	468	48	1699
WRR_	_WWY	514	115	1707
WRR_	_WWY	530	125	1709
WRR_	YGWY	522	124	1727
WRR_	GY_W	512	127	1747
YWWW	G___	432	47	285
YWWW	_W__	449	39	301
YWWW	WB__	451	41	306
YWWW	_W__	453	37	313
YWWW	G___	446	42	315
YWWW	_W__	452	40	322
YWWW	_W__	452	31	331
YWWW	G___	403	45	334
YWWW	YY__	337	53	365
YWWW	YY__	315	51	386
YWWW	_WYG	370	44	405
YWWW	Y___	351	68	427
YWWW	_WYG	381	46	430
YWWW	_WYG	392	51	463
YWWW	_WYG	447	84	495
YWWW	YY_W	436	82	520
YWWW	_GWY	297	56	592
YWWW	_GWY	271	51	620
YWWW	YY__	324	65	623
YWWW	_WGY	411	72	654
YWWW	_W__	455	80	660
YWWW	_WYG	478	64	672
YWWW	WBYG	531	49	678
YWWW	WBYG	558	45	685
YWWW	W___	669	51	695
YWWW	__W_	716	53	920
YWWW	_WGY	710	59	1184
YWWW	WBYG	677	54	1528
YWWW	GG__	735	112	1647
_RWG	_GWY	458	6	249

_RWG	YWWW	457	14	278
_RWG	WRR_	459	23	337
_RWG	WRR_	461	1	347
_RWG	G___	462	3	373
_RWG	WB__	469	18	422
_RWG	WB__	463	12	467
_RWG	_W__	452	15	528
_RWG	WRR_	450	6	1258
_RWG	WB__	450	8	1459
_RWG	WB__	451	1	1553
_RWG	_W__	455	58	1569
_RWG	WRR_	454	10	1663
_RWG	WB__	455	18	1680
_RWG	Y_BW	472	18	1703
_RWG	WB__	460	22	1710
_GWY	_RWG	444	19	248
_GWY	G___	438	46	250
_GWY	_W__	448	40	253
_GWY	G___	455	47	256
_GWY	G___	448	41	266
_GWY	G___	410	37	269
_GWY	YY__	327	76	287
_GWY	YY__	313	67	302
_GWY	YY__	313	73	317
_GWY	_W_R	278	56	321
_GWY	YY__	288	70	322
_GWY	WRRY	230	56	327
_GWY	_W_R	231	91	348
_GWY	Y___	215	130	352
_GWY	WRRY	226	143	354
_GWY	GR_W	230	170	361
_GWY	_WRW	213	154	369
_GWY	YY__	242	127	412
_GWY	YY__	244	101	542
_GWY	YWWW	268	68	592
_GWY	YY__	310	76	599
_GWY	_W_R	297	102	636
_GWY	_WGY	287	83	696
_GWY	YY__	302	60	708
_GWY	_W_R	285	53	721
_GWY	YY__	308	61	725
_GWY	YY__	303	70	833
_GWY	G_RG	304	63	914
_GWY	YY__	296	72	943
_GWY	_W_R	286	79	968
_GWY	_WWY	316	90	1198
_GWY	YY_W	296	119	1213
_GWY	YY__	266	165	1219
_GWY	_WWY	280	173	1221

_GWY	GGWW	275	158	1233
_GWY	Y___	231	146	1498
_GWY	YY__	248	110	1513
_GWY	____hidden	265	161	1529
_GWY	GGWW	267	205	1541
_GWY	GB__	282	286	1545
_GWY	WRRY	337	342	1559
_GWY	YYGG	343	401	1567
_GWY	G__Y	437	401	1580
_GWY	YYYY	450	417	1583
_GWY	YYYY	459	406	1608
_GWY	WR__	531	438	1623
_GWY	_GYW	572	375	1629
_GWY	GGGR	498	323	1644
_GWY	GBG_	599	277	1670
_GWY	GY__	641	228	1673
_GWY	YGWY	636	210	1674
_GWY	Q	629	152	1681
_GWY	G___right	696	202	1688
_GWY	GG__	715	160	1693
_GWY	GY__	676	237	1697
_GWY	YYGW	596	278	1702
_GWY	Y_B_	551	400	1705
_GWY	WR__	553	410	1706
_GWY	YYYY	525	439	1724
_GWY	_YGG	457	467	1735
_GWY	_YGG	473	479	1746
Y_BW	WB__	468	29	1275
Y_BW	_W__	469	44	1287
Y_BW	GGW_right	497	34	1434
Y_BW	WB__	487	32	1455
Y_BW	WB__	477	40	1473
Y_BW	GGW_right	503	53	1506
Y_BW	GGW_right	499	47	1529
Y_BW	GGW_right	514	45	1568
Y_BW	WRR_	516	36	1631
Y_BW	_WYG	510	45	1634
Y_BW	_W__	508	60	1682
Y_BW	_W__	501	46	1728
Y_BW	YY__	500	49	1737
WB__	_W__	502	48	58
WB__	_RWG	489	37	64
WB__	_W__	484	32	109
WB__	_W__	486	34	253
WB__	_W__	513	36	292
WB__	_RWG	493	31	302
WB__	YWWW	471	34	307
WB__	WBYG	551	33	315
WB__	_RWG	487	35	319

WB__	YWWW	469	30	325
WB__	_RWG	481	34	341
WB__	WRR_	462	30	344
WB__	G___	466	31	355
WB__	G___	478	34	371
WB__	_RWG	481	34	465
WB__	_W__	479	33	530
WB__	_W__	478	32	623
WB__	_W__	480	34	669
WB__	_W__	482	37	705
WB__	_WGY	483	32	712
WB__	_W__	479	33	853
WB__	W___	478	30	958
WB__	WRR_	472	33	1259
WB__	Y_BW	481	36	1266
WB__	Y_BW	493	37	1293
WB__	GGW_right	516	39	1302
WB__	Y_BW	491	30	1341
WB__	WBYG	567	31	1355
WB__	YWWW	650	37	1370
WB__	WBYG	608	41	1376
WB__	Y_BW	478	26	1401
WB__	Y_BW	495	37	1474
WB__	GGW_right	496	37	1496
WB__	_W__	488	69	1503
WB__	WRR_	474	91	1508
WB__	_GWY	478	86	1522
WB__	GGW_right	530	52	1605
WB__	_WYG	512	42	1631
WB__	WRR_	513	44	1634
WB__	_WYG	516	45	1650
WB__	_WWY	504	59	1682
WB__	WRR_	500	46	1704
WB__	_WYG	502	42	1726
WBYG	WB__	571	36	296
WBYG	YWWW	568	35	680
WBYG	W___	585	37	767
WBYG	GGW_right	579	37	1253
WBYG	WB__	585	40	1353
WBYG	WB__	600	35	1376
WBYG	____mid	617	33	1448
WBYG	YWWW	662	55	1476
WBYG	GY_W	648	75	1495
WBYG	YWWW	653	54	1538
WBYG	_WGY	649	8	1570
W___	GGW_right	669	41	582
W___	YWWW	670	36	693
W___	WBYG	608	45	726
W___	_W__	537	78	768

W___	YY_W	463	106	793
W___	_W__	456	93	797
W___	YY_W	441	85	821
W___	_WWY	452	106	831
W___	__W_	454	99	844
W___	_W__	431	64	861
W___	_W__	434	36	944
W___	_RWG	441	35	948
W___	WB__	447	33	956
W___	_WYG	449	52	1069
W___	WB__	452	43	1076
W___	_RWG	451	41	1135
W___	_W__	445	44	1140
W___	_W__	444	46	1151
W___	_WWY	392	88	1197
W___	G_RG	351	77	1213
W___	G_RG	328	54	1229
W___	_WRW	372	59	1519
W___	_W_R	375	63	1591
W___	G_RG	367	70	1646
W___	WRR_	374	63	1690
GGW_right	G_RG	628	84	112
GGW_right	_WGY	629	88	516
GGW_right	WBYG	605	45	548
GGW_right	WBYG	607	58	564
GGW_right	_WGY	636	66	577
GGW_right	W___	636	53	582
GGW_right	W___	649	45	590
GGW_right	_R__right	690	178	633
GGW_right	YGWY	612	160	637
GGW_right	YGWY	590	178	644
GGW_right	____new	574	226	659
GGW_right	GR_Y	510	263	705
GGW_right	____still	562	266	730
GGW_right	GRGY	539	290	734
GGW_right	YYRG	512	250	744
GGW_right	GR_Y	536	246	799
GGW_right	____still	555	241	809
GGW_right	_Y__	507	170	823
GGW_right	__W_	530	105	828
GGW_right	WB__	515	61	847
GGW_right	_WWY	522	75	885
GGW_right	__W_	535	87	903
GGW_right	_WYG	520	89	914
GGW_right	YY__	495	113	920
GGW_right	_WYG	520	95	977
GGW_right	_WYG	513	82	1016
GGW_right	WBYG	544	50	1246
GGW_right	WBYG	564	40	1253

GGW_right	WB__	534	56	1299
GGW_right	WBYG	555	47	1338
GGW_right	WB__	538	49	1342
GGW_right	WB__	528	53	1349
GGW_right	_WYG	501	33	1365
GGW_right	_WYG	524	59	1376
GGW_right	Y_BW	521	41	1437
GGW_right	WB__	527	46	1497
GGW_right	Y_BW	527	52	1511
GGW_right	Y_BW	524	40	1531
GGW_right	Y_BW	539	40	1549
GGW_right	WRR_	544	52	1622
GGW_right	GY_W	640	81	1693
G_RG	_WGY	599	179	30
G_RG	_W__	453	105	57
G_RG	_RWG	445	87	65
G_RG	YY_W	502	84	78
G_RG	WBYG	594	52	104
G_RG	_R__right	709	173	118
G_RG	GY__	693	168	120
G_RG	WBYG	604	54	155
G_RG	_W__	509	70	300
G_RG	WB__	496	50	319
G_RG	_WGY	467	62	380
G_RG	_WYG	471	76	677
G_RG	_WGY	469	72	730
G_RG	_W__	478	64	742
G_RG	_WGY	448	52	766
G_RG	W___	431	52	819
G_RG	_WYG	449	80	868
G_RG	WB__	484	55	887
G_RG	_GWY	332	76	911
G_RG	W_WR	344	84	918
G_RG	YY_W	347	92	932
G_RG	_W_R	322	92	935
G_RG	YY_W	357	73	940
G_RG	_WWY	377	45	1189
G_RG	_W__	440	66	1255
G_RG	WRR_	433	78	1279
G_RG	_W__	448	86	1475
G_RG	_WRW	434	61	1535
G_RG	WRR_	435	97	1546
G_RG	_WYG	429	113	1577
G_RG	_W__	433	113	1591
G_RG	_WRW	429	75	1622
G_RG	_WRW	423	74	1629
G_RG	W___	393	69	1648
G_RG	_W__	440	110	1736
G_RG	_WYG	470	104	1743

YGWY	_WGY	610	132	505
YGWY	_WGY	620	126	583
YGWY	_WGY	607	129	777
YGWY	_Y__	619	140	873
YGWY	GY_W	596	149	1219
YGWY	____new	635	157	1311
YGWY	GY__	657	165	1323
YGWY	YGWR	622	193	1354
YGWY	WRR_	563	168	1571
YGWY	Q	605	177	1679
YGWY	GGW_right	586	202	1690
YGWY	____new	568	172	1711
YGWY	WRR_	540	135	1728
_R__right	G_RG	735	193	119
_R__right	_WGY	731	193	133
_R__right	GG__	729	194	169
_R__right	_GWY	719	200	1690
_R__right	G___right	725	221	1704
GY__	G_RG	682	183	121
GY__	_WGY	676	180	396
GY__	GGWW	692	250	618
GY__	YGWY	605	184	725
GY__	_Y__	609	180	834
GY__	YGWY	657	172	877
GY__	_WGY	670	178	1139
GY__	GY_W	665	172	1231
GY__	YGWY	667	178	1308
GY__	_GWY	682	185	1684
GY__	G___right	676	239	1695
GY__	_GWY	660	249	1698
GY__	_W__curl	647	285	1702
GY__	GBG_	626	321	1705
GY__	_GYW	624	314	1709
GY__	YGWR	672	307	1714
_WGY	GY__	672	192	1
_WGY	_WYG	519	137	8
_WGY	G_RG	574	155	31
_WGY	YGWY	579	161	42
_WGY	____new	602	211	46
_WGY	G___right	525	177	50
_WGY	_WYG	474	100	56
_WGY	_W__	497	76	64
_WGY	YY_W	469	115	69
_WGY	_RWG	411	90	74
_WGY	_W_R	292	70	81
_WGY	_W__	458	99	103
_WGY	_WYG	472	88	105
_WGY	GY__	689	213	130
_WGY	_R__right	725	170	134

_WGY	YGWY	632	155	145
_WGY	YY_W	499	137	171
_WGY	YGWY	581	180	180
_WGY	____new	590	225	204
_WGY	YGWY	580	169	237
_WGY	YY_W	494	134	271
_WGY	G_RG	529	112	279
_WGY	_WYG	521	118	308
_WGY	YY_W	503	128	326
_WGY	_WYG	510	131	341
_WGY	WB__	521	59	350
_WGY	YGWY	560	154	363
_WGY	GG__	704	172	387
_WGY	GY__	670	165	393
_WGY	____new	610	206	407
_WGY	____new	610	226	412
_WGY	YGWY	589	180	476
_WGY	GGW_right	647	116	514
_WGY	W___	665	71	570
_WGY	YGWY	630	180	598
_WGY	YY_W	482	123	612
_WGY	YWWW	420	48	658
_WGY	_W__	307	49	683
_WGY	_W__	332	47	693
_WGY	YY__	343	106	697
_WGY	_W__	440	47	706
_WGY	_RWG	442	43	707
_WGY	WB__	450	36	715
_WGY	_W__	431	67	727
_WGY	_WYG	446	77	731
_WGY	_WYG	503	111	747
_WGY	__W_	522	135	750
_WGY	_WYG	504	89	759
_WGY	_W__	458	91	764
_WGY	W___	517	107	771
_WGY	YGWY	568	137	776
_WGY	GGRG	585	373	793
_WGY	_W__curl	660	293	794
_WGY	GGRG	639	406	796
_WGY	YYBR	639	423	797
_WGY	_GYW	657	456	798
_WGY	GRY_	625	450	799
_WGY	GGRG	590	421	837
_WGY	GRGY	572	376	869
_WGY	YYYY	520	409	912
_WGY	YYYY	514	368	937
_WGY	YYYY	541	413	969
_WGY	GY_W	513	419	1041
_WGY	GGWW	706	387	1080

_WGY	GY__	661	159	1142
_WGY	GGW_right	522	95	1234
_WGY	GR_Y	490	76	1248
_WGY	_W__	487	92	1275
_WGY	G_RG	484	95	1293
_WGY	_WWY	493	112	1334
_WGY	WRR_	578	78	1586
____new	G___right	639	245	20
____new	_WGY	622	245	231
____new	____still	611	228	253
____new	_WGY	591	228	408
____new	GGW_right	593	223	647
____new	_Y__	586	226	834
____new	YYRG	581	227	876
____new	YYRG	584	225	889
____new	_WGY	585	235	1134
____new	GR_Y	584	250	1136
____new	GY__	634	218	1159
____new	GR_Y	627	213	1302
____new	YGWY	539	204	1311
____new	Q	611	218	1704
____new	YGWY	596	185	1711
G___right	____new	664	232	22
G___right	_WGY	538	207	50
G___right	_Y__	527	212	65
G___right	_WGY	565	224	208
G___right	__W_	535	184	220
G___right	__W_	530	177	238
G___right	YYRG	530	221	311
G___right	__W_	565	218	344
G___right	G__Y	585	345	357
G___right	_GYW	652	401	373
G___right	YYBR	601	430	389
G___right	GBG_	640	412	395
G___right	GRY_	672	390	400
G___right	GGRG	645	361	461
G___right	YYY_	690	364	477
G___right	YYY_	725	357	554
G___right	GGWW	712	350	641
G___right	GBG_	682	337	780
G___right	YYY_	687	322	833
G___right	_W__curl	693	292	846
G___right	_W__curl	680	299	861
G___right	_W__curl	665	247	968
G___right	_W__curl	673	249	1152
G___right	YYY_	717	250	1221
G___right	_GWY	712	219	1698
G___right	_R__right	718	229	1715
G___right	YWWW	716	154	1742

____still	____new	579	248	263
____still	GGW_right	578	243	808
____still	GR_Y	565	268	842
____still	____new	580	244	851
____still	GBG_	584	322	886
____still	GRGY	581	326	888
____still	GR_Y	539	301	982
____still	GGW_	477	304	1042
____still	_WWY	410	346	1053
____still	YYGG	418	436	1114
____still	YYGG	404	432	1124
____still	GGWW	300	447	1182
____still	YYRR	345	411	1197
____still	GRGY	398	387	1216
____still	G__Y	460	335	1227
____still	Y_B_	545	411	1237
____still	_GYW	579	368	1250
____still	GBG_	571	353	1254
____still	_GYW	538	347	1324
____still	GGGG	520	340	1341
____still	GBGR	545	310	1572
____still	_GWY	547	311	1655
____still	YYGW	541	309	1689
____still	YYBR	536	309	1699
____still	Q	526	310	1720
____still	Q	524	348	1732
GRGY	YY__	550	306	87
GRGY	G__Y	549	346	191
GRGY	GR_Y	554	319	311
GRGY	GR_Y	536	306	752
GRGY	_WGY	566	305	790
GRGY	G__Y	522	347	808
GRGY	YYYY	519	383	828
GRGY	_WGY	544	351	867
GRGY	GR_Y	515	339	874
GRGY	G__Y	503	346	878
GRGY	GBG_	573	339	884
GRGY	GGRG	589	401	901
GRGY	YYYY	558	439	911
GRGY	WR__	532	440	941
GRGY	YYGW	536	433	949
GRGY	WR__	541	470	955
GRGY	WR__	532	484	970
GRGY	_W_W	561	487	977
GRGY	YYYY	472	464	1011
GRGY	_YGG	442	473	1020
GRGY	GBGR	395	465	1047
GRGY	GBGR	365	465	1062
GRGY	G__Y	484	370	1086

GRGY	GR_Y	440	343	1093
GRGY	YGWR	551	344	1109
GRGY	GGWW	491	327	1132
GRGY	GBGR	511	357	1138
GRGY	____mid	544	374	1144
GRGY	Y_B_	555	408	1156
GRGY	G__Y	441	347	1191
GRGY	G__Y	415	367	1214
GRGY	GGGG	409	368	1220
GRGY	YYRR	348	409	1233
GRGY	WRRY	308	391	1246
GRGY	YYGG	324	384	1531
GRGY	WRRY	303	385	1556
GRGY	GYG_	272	377	1566
GRGY	WRRY	303	389	1644
GRGY	WRRY	301	376	1691
GRGY	YYGG	299	398	1702
GRGY	YYBR	358	389	1729
GRGY	YYGG	330	380	1734
_W__curl	GY__	646	260	634
_W__curl	GGWW	692	266	826
_W__curl	G___right	684	258	968
_W__curl	G___right	678	255	1137
_W__curl	G___right	687	257	1190
_W__curl	G___right	680	253	1704
_W__curl	GY__	661	256	1721
GGWW	_GYW	733	295	120
GGWW	_GYW	728	290	234
GGWW	YYY_	727	289	421
GGWW	YYY_	730	289	578
GGWW	YYY_	705	287	615
GGWW	GY__	709	279	618
GGWW	G___right	706	333	640
GGWW	YYY_	724	332	650
GGWW	_W__curl	710	260	723
GGWW	_W__curl	707	248	746
GGWW	_R__right	728	251	794
GGWW	G___right	723	292	851
GGWW	YYY_	719	332	868
GGWW	GRY_	693	343	897
GGWW	GRY_	674	348	905
GGWW	_WYG	660	416	948
GGWW	YYBR	616	431	950
GGWW	_W_W	605	449	954
GGWW	_WYG	659	450	973
GGWW	_GYW	698	418	1014
GGWW	____mid	644	389	1055
GGWW	GGRG	668	365	1068
GGWW	GRY_	689	365	1080

GGWW	_GYW	673	390	1081
GGWW	_W_W	607	426	1086
GGWW	YGWR	629	369	1104
GGWW	GBG_	590	330	1118
GGWW	YYGW	535	298	1127
GGWW	GGW_	457	327	1137
GGWW	____mid	344	418	1158
GGWW	____mid	276	440	1181
GGWW	WGGB	268	427	1183
GGWW	WRRY	308	395	1191
GGWW	YYRR	304	397	1196
GGWW	GGGG	346	354	1203
GGWW	YY__	272	195	1211
GGWW	_WWY	274	198	1215
GGWW	_GWY	284	178	1227
GGWW	YY_W	303	161	1503
GGWW	YY_W	301	149	1540
GGWW	YYRB	273	256	1561
GGWW	GB__	298	285	1566
GGWW	WRRY	329	332	1588
GGWW	_GWY	420	436	1611
GGWW	WR__	542	480	1638
GGWW	YYBR	550	485	1642
GGWW	_W_W	584	476	1665
GGWW	_WWG	621	480	1668
GGWW	GRY_	655	401	1695
YGWR	GGRG	630	318	708
YGWR	____new	602	246	1005
YGWR	YYGW	583	273	1010
YGWR	YYGW	580	270	1018
YGWR	G___right	618	262	1021
YGWR	GBG_	609	290	1056
YGWR	____mid	618	270	1063
YGWR	YYGW	549	296	1079
YGWR	GGRG	630	347	1091
YGWR	GBG_	628	317	1113
YGWR	GRY_	643	332	1131
YGWR	YYGW	533	314	1164
YGWR	YYGW	542	294	1173
YGWR	YYGW	502	265	1238
YGWR	GGW_	493	277	1247
YGWR	GBGR	541	302	1253
YGWR	YYGW	558	306	1284
YGWR	GR_Y	598	275	1295
YGWR	G___right	663	241	1313
YGWR	____new	634	214	1349
YGWR	YGWY	531	209	1352
YGWR	GY__	647	227	1355
YGWR	GBG_	619	291	1373

YGWR	_W__curl	684	310	1546
YGWR	GRY_	685	310	1616
YGWR	_W__curl	685	300	1631
YGWR	_GYW	679	315	1681
YGWR	GY__	685	310	1713
YGWR	GBG_	644	298	1743
G__Y	YYYY	522	391	3
G__Y	GGRG	567	374	36
G__Y	YY__	547	352	84
G__Y	GGRG	606	363	93
G__Y	GRGY	548	362	267
G__Y	GR_Y	491	332	654
G__Y	YYGW	477	331	874
G__Y	_WGY	481	334	910
G__Y	GR_Y	480	339	979
G__Y	Y_B_	530	364	1081
G__Y	____mid	525	397	1095
G__Y	GGGG	385	353	1181
G__Y	GR_Y	409	350	1213
G__Y	GGGG	458	374	1256
G__Y	GY_W	412	369	1345
G__Y	____mid	415	370	1347
G__Y	____mid	412	367	1706
G__Y	GGGG	409	366	1731
_GYW	GGRG	641	380	3
_GYW	GRY_	658	387	22
_GYW	YYY_	660	371	27
_GYW	YYY_	677	346	44
_GYW	YYY_	703	331	71
_GYW	_W__curl	666	291	102
_GYW	YYY_	705	326	118
_GYW	GGWW	717	317	120
_GYW	GRY_	676	351	157
_GYW	GRY_	671	334	182
_GYW	_W__curl	679	292	190
_GYW	_W__curl	687	283	228
_GYW	GGWW	703	287	234
_GYW	YYY_	704	329	256
_GYW	GRY_	663	356	290
_GYW	GGRG	653	376	332
_GYW	_WWG	642	425	346
_GYW	_WWG	658	430	353
_GYW	YYBR	649	442	364
_GYW	GRY_	681	435	379
_GYW	GRY_	731	415	424
_GYW	_WWG	685	463	491
_GYW	_WWG	675	470	505
_GYW	_WWG	641	480	554
_GYW	_W_W	617	483	581

_GYW	GBG_	605	455	601
_GYW	_WWG	670	480	633
_GYW	_WWG	682	480	695
_GYW	_WWG	663	475	767
_GYW	GRY_	647	474	776
_GYW	_WWG	663	482	780
_GYW	_WWG	711	456	869
_GYW	YYBR	704	451	876
_GYW	YYY_	734	396	986
_GYW	GGWW	717	400	1013
_GYW	GGWW	682	410	1080
_GYW	GGRG	672	406	1140
_GYW	_WWG	659	428	1218
_GYW	GRY_	643	359	1240
_GYW	GGGG	535	351	1283
_GYW	Y_B_	531	390	1431
_GYW	GGGG	517	381	1463
_GYW	Y_B_	551	397	1480
_GYW	GBGR	565	390	1561
_GYW	GBG_	571	346	1609
_GYW	_GWY	591	361	1630
_GYW	GBG_	570	305	1635
_GYW	YYGW	570	306	1637
_GYW	YGWR	655	328	1681
_GYW	_W__curl	649	286	1690
_GYW	_W__curl	661	299	1701
_GYW	YGWR	675	304	1704
_GYW	GY__	657	302	1706
_GYW	YYGW	610	368	1743
_GYW	GBG_	607	332	1746
GGRG	G__Y	601	384	94
GGRG	YYBR	642	362	651
GGRG	YGWR	638	351	680
GGRG	YGWR	597	351	698
GGRG	YYBR	637	392	741
GGRG	GRY_	606	400	746
GGRG	YYBR	616	404	789
GGRG	_WGY	584	379	793
GGRG	GRY_	636	329	889
GGRG	GBG_	619	321	895
GGRG	GRGY	604	384	901
GGRG	GRGY	584	408	927
GGRG	YYYY	583	362	943
GGRG	YGWR	638	299	1006
GGRG	G___right	641	277	1015
GGRG	_W__curl	652	284	1018
GGRG	YYGW	656	336	1058
GGRG	GRY_	659	340	1063
GGRG	GGWW	657	352	1068

GGRG	_WGY	657	337	1073
GGRG	GBG_	628	334	1085
GGRG	_GYW	670	392	1138
GGRG	____mid	607	375	1155
GGRG	Y_B_	587	409	1162
GGRG	YYYY	509	418	1187
GGRG	GBG_	578	318	1232
GGRG	GBGR	578	322	1241
GGRG	G__Y	589	341	1254
GGRG	YYGW	558	290	1324
GGRG	YYRG	546	183	1338
GGRG	WRR_	494	146	1346
GGRG	_WWY	507	153	1481
GGRG	____new	590	198	1522
GGRG	WRR_	507	154	1559
GGRG	_WWY	489	131	1575
GGRG	YYRG	513	178	1582
GGRG	_WWY	531	156	1589
GGRG	WRR_	526	138	1594
GGRG	YGWY	574	195	1622
GGRG	____new	605	221	1636
GGRG	____new	606	220	1678
GGRG	W_WR	511	173	1719
YYY_	_GYW	699	343	46
YYY_	_GYW	715	346	72
YYY_	_GYW	713	354	264
YYY_	GGWW	724	309	422
YYY_	G___right	687	348	476
YYY_	GGWW	725	309	517
YYY_	G___right	730	389	701
YYY_	_WWG	711	432	769
YYY_	G___right	709	331	845
YYY_	G___right	707	341	863
YYY_	GGWW	711	344	877
YYY_	_W__curl	705	267	1161
YYY_	_W__curl	713	271	1257
GRY_	YYBR	661	428	38
GRY_	GGRG	650	383	46
GRY_	_GYW	664	366	50
GRY_	YYY_	665	389	129
GRY_	_GYW	663	372	156
GRY_	_GYW	653	351	183
GRY_	YGWR	641	333	212
GRY_	_GYW	680	419	341
GRY_	_WWG	686	421	346
GRY_	_GYW	716	401	427
GRY_	YYBR	666	424	538
GRY_	YYBR	672	411	549
GRY_	_WWG	688	447	620

GRY_	G___	699	398	661
GRY_	YYBR	669	415	691
GRY_	_WWY	661	418	709
GRY_	GBG_	659	418	736
GRY_	_GYW	623	471	778
GRY_	_W_W	616	448	784
GRY_	_WGY	611	438	811
GRY_	GGRG	652	344	892
GRY_	_WGY	702	360	1080
GRY_	GGRG	690	351	1106
GRY_	YGWR	672	336	1129
GRY_	GBGR	668	328	1352
GRY_	_W__curl	667	290	1384
GRY_	GY__	666	310	1388
GRY_	_WWG	672	439	1535
GRY_	YGWR	672	331	1616
GRY_	_GYW	653	380	1680
_WWG	YYY_	709	416	55
_WWG	_YGG	734	450	89
_WWG	_YGG	678	487	189
_WWG	YYBR	662	455	256
_WWG	YYBR	669	455	326
_WWG	_GYW	673	437	354
_WWG	_GYW	672	455	383
_WWG	_GYW	671	458	517
_WWG	_WYG	652	453	548
_WWG	_WGY	664	461	804
_WWG	YYBR	670	468	825
_WWG	GGWW	658	451	950
_WWG	GGWW	660	467	962
_WWG	_W_W	662	453	1061
_WWG	YYBR	649	463	1131
_WWG	_W_W	635	458	1321
_WWG	YYBR	633	456	1350
_WWG	_W_W	618	454	1398
_WWG	YYBR	632	485	1594
_WWG	_W_W	615	480	1623
_WWG	GGWW	666	474	1687
GBG_	YYBR	608	436	492
GBG_	GRY_	631	415	712
GBG_	GGRG	627	389	734
GBG_	G___right	661	314	780
GBG_	YGWR	616	307	785
GBG_	YYGW	604	296	981
GBG_	GBGR	601	298	1246
GBG_	_GYW	596	302	1637
GBG_	_GWY	603	300	1670
GBG_	GY__	607	304	1710
GBG_	_GYW	587	290	1748

YYBR	GRY_	630	441	38
YYBR	_YGG	634	461	97
YYBR	GRY_	635	446	101
YYBR	_WWG	638	451	216
YYBR	_WWG	641	448	326
YYBR	_GYW	648	437	347
YYBR	_W_W	622	458	381
YYBR	_W_W	614	470	393
YYBR	_WWG	634	458	445
YYBR	_WWG	630	458	475
YYBR	_W_W	601	472	486
YYBR	GBG_	611	460	508
YYBR	_WWG	632	438	533
YYBR	GRY_	640	418	537
YYBR	G___right	659	376	548
YYBR	GGRG	649	379	600
YYBR	GRY_	676	395	619
YYBR	GRY_	665	396	689
YYBR	GRY_	692	439	705
YYBR	_WWG	689	456	722
YYBR	YYY_	696	437	734
YYBR	_WWG	669	426	742
YYBR	GRY_	659	431	772
YYBR	_WWG	656	452	847
YYBR	_W_W	617	479	914
YYBR	_W_W	609	489	1002
YYBR	Y_B_	594	482	1177
YYBR	_W_W	605	489	1197
YYBR	Y_B_	575	483	1234
YYBR	WR__	525	475	1259
YYBR	_WWG	620	489	1325
YYBR	_WWG	630	488	1366
YYBR	_WWG	623	488	1415
YYBR	_W_W	604	480	1505
YYBR	_WWG	596	491	1511
YYBR	_WWG	601	485	1594
YYBR	WR__	576	477	1638
YYBR	GGWW	574	471	1646
YYBR	WR__	571	460	1651
YYBR	YYGW	549	313	1680
YYBR	YYGW	569	294	1703
YYBR	_GWY	563	302	1704
YYBR	Q	572	303	1710
YYBR	Q	551	330	1718
YYBR	YYGW	476	367	1720
YYBR	GRGY	374	405	1728
YYBR	YYGG	322	430	1743
YYYY	_W_W	564	441	735
YYYY	_WGY	547	434	827

YYYY	WR__	539	433	831
YYYY	GRY_	517	396	935
YYYY	_WGY	532	355	940
YYYY	GGRG	561	362	944
YYYY	_WGY	554	425	969
YYYY	GRY_	544	435	985
YYYY	Y_B_	521	404	996
YYYY	WR__	484	450	1007
YYYY	_WWY	404	360	1037
YYYY	GRGY	421	416	1041
YYYY	WR__	486	457	1070
YYYY	GRGY	460	385	1092
YYYY	GGWW	464	421	1148
YYYY	G__Y	462	388	1152
YYYY	____mid	479	414	1177
YYYY	GBGR	503	431	1276
YYYY	YYGG	444	437	1286
YYYY	YYGG	444	439	1317
YYYY	_GWY	444	436	1582
YYYY	_GWY	497	434	1615
YYYY	WR__	527	439	1697
YYYY	WR__	539	474	1737
YYYY	WR__	540	484	1745
YYYY	_GWY	519	486	1749
GGGG	WR__	534	463	116
GGGG	YYYY	536	442	343
GGGG	YYGW	373	350	359
GGGG	____mid	388	348	363
GGGG	GRBR	307	303	370
GGGG	____hidden	284	264	374
GGGG	YYGG	270	257	453
GGGG	____hidden	264	288	458
GGGG	____hidden	275	275	474
GGGG	WRRY	271	262	507
GGGG	____hidden	266	243	522
GGGG	GB__	290	272	532
GGGG	YYGG	306	305	546
GGGG	GRBR	304	327	584
GGGG	YYGW	345	370	649
GGGG	GB__	313	338	757
GGGG	____mid	348	359	811
GGGG	YYGW	362	378	839
GGGG	WRRY	339	341	856
GGGG	WRRY	319	331	922
GGGG	YY__	329	289	982
GGGG	GRBR	336	303	983
GGGG	YY__	305	289	1026
GGGG	GBGR	337	366	1082
GGGG	GBGR	377	363	1128

GGGG	GRGY	389	345	1219
GGGG	G__Y	414	362	1231
GGGG	YYGG	430	422	1242
GGGG	YYYY	449	401	1253
GGGG	G__Y	451	385	1256
GGGG	_GYW	502	357	1285
GGGG	_GYW	502	363	1430
GGGG	YYYY	477	396	1441
GGGG	____mid	461	355	1446
GGGG	_GYW	500	360	1463
GGGG	GBGR	519	351	1473
GGGG	____mid	451	342	1497
GGGG	GGRG	522	333	1518
GGGG	G__Y	456	361	1526
GGGG	____mid	461	356	1531
GGGG	GRBR	387	320	1543
GGGG	YYGG	382	378	1547
GGGG	_GWY	329	364	1560
GGGG	GBGR	274	272	1735
WR__	_YGG	534	464	38
WR__	YYYY	540	463	829
WR__	YYYY	535	458	845
WR__	GRGY	539	454	950
WR__	GRGY	522	477	1003
WR__	Y_B_	531	464	1125
WR__	Y_B_	531	488	1182
WR__	GBGR	518	434	1282
WR__	GBGR	535	441	1324
WR__	YYBR	554	478	1635
WR__	GGWW	557	472	1671
_W_W	_YGG	597	472	49
_W_W	_YGG	593	471	161
_W_W	YYBR	593	472	382
_W_W	YYBR	594	471	505
_W_W	_GYW	590	472	565
_W_W	GRY_	587	466	784
_W_W	GRGY	591	467	985
_W_W	YYBR	584	465	1007
_W_W	YYBR	609	464	1041
_W_W	YYBR	618	449	1056
_W_W	_WWG	640	440	1061
_W_W	GGWW	631	406	1072
_W_W	Y_B_	592	459	1297
_W_W	_WWG	594	455	1399
_W_W	_WWG	592	455	1624
_YGG	WR__	563	484	37
_YGG	YY__	557	485	38
_YGG	_WWG	716	475	89
_YGG	YYBR	662	484	97

_YGG	_W_W	620	481	137
_YGG	YYBR	654	469	152
_YGG	_W_W	613	486	161
_YGG	WGGB	347	490	325
_YGG	WR__	509	490	1139
_YGG	YYGG	349	483	1426
_YGG	WGGB	355	487	1570
_YGG	_GWY	437	479	1738

Antlist	InitialPosX	InitialPosY	ColonyArea	AntLength	MinX	MinY	MaxX	MaxY
_W_R	270	67	280476	52.7	161	24	775	509
WRRY	250	188						
_WRW	198	152						
Y___	218	187						
GR_W	206	235						
____hidden	241	256						
YYG_	250	299						
YYGW	258	370						
GYG_	237	394						
_R__	180	388						
YYRR	255	444						
YY__	331	470						
WGGB	300	436						
GRBR	346	334						
GB__	296	316						
YYGG	340	195						
W_WR	309	184						
_WWY	423	128						
GG_W	403	203						
YYRB	365	274						
____white	366	278						
____mid	398	330						
GBGR	372	432						
Y_B_	401	392						
GY_W	486	404						
GGW_	447	296						
Q	465	191						
GGGR	442	222						
GYY_	462	220						
YYRG	489	272						
_Y__	488	207						
YY_W	445	141						
__W_	489	169						
_WYG	488	134						
_W__	480	80						
G___	392	40						
WB__	512	36						
_RWG	460	0						
WBYG	574	34						
GGW_right	633	82						
G_RG	622	140						
YGWY	617	133						
_WGY	654	208						
GY__	688	209						
____new	639	243						
GG__	742	142						
W___	677	40						
_R__right	739	192						
G___right	715	242						
____still	580	247						
_W__curl	645	292						
GGWW	733	296						
GR_Y	499	284						
GRGY	548	297						
YGWR	629	316						
YYY_	699	342						
_GYW	642	380						
GGRG	605	390						
G__Y	513	393						
YYYY	557	426						
GGGG	540	440						
WR__	537	458						
GRY_	646	417						
GBG_	499	434						
YYBR	625	438						
_W_W	598	476						
_YGG	570	485						
_WWG	735	449						
_GWY	444	0						
YWWW	450	0						
WRR_	451	0						
Y_BW	452	0						

Actor	Target	Time	ActorPosX	ActorPosY
_____1	DIE	7	0	0
_____1	WRGW	16	0	0
_____1	WYWW	103	0	0
_____1	WGWY	144	0	0
_____1	WG__2	160	0	0
_____1	WWGY	351	0	0
_____1	WGWY	381	0	0
_____1	WRGW	411	0	0
_____1	WWWY	421	0	0
_____1	WGGG	527	0	0
_____1	_____2	942	0	0
_____1	WYRW	951	0	0
_____1	WGG_	1104	0	0
_____1	WWR_	1154	0	0
_____1	W_W_2	1420	0	0
_____2	WG__1	177	0	0
_____2	WYYG	393	0	0
_____2	W_W_2	432	0	0
_____2	WGGY	557	0	0
_____2	Q	952	0	0
_____2	WWGY	962	0	0
_____2	WG__1	968	0	0
_____2	WG__2	976	0	0
_____2	WYYG	985	0	0
_____2	WBWY	986	0	0
_____2	WGYY	994	0	0
_____2	WYRW	1007	0	0
_____2	WYRW	1231	0	0
_____2	Q	1350	0	0
_____3	_GGR	306	0	0
_____3	_GGR	313	0	0
_____3	WW__2	750	0	0
_____3	W_W_1	963	0	0
_____3	WGGW	1121	0	0
_____3	WGGW	1147	0	0
_____3	_GGR	1154	0	0
_GGR	WGRP	13	0	0
_GGR	WYWW	14	0	0
_GGR	WY__	43	0	0
_GGR	WYWW	76	0	0
_GGR	WY__	80	0	0
_GGR	WY__	83	0	0
_GGR	WY_R	129	0	0
_GGR	WR__	144	0	0
_GGR	W_RG	164	0	0
_GGR	WW__(H)	178	0	0
_GGR	WW__3	253	0	0
_GGR	_____3	306	0	0

_GGR	WYRR(DIE)	350	0	0
_GGR	WW__	372	0	0
_GGR	WYYG	385	0	0
_GGR	_YGW	394	0	0
_GGR	WY__	408	0	0
_GGR	WY_R	416	0	0
_GGR	W_YY	427	0	0
_GGR	WGWY	531	0	0
_GGR	WRGG	545	0	0
_GGR	WG__2	551	0	0
_GGR	WYG_	573	0	0
_GGR	WW__(H)	597	0	0
_GGR	WRGW	600	0	0
_GGR	WWWY	618	0	0
_YGW	WW__(Z)	22	0	0
_YGW	WW__(H)	387	0	0
_YGW	_GGR	393	0	0
_YGW	WW__	828	0	0
_YGW	WW__	834	0	0
_YGW	WW__	864	0	0
_YYY	Q	40	0	0
_YYY	Q	52	0	0
_YYY	WYY_	77	0	0
_YYY	WWY_	104	0	0
_YYY	WRWP	132	0	0
_YYY	WGWW	266	0	0
_YYY	W_YY	287	0	0
_YYY	WYY_	323	0	0
_YYY	WR__	592	0	0
_YYY	WBGW	1041	0	0
_YYY	WGGY	1365	0	0
_YYY	WGGY	1382	0	0
_YYY	WGGY	1395	0	0
PYPG	WG__2	739	0	0
PYPG	WGRP	777	0	0
PYPG	WW__	857	0	0
PYPG	WWWY	863	0	0
PYPG	WGG_	875	0	0
PYPG	WG__2	954	0	0
PYPG	WGYY	956	0	0
PYPG	WGYY	961	0	0
PYPG	W___1	964	0	0
PYPG	WG__1	986	0	0
PYPG	W_W_1	1007	0	0
PYPG	W___1	1037	0	0
PYPG	WPPP	1040	0	0
PYPG	W___1	1044	0	0
PYPG	W___1	1062	0	0
PYPG	_GGR	1106	0	0

PYPG	WPGR	1115	0	0
PYPG	WRWP	1125	0	0
PYPG	WGWW	1132	0	0
PYPG	WGWY	1143	0	0
PYPG	WWY_	1149	0	0
PYPG	WG__1	1153	0	0
PYPG	WPGG	1156	0	0
PYPG	W_W_1	1171	0	0
PYPG	WYY_	1185	0	0
PYPG	WYG_	1194	0	0
PYPG	WYG_	1204	0	0
PYPG	WGG_	1207	0	0
PYPG	WG__1	1235	0	0
PYPG	____2	1245	0	0
PYPG	W___1	1309	0	0
PYPG	WPPP	1329	0	0
PYPG	WPPP	1349	0	0
Q	WRYR	8	0	0
Q	WW__1	35	0	0
Q	_YYY	38	0	0
Q	WR__	42	0	0
Q	WW__1	63	0	0
Q	WR__	67	0	0
Q	_YYY	73	0	0
Q	WR__	78	0	0
Q	WG__1	93	0	0
Q	WWR_	106	0	0
Q	WWR_	109	0	0
Q	WG_Y	155	0	0
Q	WPGR	168	0	0
Q	W_RG	245	0	0
Q	WGG_	268	0	0
Q	WG__2	272	0	0
Q	WW__(H)	281	0	0
Q	WG__2	293	0	0
Q	WPGG	309	0	0
Q	WBWY	320	0	0
Q	WWRY	361	0	0
Q	WRGG	362	0	0
Q	W_W_2	380	0	0
Q	WYG_	397	0	0
Q	WYG_	408	0	0
Q	WW__(H)	435	0	0
Q	WWRY	469	0	0
Q	W_W_1	489	0	0
Q	WG__2	508	0	0
Q	WPGY	519	0	0
Q	WW__	559	0	0
Q	WWGY	663	0	0

Q	WPGG	861	0	0
Q	W_W_2	869	0	0
Q	WPGG	920	0	0
Q	_YYY	1002	0	0
Q	W_BG	1032	0	0
Q	W_BG	1108	0	0
Q	WWRY	1141	0	0
Q	W_BG	1143	0	0
Q	WPGR	1365	0	0
Q	WYYG	1393	0	0
W___1	_YYY	282	0	0
W___1	WGWW	288	0	0
W___1	WGWW	315	0	0
W___1	WGGG	380	0	0
W___1	WGGG	436	0	0
W___1	WGGG	613	0	0
W___1	WGGG	658	0	0
W___1	WW__	687	0	0
W___1	WGGW	1255	0	0
W___1	WW__(Z)	1438	0	0
W_BG	W_YY	0	0	0
W_BG	WY_R	369	0	0
W_BG	WBGW	414	0	0
W_BG	WGGG	535	0	0
W_BG	W_RG	567	0	0
W_BG	WYRW	854	0	0
W_BG	Q	1108	0	0
W_BG	W_YY	1326	0	0
W_BG	WBGW	1348	0	0
W_BG	W_YY	1366	0	0
W_BG	W_YR	1395	0	0
W_RG	W_YY	386	0	0
W_RG	WGGG	425	0	0
W_RG	Q	517	0	0
W_RG	W_BG	561	0	0
W_RG	WGWW	595	0	0
W_RG	WG__1	730	0	0
W_RG	W_BG	778	0	0
W_RG	W_W_1	934	0	0
W_RG	_GGR	1195	0	0
W_RG	WR__	1214	0	0
W_RG	WPGR	1248	0	0
W_RG	WPGR	1257	0	0
W_W_1	WRWP	388	0	0
W_W_1	WRGG	978	0	0
W_W_1	WPGG	1110	0	0
W_W_1	WWR_	1138	0	0
W_W_1	WYWW	1166	0	0
W_W_1	WGRP	1168	0	0

W_W_1	PYPG	1170	0	0
W_W_2	_GGR	0	0	0
W_W_2	WRGW	64	0	0
W_W_2	WW__	878	0	0
W_W_2	Q	964	0	0
W_W_2	WW__	1024	0	0
W_W_2	WW__	1082	0	0
W_W_2	WWRY	1128	0	0
W_W_2	W_YR	1149	0	0
W_W_2	_GGR	1323	0	0
W_W_2	W_YR	1353	0	0
W_W_2	W_YR	1406	0	0
W_YR	WRGG	336	0	0
W_YR	W_BG	425	0	0
W_YR	WW__	458	0	0
W_YR	W___1	492	0	0
W_YR	WG__1	509	0	0
W_YR	WG__1	526	0	0
W_YR	W_W_2	549	0	0
W_YR	WGG_	607	0	0
W_YR	____1	957	0	0
W_YR	WW__(Z)	1034	0	0
W_YR	WW__(Z)	1050	0	0
W_YR	____1	1056	0	0
W_YR	WYRW	1076	0	0
W_YR	WYRW	1089	0	0
W_YR	W_W_2	1149	0	0
W_YR	WGRP	1218	0	0
W_YR	W_W_2	1353	0	0
W_YR	W_W_1	1375	0	0
W_YR	W_BG	1396	0	0
W_YR	W_W_2	1406	0	0
W_YY	W_BG	0	0	0
W_YY	WGWW2	262	0	0
W_YY	_YYY	273	0	0
W_YY	_YYY	286	0	0
W_YY	_YYY	294	0	0
W_YY	WY_R	335	0	0
W_YY	W_BG	358	0	0
W_YY	____2	557	0	0
W_YY	W_W_2	663	0	0
W_YY	____2	790	0	0
W_YY	W_W_2	837	0	0
W_YY	W_W_2	867	0	0
W_YY	WYRW	963	0	0
W_YY	WPGG	977	0	0
W_YY	WBWY	1234	0	0
W_YY	W_BG	1322	0	0
W_YY	_YYY	1395	0	0

W_YY	WGRP	1430	0	0
WBWY	WG__1	183	0	0
WBWY	WW__1	288	0	0
WBWY	Q	319	0	0
WBWY	WYWW	343	0	0
WBWY	WG__3	435	0	0
WBWY	WG__3	481	0	0
WBWY	WG__3	489	0	0
WBWY	WYYB	895	0	0
WBWY	WWRY	903	0	0
WBWY	_GGR	923	0	0
WBWY	WPGR	965	0	0
WBWY	WWGY	1227	0	0
WBWY	WYG_	1273	0	0
WG__1	WRGG	119	0	0
WG__1	WRWP	139	0	0
WG__1	WRWP	140	0	0
WG__1	WRGG	146	0	0
WG__1	____2	177	0	0
WG__1	WGG_	412	0	0
WG__1	WGYY	464	0	0
WG__1	W_YR	501	0	0
WG__1	WGWY	567	0	0
WG__1	WPGG	712	0	0
WG__1	PYPG	818	0	0
WG__1	WWGY	822	0	0
WG__1	WGRP	879	0	0
WG__1	WWR_	924	0	0
WG__1	WGG_	936	0	0
WG__1	WWR_	973	0	0
WG__1	WW__	979	0	0
WG__1	WWGY	984	0	0
WG__1	WWR_	1120	0	0
WG__1	WRYR	1130	0	0
WG__1	WGG_	1250	0	0
WG__1	WRYY	1281	0	0
WG__1	WYY_	1323	0	0
WG__1	WGGG	1356	0	0
WG__1	WYY_	1374	0	0
WG__1	WWR_	1401	0	0
WG__1	WGYY	1407	0	0
WG__2	WGRP	0	0	0
WG__2	WYRW	75	0	0
WG__2	Q	292	0	0
WG__2	W_W_1	404	0	0
WG__2	WWR_	536	0	0
WG__2	_GGR	554	0	0
WG__2	WRGG	570	0	0
WG__2	WPYY	574	0	0

WG__2	WGWY	620	0	0
WG__2	WYRW	663	0	0
WG__2	W___1	687	0	0
WG__2	WW__2	703	0	0
WG__2	W_RG	724	0	0
WG__2	PYPG	736	0	0
WG__2	PYPG	739	0	0
WG__2	WGRP	772	0	0
WG__2	PYPG	787	0	0
WG__2	PYPG	799	0	0
WG__2	PYPG	821	0	0
WG__2	WW__	955	0	0
WG__2	WYRW	985	0	0
WG__2	WW__2	1013	0	0
WG__2	WRYY	1034	0	0
WG__2	WYG_	1072	0	0
WG_Y	WWGY	219	0	0
WG_Y	WGG_	373	0	0
WG_Y	WGGY	424	0	0
WG_Y	WWWY	467	0	0
WG_Y	____2	992	0	0
WGG_	WRYR	16	0	0
WGG_	WG__1	44	0	0
WGG_	WGRP	129	0	0
WGG_	WY_G2	149	0	0
WGG_	WGWY	174	0	0
WGG_	WGWY	179	0	0
WGG_	WGWY	266	0	0
WGG_	WWGY	302	0	0
WGG_	WY__	316	0	0
WGG_	WY__	320	0	0
WGG_	WG_Y	376	0	0
WGG_	WGWY	385	0	0
WGG_	WG__1	411	0	0
WGG_	W_YR	507	0	0
WGG_	W_W_1	510	0	0
WGG_	WGWY	529	0	0
WGG_	Q	568	0	0
WGG_	WG__1	575	0	0
WGG_	W___1	588	0	0
WGG_	_GGR	658	0	0
WGG_	W_W_1	699	0	0
WGG_	WG__1	712	0	0
WGG_	WG__1	767	0	0
WGG_	WWWY	879	0	0
WGG_	WY_G2	948	0	0
WGG_	____1	1146	0	0
WGG_	_GGR	1263	0	0
WGG_	WW__2	1336	0	0

WGG_	WBGW	1404	0	0
WGGG	W___1	383	0	0
WGGG	W_RG	430	0	0
WGGG	W___1	433	0	0
WGGG	W___1	624	0	0
WGGG	WYRW	711	0	0
WGGG	WPYY	731	0	0
WGGG	WPGR	747	0	0
WGGG	WGWY	893	0	0
WGGG	WR__	914	0	0
WGGG	WR__	967	0	0
WGGG	WYY_	987	0	0
WGGG	W_BG	1069	0	0
WGGG	W_BG	1103	0	0
WGGW	Q	972	0	0
WGGW	Q	992	0	0
WGGW	Q	1016	0	0
WGGW	W_RG	1020	0	0
WGGW	WYY_	1022	0	0
WGGW	WPGR	1036	0	0
WGGW	WPGR	1045	0	0
WGGW	____3	1088	0	0
WGGY	WG__1	23	0	0
WGGY	W_RG	35	0	0
WGGY	WGGG	112	0	0
WGGY	WGWW	113	0	0
WGGY	WG__1	132	0	0
WGGY	WPYY	133	0	0
WGGY	WRGG	149	0	0
WGGY	Q	157	0	0
WGGY	WWR_	189	0	0
WGGY	WWY_	194	0	0
WGGY	WYGR	221	0	0
WGGY	WRYY	228	0	0
WGGY	WPGG	258	0	0
WGGY	WWGY	263	0	0
WGGY	PYPG	335	0	0
WGGY	_GGR	371	0	0
WGGY	WGG_	424	0	0
WGGY	WRYR	433	0	0
WGGY	WGG_	443	0	0
WGGY	WGG_	462	0	0
WGGY	WGYY	489	0	0
WGGY	WGG_	507	0	0
WGGY	W_W_1	520	0	0
WGGY	WGG_	540	0	0
WGGY	WGG_	565	0	0
WGGY	Q	568	0	0
WGGY	WG__1	574	0	0

WGGY	WY_G2	957	0	0
WGGY	WG__1	967	0	0
WGGY	WPGG	981	0	0
WGGY	WGG_	989	0	0
WGGY	WW__3	996	0	0
WGGY	WW__3	1003	0	0
WGGY	WWWY	1081	0	0
WGGY	WYRR(DIE)	1130	0	0
WGGY	WGYY	1269	0	0
WGGY	___W	1346	0	0
WGRP	WYWW	357	0	0
WGRP	WG__1	879	0	0
WGRP	WG__1	894	0	0
WGRP	WYWW	1301	0	0
WGRP	WYWW	1344	0	0
WGRP	WYWW	1358	0	0
WGWW	WWR_	5	0	0
WGWW	W_W_1	12	0	0
WGWW	WY_R	17	0	0
WGWW	WGGG	61	0	0
WGWW	WGGG	149	0	0
WGWW	W___1	163	0	0
WGWW	WPGR	174	0	0
WGWW	_YYY	261	0	0
WGWW	WGWY	283	0	0
WGWW	W___1	296	0	0
WGWW	W_YY	309	0	0
WGWW	W___1	316	0	0
WGWW	W_BG	358	0	0
WGWW	WGWY	496	0	0
WGWW	WPPP	627	0	0
WGWW	W___1	876	0	0
WGWW	W___1	882	0	0
WGWW	WW__	927	0	0
WGWW	W_YR	1008	0	0
WGWW	WYYG	1016	0	0
WGWW	WBWY	1027	0	0
WGWW	WGGW	1082	0	0
WGWW	WW__2	1088	0	0
WGWW	WGRP	1091	0	0
WGWW	WRWP	1286	0	0
WGWW	WR__	1381	0	0
WGWW	WY_R	1408	0	0
WGWW	W_RG	1438	0	0
WGWY	WWGG	36	0	0
WGWY	_YGW	66	0	0
WGWY	____1	145	0	0
WGWY	WGGG	228	0	0
WGWY	WY_R	245	0	0

WGWY	WYRW	253	0	0
WGWY	WR__	258	0	0
WGWY	WYRW	264	0	0
WGWY	WPGR	498	0	0
WGWY	WPGR	532	0	0
WGWY	_GGR	537	0	0
WGWY	WPGR	551	0	0
WGWY	WW__1	1062	0	0
WGWY	WRWP	1164	0	0
WGWY	WW__	1172	0	0
WGWY	WPGR	1183	0	0
WGWY	_GGR	1210	0	0
WGWY	WPYY	1220	0	0
WGWY	WPYY	1253	0	0
WGYY	WWGY	9	0	0
WGYY	WWGY	62	0	0
WGYY	WW__2	167	0	0
WGYY	W___1	212	0	0
WGYY	W_W_1	364	0	0
WGYY	WG__1	378	0	0
WGYY	WRYR	385	0	0
WGYY	W_W_1	389	0	0
WGYY	W___1	404	0	0
WGYY	W___1	609	0	0
WGYY	_GGR	624	0	0
WGYY	WWWY	655	0	0
WGYY	WWWY	690	0	0
WGYY	WGRP	711	0	0
WGYY	WWWY	785	0	0
WGYY	WW__	818	0	0
WGYY	W_YR	831	0	0
WGYY	WG__1	855	0	0
WGYY	WRYR	903	0	0
WGYY	W_YR	918	0	0
WGYY	WRYR	1293	0	0
WGYY	WWGY	1401	0	0
WPGG	WY_G2	89	0	0
WPGG	Q	309	0	0
WPGG	WWR_	430	0	0
WPGG	WW__(H)	495	0	0
WPGG	WG__1	714	0	0
WPGG	WWR_	747	0	0
WPGG	W_W_2	814	0	0
WPGG	_GGR	833	0	0
WPGG	Q	867	0	0
WPGG	_GGR	884	0	0
WPGG	Q	912	0	0
WPGG	WW__	935	0	0
WPGG	WWRY	967	0	0

WPGG	WGGY	981	0	0
WPGR	WGWW	501	0	0
WPGR	WGWY	502	0	0
WPGR	WGWY	529	0	0
WPGR	W_W_1	660	0	0
WPGR	W_W_1	673	0	0
WPGR	WYRW	698	0	0
WPGR	_GGR	778	0	0
WPGR	WYRW	787	0	0
WPGR	WYRW	809	0	0
WPGR	WGGW	1034	0	0
WPGR	WGGW	1045	0	0
WPGR	WGWY	1188	0	0
WPGR	WW__2	1197	0	0
WPGR	WBGW	1213	0	0
WPGR	W_RG	1247	0	0
WPGY	WRWP	0	0	0
WPGY	_YYY	250	0	0
WPGY	WY_R	945	0	0
WPGY	_YYY	996	0	0
WPGY	_YYY	1010	0	0
WPGY	WY_R	1437	0	0
WPPP	WW__3	0	0	0
WPYY	WRGG	0	0	0
WPYY	WY_R	287	0	0
WPYY	_GGR	817	0	0
WPYY	WW__1	937	0	0
WPYY	WW__1	1053	0	0
WPYY	WWY_	1197	0	0
WPYY	WY_R	1244	0	0
WPYY	WGWY	1303	0	0
WPYY	WGWY	1321	0	0
WR__	WG__2	361	0	0
WR__	WPYY	456	0	0
WR__	WPYY	467	0	0
WR__	WY_R	476	0	0
WR__	WPYY	477	0	0
WR__	WGGG	602	0	0
WR__	WGGG	914	0	0
WR__	WGGG	966	0	0
WR__	W_RG	967	0	0
WR__	WY_G1	977	0	0
WR__	WGWW	1274	0	0
WR__	WBGW	1350	0	0
WRGG	WPYY	41	0	0
WRGG	WWR_	52	0	0
WRGG	WG__1	145	0	0
WRGG	W___1	274	0	0
WRGG	W___1	276	0	0

WRGG	WG__1	340	0	0
WRGG	WWY_	343	0	0
WRGG	WG__1	348	0	0
WRGG	WWY_	652	0	0
WRGG	WWY_	730	0	0
WRGG	WWY_	782	0	0
WRGG	WRWP	949	0	0
WRGG	WRWP	976	0	0
WRGG	WPYY	1056	0	0
WRGG	WWR_	1419	0	0
WRGG	WPYY	1438	0	0
WRGW	WYG_	0	0	0
WRGW	WYG_	317	0	0
WRGW	WWGG	354	0	0
WRGW	Q	360	0	0
WRGW	WYG_	371	0	0
WRGW	WWWY	406	0	0
WRGW	WYYG	512	0	0
WRGW	WWRY	539	0	0
WRGW	WRYR	738	0	0
WRGW	WYG_	929	0	0
WRGW	WRYR	964	0	0
WRGW	WGRP	1142	0	0
WRGW	WGYY	1193	0	0
WRGW	WY_R	1196	0	0
WRGW	WYRW	1228	0	0
WRGW	WGRW	1258	0	0
WRGW	WW__(H)	1288	0	0
WRGW	WG__3	1296	0	0
WRGW	WRGG	1320	0	0
WRGW	WYRW	1378	0	0
WRGW	WGRP	1406	0	0
WRGW	WW__(H)	1413	0	0
WRWP	W_W_1	120	0	0
WRWP	WRGG	158	0	0
WRWP	W_W_1	162	0	0
WRWP	W_W_1	165	0	0
WRWP	WYY_	290	0	0
WRWP	WPGY	886	0	0
WRWP	WRGG	949	0	0
WRWP	WGWY	1110	0	0
WRWP	WGWW	1284	0	0
WRYR	Q	8	0	0
WRYR	WGG_	14	0	0
WRYR	WGG_	18	0	0
WRYR	WGRP	68	0	0
WRYR	W_W_1	560	0	0
WRYR	WW__(H)	707	0	0
WRYR	WW__(H)	709	0	0

WRYR	WRGW	742	0	0
WRYR	WRGW	959	0	0
WRYR	WRGW	964	0	0
WRYR	WW__2	1066	0	0
WRYR	W_W_1	1099	0	0
WRYR	WG__1	1132	0	0
WRYR	WYRW	1276	0	0
WRYR	WGYY	1293	0	0
WRYY	WYRW	0	0	0
WRYY	WPGG	431	0	0
WRYY	WPGG	758	0	0
WRYY	WY__	1002	0	0
WRYY	WPGG	1206	0	0
WRYY	WWR_	1212	0	0
WRYY	WG__1	1282	0	0
WRYY	WYWW	1329	0	0
WRYY	WG__1	1345	0	0
WRYY	_GGR	1348	0	0
WRYY	WRGG	1437	0	0
WRYY	WWR_	1438	0	0
WW__	____3	36	0	0
WW__	WG__1	325	0	0
WW__	_GGR	660	0	0
WW__	WGWW	825	0	0
WW__	W___1	906	0	0
WW__	WYY_	1090	0	0
WW__(H)	WW__(Z)	0	0	0
WW__(H)	WW__(Z)	622	0	0
WW__(H)	WW__(Z)	733	0	0
WW__(H)	_YGB	895	0	0
WW__(H)	_YGB	1316	0	0
WW__(Z)	WW__(H)	0	0	0
WW__(Z)	_YGW	583	0	0
WW__(Z)	WW__(H)	622	0	0
WW__(Z)	WRYR	708	0	0
WW__(Z)	WW__(H)	733	0	0
WW__(Z)	WRGW	775	0	0
WW__(Z)	WGRP	896	0	0
WW__(Z)	WGRP	918	0	0
WW__(Z)	WG__1	978	0	0
WW__(Z)	W_W_1	1012	0	0
WW__(Z)	W_YR	1035	0	0
WW__(Z)	WRYR	1085	0	0
WW__(Z)	WW__1	1194	0	0
WW__(Z)	WGRP	1336	0	0
WW__(Z)	WGGY	1352	0	0
WW__1	WPYY	19	0	0
WW__1	WYY_	34	0	0
WW__1	Q	38	0	0

WW__1	WWY_	45	0	0
WW__1	_YYY	48	0	0
WW__1	WYY_	54	0	0
WW__1	WYYG	121	0	0
WW__1	WG__1	135	0	0
WW__1	WG__1	235	0	0
WW__1	WYGR	288	0	0
WW__1	WYWW	321	0	0
WW__1	WYG_	405	0	0
WW__1	WYYB	422	0	0
WW__1	WG__1	492	0	0
WW__1	Q	553	0	0
WW__1	WWWY	592	0	0
WW__1	WWGY	646	0	0
WW__1	W_RG	681	0	0
WW__1	____3	702	0	0
WW__1	____3	754	0	0
WW__1	WG_Y	781	0	0
WW__1	WPPP	790	0	0
WW__1	WGGG	844	0	0
WW__1	WBGW	873	0	0
WW__1	WGGG	886	0	0
WW__1	WYY_	889	0	0
WW__1	WBGW	913	0	0
WW__1	WPYY	931	0	0
WW__1	_GGR	991	0	0
WW__1	_GGR	1034	0	0
WW__1	WGWY	1062	0	0
WW__1	_GGR	1112	0	0
WW__1	WGGW	1150	0	0
WW__1	WW__(Z)	1194	0	0
WW__2	WWGY	150	0	0
WW__2	W___1	845	0	0
WW__2	WYYG	862	0	0
WW__2	WWWY	894	0	0
WW__2	WGRP	912	0	0
WW__2	WGRP	971	0	0
WW__2	W_YY	993	0	0
WW__2	W_W_2	1058	0	0
WW__2	WBWY	1216	0	0
WW__2	_GGR	1248	0	0
WW__2	_GGR	1267	0	0
WW__2	WY__	1280	0	0
WW__2	_GGR	1311	0	0
WW__2	WPGG	1320	0	0
WW__2	WGG_	1332	0	0
WW__2	WBWY	1347	0	0
WW__2	W_W_1	1349	0	0
WW__2	WGRP	1360	0	0

WW__2	WGRP	1393	0	0
WW__2	WRGW	1418	0	0
WW__3	WPPP	0	0	0
WWGG	WWRY	11	0	0
WWGG	WGWW	36	0	0
WWGG	WY__	109	0	0
WWGG	WY__	305	0	0
WWGG	WG__3	306	0	0
WWGG	WRGW	332	0	0
WWGG	WRGW	347	0	0
WWGG	WW__	408	0	0
WWGG	WYG_	433	0	0
WWGG	WYYB	757	0	0
WWGG	WYG_	798	0	0
WWGG	WYG_	1325	0	0
WWGY	W_W_1	45	0	0
WWGY	WG__1	70	0	0
WWGY	WW__	153	0	0
WWGY	WG__1	348	0	0
WWGY	WGG_	359	0	0
WWGY	WRYR	390	0	0
WWGY	_GGR	613	0	0
WWGY	WY_G2	618	0	0
WWGY	W_W_1	624	0	0
WWGY	WW__	646	0	0
WWGY	WW__	658	0	0
WWGY	WGRP	792	0	0
WWGY	WG__1	828	0	0
WWGY	WG__1	1178	0	0
WWR_	WGWW	6	0	0
WWR_	WG__1	56	0	0
WWR_	WRGG	81	0	0
WWR_	WBWY	139	0	0
WWR_	WR__	216	0	0
WWR_	WBWY	227	0	0
WWR_	WYRW	233	0	0
WWR_	WWY_	541	0	0
WWR_	WY__	996	0	0
WWR_	_GGR	1087	0	0
WWR_	WG__1	1123	0	0
WWR_	W_W_1	1137	0	0
WWR_	_GGR	1232	0	0
WWR_	WRGG	1248	0	0
WWR_	WG__1	1269	0	0
WWR_	WRGG	1420	0	0
WWRY	WWY_	233	0	0
WWRY	WBWY	259	0	0
WWRY	W_W_2	1105	0	0
WWRY	WGG_	1198	0	0

WWWY	WGRP	124	0	0
WWWY	WYRR(DIE)	147	0	0
WWWY	WY_G2	243	0	0
WWWY	WGYY	458	0	0
WWWY	WWGY	602	0	0
WWWY	W___1	656	0	0
WWWY	_GGR	690	0	0
WWWY	WG_Y	781	0	0
WWWY	WGRP	807	0	0
WWWY	WG__1	831	0	0
WWWY	W___1	853	0	0
WWWY	WGGG	879	0	0
WWWY	WW__	897	0	0
WWWY	W___1	947	0	0
WWWY	W_W_1	1082	0	0
WWY_	WW__1	40	0	0
WWY_	WPYY	89	0	0
WWY_	Q	165	0	0
WWY_	WWR_	177	0	0
WWY_	WYRW	187	0	0
WWY_	WGRP	214	0	0
WWY_	WPGG	225	0	0
WWY_	WR__	610	0	0
WWY_	WRGG	648	0	0
WWY_	WG__1	666	0	0
WY__	_GGR	85	0	0
WY__	WWGG	109	0	0
WY__	WWGG	305	0	0
WY__	WRYY	316	0	0
WY__	WW__1	331	0	0
WY__	WYWW	335	0	0
WY__	Q	425	0	0
WY__	WWGG	770	0	0
WY__	_GGR	1067	0	0
WY__	WYWW	1108	0	0
WY__	WYY_	1146	0	0
WY__	WYY_	1240	0	0
WY__	WGRP	1248	0	0
WY__	__W_	1289	0	0
WY__	WG__1	1320	0	0
WY__	WRGG	1324	0	0
WY__	WRWP	1343	0	0
WY__	W___1	1353	0	0
WY__	WGYY	1359	0	0
WY__	WWGY	1363	0	0
WY_G1	WGG_	3	0	0
WY_G1	WG__2	20	0	0
WY_G1	WGRP	22	0	0
WY_G1	WGRP	25	0	0

WY_G1	WPGG	79	0	0
WY_G1	WGRP	93	0	0
WY_G1	WW__(H)	105	0	0
WY_G1	WYYG	133	0	0
WY_G1	WWGY	202	0	0
WY_G1	WWWY	245	0	0
WY_G1	WGG_	324	0	0
WY_G1	W_W_1	371	0	0
WY_G1	WGYY	394	0	0
WY_G1	WYYG	403	0	0
WY_G1	WRGW	455	0	0
WY_G1	WYYG	506	0	0
WY_G1	WWGY	666	0	0
WY_G1	_GGR	853	0	0
WY_G1	WPGG	893	0	0
WY_G1	WWGY	910	0	0
WY_G1	____2	914	0	0
WY_G1	WW__	918	0	0
WY_G1	WG__2	933	0	0
WY_G1	W_RG	969	0	0
WY_G1	W_W_1	970	0	0
WY_G1	WR__	977	0	0
WY_G1	W_W_1	984	0	0
WY_G1	WWGY	1000	0	0
WY_G1	WW__2	1033	0	0
WY_G1	____2	1062	0	0
WY_G1	WGG_	1097	0	0
WY_G1	____2	1123	0	0
WY_G1	WW__2	1145	0	0
WY_G2	WGG_	6	0	0
WY_G2	WGRP	21	0	0
WY_G2	WGRP	24	0	0
WY_G2	WPGG	82	0	0
WY_G2	WPGG	86	0	0
WY_G2	WGRP	92	0	0
WY_G2	WW__(Z)	114	0	0
WY_G2	WGRP	126	0	0
WY_G2	WWGY	201	0	0
WY_G2	WWWY	207	0	0
WY_G2	WWWY	229	0	0
WY_G2	WGRP	368	0	0
WY_G2	WGRP	372	0	0
WY_G2	WGWY	384	0	0
WY_G2	WRGW	399	0	0
WY_G2	WYYG	400	0	0
WY_G2	WRGW	450	0	0
WY_G2	WYYG	506	0	0
WY_G2	WRGW	543	0	0
WY_G2	WGWY	559	0	0

WY_G2	WWGY	663	0	0
WY_G2	WWRY	695	0	0
WY_G2	_GGR	859	0	0
WY_G2	_GGR	883	0	0
WY_G2	_GGR	888	0	0
WY_G2	_GGR	902	0	0
WY_G2	WPGG	903	0	0
WY_G2	WWWY	905	0	0
WY_G2	WGYY	930	0	0
WY_G2	PYPG	957	0	0
WY_G2	W_RG	969	0	0
WY_G2	W_W_1	971	0	0
WY_G2	WWGY	1005	0	0
WY_G2	WW__2	1031	0	0
WY_G2	WWWY	1116	0	0
WY_G2	____2	1124	0	0
WY_G2	____2	1126	0	0
WY_G2	Q	1259	0	0
WY_G2	Q	1370	0	0
WY_R	WR__	0	0	0
WY_R	WYY_	6	0	0
WY_R	WGWW	23	0	0
WY_R	WYY_	74	0	0
WY_R	_GGR	132	0	0
WY_R	WR__	159	0	0
WY_R	WPYY	226	0	0
WY_R	W___1	248	0	0
WY_R	WPYY	284	0	0
WY_R	WBGW	938	0	0
WY_R	WPYY	1210	0	0
WY_R	WGWW	1388	0	0
WY_R	_YYY	1392	0	0
WY_R	WBGW	1405	0	0
WYG_	WRGW	371	0	0
WYG_	WWGG	433	0	0
WYG_	WYYB	457	0	0
WYG_	WYYB	1011	0	0
WYG_	WYYB	1019	0	0
WYGR	WYWW	3	0	0
WYGR	WYRW	66	0	0
WYGR	WWY_	272	0	0
WYGR	WW__1	285	0	0
WYGR	WRYY	774	0	0
WYRW	WRYY	0	0	0
WYRW	WYGR	101	0	0
WYRW	WWY_	187	0	0
WYRW	WPYY	232	0	0
WYRW	WPYY	380	0	0
WYRW	WY__	449	0	0

WYRW	W_RG	459	0	0
WYRW	WPGY	477	0	0
WYRW	W_W_1	678	0	0
WYRW	WPGR	696	0	0
WYRW	WGGG	713	0	0
WYRW	_GGR	740	0	0
WYRW	WPGR	810	0	0
WYRW	W_BG	848	0	0
WYRW	W_RG	852	0	0
WYRW	WWGY	869	0	0
WYRW	WW__2	875	0	0
WYRW	WWGY	887	0	0
WYRW	____1	950	0	0
WYRW	W_YY	962	0	0
WYRW	WG__1	984	0	0
WYRW	WW__(Z)	1045	0	0
WYRW	W(G)Y_	1089	0	0
WYRW	WWRY	1107	0	0
WYRW	WWRY	1131	0	0
WYRW	W_YR	1153	0	0
WYRW	W_YR	1256	0	0
WYWW	WGG_	0	0	0
WYWW	WGRP	230	0	0
WYWW	WGRP	305	0	0
WYWW	WY__	335	0	0
WYWW	WPYY	342	0	0
WYWW	WGRP	359	0	0
WYWW	WGRP	1091	0	0
WYWW	WY__	1112	0	0
WYWW	WRGW	1211	0	0
WYWW	WW__1	1244	0	0
WYWW	WYGR	1258	0	0
WYWW	WR__	1325	0	0
WYWW	WGRP	1346	0	0
WYWW	WGRP	1362	0	0
WYY_	WRGG	0	0	0
WYY_	WW__1	55	0	0
WYY_	WPYY	61	0	0
WYY_	WY_R	62	0	0
WYY_	Q	67	0	0
WYY_	WY_R	72	0	0
WYY_	W_YY	96	0	0
WYY_	_YYY	102	0	0
WYY_	WR__	110	0	0
WYY_	WPGR	152	0	0
WYY_	WRWP	167	0	0
WYY_	WGWW	205	0	0
WYY_	WRWP	246	0	0
WYY_	WR__	252	0	0

WYY_	WR__	259	0	0
WYY_	WY_R	260	0	0
WYY_	WYRW	265	0	0
WYY_	WRWP	289	0	0
WYY_	WW__1	892	0	0
WYY_	WGWY	910	0	0
WYY_	WGGG	936	0	0
WYY_	WGGG	943	0	0
WYY_	WGGG	970	0	0
WYY_	WGGG	994	0	0
WYY_	WY_R	1072	0	0
WYY_	WW__	1090	0	0
WYY_	_YGW	1170	0	0
WYY_	PYPG	1197	0	0
WYY_	WPYY	1220	0	0
WYY_	Q	1231	0	0
WYY_	WWR_	1243	0	0
WYY_	WGWW	1254	0	0
WYY_	WWY_	1270	0	0
WYY_	WYGR	1277	0	0
WYY_	WYWW	1311	0	0
WYY_	WG__1	1338	0	0
WYY_	WG__1	1356	0	0
WYY_	WRGG	1393	0	0
WYY_	WG__1	1407	0	0
WYY_	WYRR(DIE)	1423	0	0
WYYB	WY_G2	30	0	0
WYYB	WY_G2	68	0	0
WYYB	WWRY	189	0	0
WYYB	WRGW	383	0	0
WYYB	WW__(H)	471	0	0
WYYB	WBWY	891	0	0
WYYB	DIE	1081	0	0
WYYB	DIE	1223	0	0
WYYG	WW__	110	0	0
WYYG	WW__	121	0	0
WYYG	WGRP	135	0	0
WYYG	____2	395	0	0
WYYG	WY_G1	427	0	0
WYYG	WWRY	758	0	0
WYYG	WGRP	1207	0	0
WYYG	WYG_	1230	0	0
WYYG	_GGR	1253	0	0
WYYG	WYG_	1320	0	0
WYYG	Q	1395	0	0

Antlist	InitialPosX	InitialPosY	ColonyArea	AntLength	MinX	MinY	MaxX	MaxY
_YGW	NA	NA	349574	56.3	67	0	723	576
WYYB	NA	NA						
____1	NA	NA						
DIE	NA	NA						
WRGW	NA	NA						
_GGR	NA	NA						
WYG_	NA	NA						
WWRY	NA	NA						
WW__(H)	NA	NA						
WW__(Z)	NA	NA						
WWGG	NA	NA						
WBWY	NA	NA						
WGWY	NA	NA						
WGRP	NA	NA						
WYWW	NA	NA						
WY__	NA	NA						
WYGR	NA	NA						
WG___1	NA	NA						
WGWW	NA	NA						
WWR_	NA	NA						
WYRW	NA	NA						
WRYY	NA	NA						
WW__1	NA	NA						
WPYY	NA	NA						
WRGG	NA	NA						
WYY_	NA	NA						
W_YR	NA	NA						
W_W_1	NA	NA						
WWY_	NA	NA						
WPGR	NA	NA						
WPGY	NA	NA						
WRWP	NA	NA						
_YYY	NA	NA						
WR__	NA	NA						
WY_R	NA	NA						
WGGW	NA	NA						
W_YY	NA	NA						
W_BG	NA	NA						
WW__2	NA	NA						
WGGG	NA	NA						
W___1	NA	NA						
W_RG	NA	NA						
WGGY	NA	NA						
WGG_	NA	NA						
WG_Y	NA	NA						
WG__1	NA	NA						
W___2	NA	NA						
____2	NA	NA						
PYPG	NA	NA						
WYRR(DIE)	NA	NA						
WW__	NA	NA						
____3	NA	NA						
WPPP	NA	NA						
WW__3	NA	NA						
WWGY	NA	NA						
WG__2	NA	NA						
WYYG	NA	NA						
WY_G1	NA	NA						
WWWY	NA	NA						
WRYR	NA	NA						
WPGG	NA	NA						
W_W_2	NA	NA						
Q	NA	NA						
WY_G2	NA	NA						
WGYY	NA	NA						
WGRW	NA	NA						
WG__3	NA	NA						
_YGB	NA	NA						
__W_	NA	NA						
W(G)Y_	NA	NA						
WBGW	NA	NA						
WGWW2	NA	NA						
___W	NA	NA						

Actor	Target	ActorPosX	ActorPosY	Time
WWR_	WG__	255	359	584
WWR_	ZZZZ	238	377	755
WYYG	W_GY	356	272	1630
WYYG	WGGW	311	348	1677
AAAA	W_GY	319	244	253
AAAA	WYYG	292	297	265
AAAA	WYYG	285	299	271
AAAA	W_W_	290	315	275
AAAA	WYYG	288	292	276
AAAA	W_W_	293	298	277
AAAA	WYYG	291	293	310
AAAA	W_W_	298	299	314
AAAA	WYYG	285	290	326
AAAA	WWGY	294	276	340
AAAA	_R__	303	108	377
AAAA	WBGW	273	95	385
AAAA	_R__	325	111	485
AAAA	WYYB	304	144	479
AAAA	WBYY	321	75	491
AAAA	WBGW	302	83	493
AAAA	WBYY	318	76	501
AAAA	WWGY	337	85	510
AAAA	WYRW	359	107	527
AAAA	WYG_	410	164	562
AAAA	XXXX	488	288	693
AAAA	WY_G	518	395	768
AAAA	WYY_	564	443	818
AAAA	WY_G	513	403	841
AAAA	JJJJ	495	264	1405
AAAA	WYG_	469	254	1410
AAAA	WRWB	488	176	1660
AAAA	JJJJ	486	232	1649
W_W_	WWR_	295	355	210
W_W_	WWR_	275	347	235
W_W_	AAAA	303	307	267
W_W_	WRWB	401	202	549
W_W_	WYRW	357	166	596
W_W_	WBGG	311	204	623
W_W_	W_GY	332	280	668
W_W_	WBGG	339	260	1083
W_W_	W_GY	319	291	1094
W_W_	WYYG	314	260	277
W_W_	WYYG	300	291	1773
W_WG	W_W_	244	207	127
W_WG	WBGG	264	175	141
W_WG	AAAA	264	235	322
W_WG	W_W_	306	291	352
W_WG	WWR_	282	357	376

W_WG	WWR_	270	403	845
W_WG	WWR_	236	353	1411
WBGG	WGYW	350	127	867
WBGG	W_RG	367	206	990
WBGG	WYYB	335	203	1043
WBGG	W_RG	370	206	1049
WBGG	WWGY	275	226	1125
WBGG	WYG_	406	203	1200
WBGG	WGG_	531	203	1233
WBGG	XXXX	465	270	1256
WBGG	W_GY	386	261	1306
WBGG	WY_G	392	249	1309
WBGG	W_GY	387	249	1312
WBGG	W_GY	352	220	1529
WYRW	WYYB	329	166	105
WYRW	WGYW	384	122	120
WYRW	HHHH	375	113	125
WYRW	_R__	346	113	356
WYRW	_R__	346	113	358
WYRW	WG__	376	138	414
WYRW	WYG_	390	146	427
WYRW	WGYW	375	112	523
WYRW	WWGY	379	127	576
WYRW	WYYB	290	154	614
WYRW	WBGG	290	154	616
WYRW	WBGW	290	139	629
WYRW	WBGW	291	107	736
WYRW	_R__	313	124	779
WYRW	WYYB	258	152	850
WYRW	WYYB	236	185	906
WYRW	W_RG	354	183	931
WYRW	WW__	360	246	966
WYRW	WGGW	385	383	1072
WYRW	WGGY	398	438	1089
WYRW	WG__	367	440	1091
WYRW	WG__	348	451	1089
WYRW	WGGY	376	467	1112
WYRW	WGGG	472	476	1142
WYRW	WWY_	383	431	1270
WYRW	W_GY	343	239	1288
WYRW	WGYW	361	215	1289
WYRW	WY_G	361	215	1293
WYRW	W_RG	369	189	1300
WYRW	WGRB	389	130	1502
WYRW	WW__	440	62	1527
WYRW	WRGG	504	92	1553
WYRW	_Y_B	468	79	1567
WYRW	WGG_	410	152	1632
WYRW	_Y_B	454	78	1648

WYRW	WGYW	445	79	1654
WYRW	_Y_B	452	69	1734
WYRW	WBWY	439	76	1750
WYRW	_Y_B	457	84	1730
WYRW	WW__	457	71	1758
WYRW	WYG_	500	59	1764
WYRW	WGRB	505	73	1766
WYRW	WYWY	555	97	1771
WYRW	_YYY	567	62	1775
WYYB	WBGG	281	159	131
WYYB	WBGW	284	134	138
WYYB	WBGG	306	157	501
WYYB	WBGG	283	164	636
WYYB	WYRW	283	164	639
WYYB	WYRW	286	160	825
WYYB	WBGG	276	172	831
WYYB	WGYW	274	162	991
WYYB	W_RG	363	190	1040
WYYB	WYRW	312	131	1064
WYYB	WWGY	290	112	1555
WYYB	W_W_	295	240	1755
WBGW	WYYR	255	86	242
WBGW	WYRW	307	124	309
WBGW	WBGG	307	132	312
WBGW	WYYB	294	135	314
WBGW	EEEE	281	93	335
WBGW	FFFF	293	88	336
WBGW	WYRW	279	78	339
WBGW	_R__	304	125	619
WBGW	_R__	313	108	649
WBGW	WGYW	363	111	943
WBGW	WBYY	339	77	970
WBGW	WYYR	341	88	973
WBGW	WBYY	329	69	976
WBGW	WYYR	295	96	1006
WBGW	WGYW	268	99	1192
WBGW	WWGY	247	70	1504
WBGW	WWGY	247	70	1509
WBGW	WBYY	247	108	1758
WYYR	_R__	239	126	128
WYYR	W_WG	246	159	138
WYYR	_R__	233	126	149
WYYR	W_W_	248	204	167
WYYR	EEEE	264	77	845
WYYR	WBGW	299	89	853
WYYR	FFFF	299	89	866
WYYR	_R__	301	78	888
WYYR	_R__	314	72	926
WYYR	WBYY	362	66	1580

WYYR	_R__	362	87	1627
EEEE	WBGW	245	70	336
WWY_	W_GY	353	396	190
WWY_	W_GY	328	427	212
WWY_	W_W_	297	374	223
WWY_	W_W_	313	353	225
WWY_	W_GY	331	366	233
WWY_	WGGY	402	406	404
WWY_	WGGY	376	420	410
WWY_	W_WG	322	416	761
WWY_	WGGW	353	384	853
WWY_	WW__	327	356	1016
WWY_	WYRW	372	338	1034
WWY_	WBGG	357	253	1049
WWY_	PPPP	514	253	1070
WWY_	JJJJ	492	209	1080
WWY_	WYG_	445	244	1158
WWY_	XXXX	462	264	1159
WWY_	WYG_	431	258	1164
WWY_	WG__	484	209	1190
WWY_	XXXX	480	262	1208
WWY_	WYG_	439	245	1228
WWY_	XXXX	452	271	1230
WWY_	WGGW	348	375	1257
WWY_	WGGW	374	337	1305
WWY_	WYG_	422	276	1314
WWY_	XXXX	483	203	1321
WWY_	WRWB	505	190	1323
WWY_	WGG_	436	179	1351
WWY_	WYG_	467	236	1361
WWY_	PPPP	481	235	1364
WWY_	WRWB	503	206	1365
WWY_	MMMM	439	400	1378
WWY_	AAAA	509	400	1380
WWY_	WYY_	553	481	1394
WWY_	WGGW	435	337	1496
WWY_	W_GY	341	251	1679
_R__	FFFF	329	105	58
_R__	WYRW	306	134	72
_R__	WYRW	306	96	222
_R__	WYRW	322	91	338
_R__	WYYR	324	89	888
_R__	WWGY	390	86	1602
_R__	AAAA	390	99	1749
W_GY	WWY_	371	405	133
W_GY	WGGY	384	416	166
W_GY	WGG_	414	389	178
W_GY	WWY_	377	407	191
W_GY	WGGY	374	425	200

W_GY	WWY_	347	414	212
W_GY	WYYG	277	240	252
W_GY	WBGG	309	211	263
W_GY	WYYB	294	173	270
W_GY	WWGY	319	279	344
W_GY	WGGW	357	301	362
W_GY	WGGY	413	409	453
W_GY	WGGY	422	436	489
W_GY	W_W_	347	264	522
W_GY	AAAA	333	259	549
W_GY	WYYG	281	304	641
WGGY	WGG_	442	471	615
WGGY	WGG_	449	471	626
WGGY	W_WG	378	479	692
WGGY	WG__	395	479	935
WGGY	WWR_	247	421	1350
WGG_	XXXX	517	347	291
WGG_	WY_G	504	304	294
WGG_	XXXX	517	337	296
WGG_	MMMM	524	417	307
WGG_	WYBG	539	431	310
WGG_	UUUU	579	414	339
WGG_	WYBG	527	343	360
WGG_	PPPP	505	273	367
WGG_	_YYY	545	179	402
WGG_	WYBG	550	179	501
WGG_	XXXX	528	261	514
WGG_	OOOO	543	268	537
WGG_	OOOO	548	268	557
WGG_	W__W	535	240	572
WGG_	W_W_	364	270	733
WGG_	WGGW	396	369	861
WGG_	JJJJ	446	337	879
WGG_	WYG_	451	337	895
WGG_	JJJJ	463	336	904
WGG_	WYG_	418	261	1141
WGG_	WYG_	398	243	1168
WGG_	WWY_	439	266	1184
WGG_	OOOO	544	262	1207
WGG_	WRWB	544	201	1239
WGG_	WY__	513	144	1247
WGG_	WW__	492	140	1248
WGG_	XXXX	509	153	1277
WGG_	W_RG	417	167	1295
WBGR	MMMM	501	450	13
WBGR	XXXX	522	431	20
WBGR	WYBG	545	466	33
WBGR	WYBG	545	460	47
WBGR	WGGG	497	465	114

WBGR	XXXX	502	453	141
WBGR	WYBG	522	471	148
WBGR	WGGG	492	466	202
WBGR	XXXX	508	455	216
WBGR	WYBG	547	455	237
WBGR	WYY_	565	458	250
WBGR	UUUU	578	434	292
WBGR	WYY_	615	483	303
WBGR	WYBG	570	466	323
WBGR	W___	695	375	491
WBGR	WBWY	686	368	628
WGGG	WBGR	473	463	28
WGGG	IIII	463	456	1167
WGGG	IIII	463	456	1393
WGGW	AAAA	279	305	1
WGGW	WG__	406	290	124
WGGW	JJJJ	406	292	127
WGGW	WG__	411	278	131
WGGW	JJJJ	421	278	137
WGGW	WG__	422	469	139
WGGW	W_GY	372	312	361
WGGW	W_W_	318	297	466
WGGW	WRWB	266	233	701
WGGW	W_W_	319	258	711
WGGW	W_W_	308	275	717
WGGW	WRWB	306	273	720
WGGW	W_GY	303	277	721
WGGW	WRWB	295	265	728
WGGW	WYYG	279	280	731
WGGW	WWR_	257	352	750
JJJJ	WRWB	441	270	517
JJJJ	WGG_	455	340	899
JJJJ	WYG_	461	338	901
JJJJ	WY_G	504	366	909
JJJJ	WYG_	519	373	914
JJJJ	AAAA	533	377	917
JJJJ	WYG_	532	365	922
JJJJ	OOOO	527	291	974
JJJJ	WGG_	500	291	980
JJJJ	WRWB	527	183	1012
JJJJ	WWY_	517	186	1081
JJJJ	WRWB	534	186	1153
JJJJ	WGRB	525	188	1156
JJJJ	WG_W	531	188	1158
JJJJ	WRWB	542	186	1174
JJJJ	WG_W	535	186	1176
WG__	W_W_	305	248	145
WG__	AAAA	305	283	155
WG__	AAAA	299	282	162

WG__	WRWB	463	250	197
WG__	W_BG	491	213	208
WG__	_YYY	511	208	211
WG__	WY__	485	181	230
WG__	WRGG	523	293	239
WG__	MMMM	477	208	261
WG__	WYG_	477	194	263
WG__	WRWB	468	233	268
WG__	W_GY	409	288	297
WG__	WRWB	441	267	302
WG__	W_BG	463	240	324
WG__	WYRW	383	140	407
WG__	WWGY	378	156	409
WG__	WBGG	342	184	428
WG__	WWR_	289	354	450
WG__	WGGG	46	482	623
WG__	WGGY	348	458	994
WG__	WYRW	330	461	1111
WG__	WGGY	272	461	1281
WG__	WGGG	452	474	1395
WG__	WYY_	564	487	1505
WG__	W_GW	607	487	1511
WG__	WW__	623	480	1543
WG__	W_GW	623	480	1546
WG__	WW__	623	471	1603
WG__	WYY_	603	463	1608
WWGY	W__W	382	197	156
WWGY	WGYW	374	164	169
WWGY	WYRW	369	137	175
WWGY	WGYW	373	137	175
WWGY	WBGG	358	159	178
WWGY	WGYW	373	148	180
WWGY	WYRW	373	136	181
WWGY	HHHH	378	136	182
WWGY	WGYW	378	140	182
WWGY	AAAA	290	212	201
WWGY	W_WG	237	224	212
WWGY	WYYG	258	267	288
WWGY	W_WG	316	240	327
WWGY	WBGG	307	203	361
WWGY	WG__	376	200	372
WWGY	WYG_	400	186	432
WWGY	WGWY	411	130	461
WWGY	WGRB	418	109	473
WWGY	HHHH	385	88	492
WWGY	WBYY	378	65	506
WWGY	_R__	373	71	507
WWGY	HHHH	371	71	508
WWGY	WBYY	360	71	510

WWGY	_R__	355	71	512
WWGY	WBYY	346	71	550
WWGY	_R__	357	78	552
WWGY	WYRW	365	88	553
WWGY	AAAA	356	94	555
WWGY	WYRW	362	94	556
WWGY	WYRW	362	105	569
WWGY	WYRW	379	116	606
WWGY	WGRB	400	146	810
WWGY	WRWB	408	176	847
WWGY	WGWY	377	125	854
WWGY	WRWB	404	130	862
WWGY	WGRB	419	123	865
WWGY	WGWY	382	116	869
WWGY	_YYY	517	163	920
WWGY	WW_W	494	137	921
WWGY	WGRB	502	122	921
WWGY	LLLL	499	95	922
WWGY	WYWY	507	88	924
WWGY	WGRB	516	96	927
WWGY	WY__	506	115	939
WWGY	_YYY	516	111	940
WWGY	_YYY	516	116	964
WWGY	WY__	510	122	966
WWGY	_YYY	520	127	970
WWGY	WY__	525	133	970
WWGY	WRWB	526	198	984
WWGY	WYWY	542	115	1033
WWGY	WRWB	533	164	1051
WWGY	LLLL	481	26	1057
WWGY	HHHH	382	94	1065
WWGY	FFFF	370	112	1066
WWGY	WBGW	300	98	1069
WWGY	WGYW	277	103	1075
WWGY	WYYB	294	117	1082
WWGY	AAAA	288	143	1096
WWGY	WBGG	294	178	1099
WWGY	WYYG	260	240	1125
WWGY	W_WG	246	303	1228
WWGY	WYYB	246	191	1281
WWGY	WYYR	303	86	1319
WWGY	WYYR	316	89	1345
WWGY	WBGW	275	89	1357
WWGY	WBGW	244	98	1382
WWGY	WBYY	305	90	1536
WWGY	HHHH	305	90	1536
WWGY	EEEE	300	78	1540
WWGY	WYYR	343	87	1562
WWGY	WBYY	306	87	1571

WWGY	HHHH	318	94	1579
WWGY	WYYB	318	105	1581
WWGY	WBYY	322	76	1586
WWGY	HHHH	321	98	1587
WWGY	WW__	427	47	1595
WWGY	_YYY	434	71	1606
WWGY	WGRB	436	77	1608
WWGY	_YYY	436	71	1609
WWGY	WYRW	436	121	1612
WWGY	WGG_	440	160	1616
WWGY	WYG_	459	190	1620
WWGY	WRWB	490	172	1630
WWGY	W_BG	518	186	1633
WWGY	W__W	557	183	1636
WWGY	W___	646	160	1641
WWGY	WRR_	675	90	1783
WWGY	WWRY	676	80	1784
HHHH	WW__	364	85	71
HHHH	WYRW	362	90	170
HHHH	WYRW	357	98	239
HHHH	WYRW	357	98	242
HHHH	WYRW	364	98	357
HHHH	_R__	361	98	364
HHHH	XXXX	361	95	376
HHHH	WWGY	363	94	510
HHHH	_R__	355	89	517
HHHH	WWGY	364	87	519
HHHH	WGYW	389	80	527
HHHH	WYRW	387	83	529
HHHH	WGYW	407	80	533
HHHH	FFFF	417	74	539
HHHH	WGYW	413	71	547
HHHH	FFFF	413	62	553
HHHH	FFFF	415	49	804
HHHH	_YYY	423	49	810
HHHH	_YYY	427	49	820
HHHH	_YYY	423	49	865
HHHH	_YYY	448	49	912
HHHH	WG_W	463	49	920
HHHH	WG_W	479	32	927
HHHH	WW__	478	32	935
HHHH	WG_W	478	47	940
HHHH	WW__	486	30	941
HHHH	WBYY	371	55	952
HHHH	WBYY	362	51	966
HHHH	_R__	356	90	1004
HHHH	WBYY	353	62	1045
HHHH	EEEE	324	48	1143
HHHH	WBYY	317	54	1146

HHHH	EEEE	301	45	1354
HHHH	WBYY	301	80	1377
HHHH	EEEE	307	71	1382
HHHH	WBYY	330	78	1403
HHHH	WBYY	345	83	1408
HHHH	WBYY	341	83	1465
HHHH	WBYY	331	83	1517
HHHH	WGRB	346	93	1571
HHHH	_R__	358	85	1574
HHHH	WWGY	355	98	1590
HHHH	WGRB	355	98	1591
HHHH	WYYR	358	113	1606
HHHH	_R__	364	122	1660
HHHH	WGG_	391	153	1694
HHHH	WYRW	396	146	1697
HHHH	WYG_	420	158	1701
HHHH	WG_W	488	164	1708
HHHH	W___	515	161	1713
HHHH	W_BG	523	174	1726
HHHH	WYBG	531	154	1732
HHHH	W_BG	535	178	1735
HHHH	JJJJ	529	203	1738
HHHH	W___	492	162	1747
HHHH	W___	498	152	1755
HHHH	W___	513	167	1772
HHHH	WGRB	499	118	1784
WGYW	WGRB	419	137	11
WGYW	WYG_	419	142	72
WGYW	HHHH	390	125	192
WGYW	WYG_	419	148	197
WGYW	WW_W	488	156	207
WGYW	WGRB	430	129	211
WGYW	WW_W	466	141	215
WGYW	WY__	468	145	216
WGYW	WGRB	402	124	223
WGYW	WW_W	456	137	228
WGYW	WY__	470	144	230
WGYW	WGRB	484	140	233
WGYW	_R__	480	132	235
WGYW	WYG_	453	160	240
WGYW	WYG_	400	164	252
WGYW	WW_W	454	141	256
WGYW	WYG_	471	157	257
WGYW	WY__	471	157	257
WGYW	WW_W	471	145	264
WGYW	WY__	471	145	265
WGYW	W_RG	474	147	267
WGYW	WYG_	464	162	272
WGYW	WGRB	445	133	300

WGYW	WGRB	442	122	306
WGYW	WW_W	442	122	306
WGYW	WGRB	430	131	345
WGYW	WGRB	438	79	358
WGYW	HHHH	393	84	367
WGYW	WG__	427	134	389
WGYW	WY__	450	151	407
WGYW	WW_W	455	139	411
WGYW	WGRB	446	120	447
WGYW	HHHH	400	97	522
WGYW	WWGY	400	85	524
WGYW	WBYY	381	55	546
WGYW	WWGY	381	59	547
WGYW	WGRB	436	79	563
WGYW	_YYY	436	76	805
WGYW	_R__	372	103	821
WGYW	WBYY	361	81	891
WGYW	WBYY	357	77	904
WGYW	_R__	357	77	904
WGYW	WBGW	361	92	928
WGYW	W_RG	390	146	935
WGYW	WBGW	352	128	950
WGYW	WBGG	291	166	968
WGYW	WYYB	266	166	971
WGYW	WBGG	289	166	974
WGYW	WBGW	272	112	1090
WGYW	WBYY	299	96	1107
WGYW	JJJJ	287	102	1136
WGYW	JJJJ	294	115	1177
WGYW	WBGW	308	108	1178
WGYW	WBYY	308	93	1195
WGYW	WBGW	312	110	1202
WGYW	_R__	319	79	1224
WGYW	HHHH	318	78	1229
WGYW	HHHH	309	72	1249
WGYW	_R__	315	92	1320
WGYW	WBYY	315	85	1322
WGYW	_R__	321	85	1322
WGYW	WBYY	316	82	1326
WGYW	HHHH	334	60	1339
WGYW	WGRB	425	83	1347
WGYW	WW__	444	69	1362
WGYW	WYWY	539	84	1397
WGYW	W_W_	544	87	1434
WGYW	WRGG	531	59	1526
WGYW	WW__	476	73	1534
WGYW	WYRW	476	73	1534
WGYW	LLLL	492	69	1542
WGYW	_Y_B	492	82	1563

WGYW	WYRW	486	82	1566
WGYW	_Y_B	486	78	1569
WGYW	LLLL	522	56	1673
WGYW	_Y_B	506	62	1679
WGYW	WRGG	497	62	1687
WGYW	_Y_B	478	64	1708
WGYW	_R__	420	101	1751
WGYW	WW__	455	70	1757
WGYW	WYWY	516	51	1774
W__W	WG__	421	235	4
W__W	WRWB	427	238	222
W__W	W_BG	466	235	244
W__W	WYG_	454	211	264
W__W	XXXX	454	170	511
W__W	W_BG	500	204	556
W__W	PPPP	547	209	564
W__W	PPPP	559	227	750
W__W	WYBG	557	204	815
W__W	WYBG	570	196	837
W__W	WGRB	612	178	955
W__W	WRWB	590	171	961
W__W	WY_R	590	177	1214
W__W	WRWB	582	156	1218
W__W	WYBG	592	129	1221
W__W	_YYY	582	133	1225
W__W	WYBG	603	115	1424
W__W	QQQQ	603	115	1424
W__W	WGWY	593	127	1427
W__W	WYBG	598	122	1444
W__W	WRR_	608	115	1447
W__W	WYBG	611	120	1454
W__W	WGWY	619	146	1500
W__W	WYBG	601	146	1503
W__W	WRR_	618	109	1523
W__W	QQQQ	604	116	1527
W__W	WYBG	575	161	1587
W__W	WY__	579	171	1592
W__W	WRWB	572	164	1614
WW__	HHHH	369	66	32
WW__	HHHH	365	63	65
WW__	HHHH	377	63	74
WW__	WG_W	463	50	84
WW__	WRGG	466	45	106
WW__	WG_W	476	24	111
WW__	WG_W	471	46	133
WW__	WG_W	450	51	136
WW__	WG_W	455	50	204
WW__	LLLL	477	23	219
WW__	_Y_B	480	8	795

WW__	WGRB	476	82	803
WW__	WW_W	470	82	810
WW__	_Y_B	473	77	811
WW__	WW_W	467	84	814
WW__	WG_W	492	69	816
WW__	LLLL	495	49	817
WW__	WG_W	498	55	820
WW__	_Y_B	481	53	821
WW__	FFFF	478	59	822
WW__	_YYY	518	164	829
WW__	W__W	519	182	835
WW__	W_BG	501	168	835
WW__	WRWB	473	166	838
WW__	WYG_	464	175	842
WW__	WRWB	446	185	848
WW__	WYG_	470	203	853
WW__	W_RG	464	193	856
WW__	WYG_	472	197	858
WW__	XXXX	508	228	860
WW__	XXXX	528	245	866
WW__	OOOO	543	260	872
WW__	XXXX	536	258	879
WW__	WYG_	499	275	884
WW__	OOOO	536	268	886
WW__	WRWB	521	231	901
WW__	W_W_	352	254	958
WW__	WYRW	373	321	997
WW__	W_W_	344	289	1002
WW__	WWY_	338	331	1013
WW__	W_W_	295	313	1056
WW__	W_W_	343	269	1061
WW__	WBGG	341	257	1063
WW__	ZZZZ	315	239	1076
WW__	WYYB	297	169	1082
WW__	WGYW	297	169	1082
WW__	WWGY	296	154	1084
WW__	WGWY	262	130	1093
WW__	WBYY	299	85	1099
WW__	EEEE	305	68	1100
WW__	HHHH	366	50	1105
WW__	FFFF	481	28	1122
WW__	LLLL	478	30	1323
WW__	LLLL	481	41	1364
WW__	FFFF	480	21	1416
WW__	WRGG	480	40	1425
WW__	LLLL	478	44	1493
WW__	FFFF	482	38	1494
WW__	_Y_B	482	19	1557
WW__	_Y_B	448	53	1565

WW__	_Y_B	431	52	1595
WW__	_Y_B	431	56	1612
WW__	WBWY	388	55	1667
WW__	WYYR	365	68	1681
WW__	WBYY	350	82	1686
WW__	WBYY	291	119	1698
WW__	WYYB	281	157	1725
FFFF	WBYY	325	70	50
FFFF	WBYY	323	70	55
FFFF	WBYY	326	70	60
FFFF	_R__	327	86	69
FFFF	HHHH	328	74	110
FFFF	WBYY	338	72	111
FFFF	HHHH	345	77	113
FFFF	WBYY	346	72	114
FFFF	HHHH	341	75	118
FFFF	WBYY	340	67	131
FFFF	WBYY	338	59	217
FFFF	WYRW	333	83	221
FFFF	WBYY	337	70	224
FFFF	WYRW	341	70	225
FFFF	WBYY	330	65	227
FFFF	_R__	301	65	268
FFFF	WBYY	311	54	270
FFFF	WBGW	305	64	273
FFFF	WBYY	313	57	275
FFFF	_R__	314	69	280
FFFF	HHHH	345	74	281
FFFF	WW__	466	54	291
FFFF	WG_W	466	54	291
FFFF	LLLL	493	59	294
FFFF	LLLL	528	52	314
FFFF	LLLL	477	57	327
FFFF	WBYY	377	68	336
FFFF	LLLL	475	49	489
FFFF	WW_W	465	80	578
FFFF	LLLL	476	57	591
FFFF	_Y_B	470	49	799
FFFF	WGRB	432	81	808
FFFF	_Y_B	462	75	816
FFFF	WYWY	497	87	819
FFFF	_Y_B	13	44	824
FFFF	WW_W	14	1	841
FFFF	HHHH	427	59	843
FFFF	WGWY	398	69	847
FFFF	_R__	359	89	852
FFFF	WYYR	334	89	854
FFFF	_R__	331	89	855
FFFF	_R__	331	94	871

FFFF	WGRB	436	71	880
FFFF	LLLL	492	49	885
FFFF	WG_W	503	46	940
FFFF	WYWY	537	63	946
FFFF	WG_W	522	75	948
FFFF	WWGY	513	89	951
FFFF	_YYY	525	92	953
FFFF	WWGY	522	96	954
FFFF	WYWY	522	96	969
FFFF	LLLL	483	47	982
FFFF	_Y_B	503	30	1559
FFFF	WRGG	529	63	1561
FFFF	WYG_	497	13	1749
FFFF	_Y_B	495	20	1754
FFFF	WBWY	485	33	1757
FFFF	_Y_B	551	53	1777
FFFF	_Y_B	601	230	1787
FFFF	_Y_B	546	394	1793
WBYY	WW__	379	67	74
WBYY	FFFF	363	48	116
WBYY	XXXX	361	49	372
WBYY	WGYW	345	65	845
WBYY	HHHH	348	59	846
WBYY	WGWY	351	65	894
WBYY	WBGW	340	80	974
WBYY	HHHH	344	59	975
WBYY	WBGW	344	62	978
WBYY	HHHH	352	57	1091
WBYY	WW__	318	80	1101
WBYY	WYYR	317	82	1105
WBYY	EEEE	303	60	1338
WBYY	EEEE	306	63	1378
WBYY	EEEE	318	52	1473
WBYY	EEEE	318	52	1515
WBYY	HHHH	325	70	1517
WBYY	EEEE	298	59	1585
WBYY	WWGY	326	79	1592
WBYY	WW__	337	71	1686
WY_G	WYY_	580	460	89
WY_G	ZZZZ	580	460	90
WY_G	WYY_	579	456	104
WY_G	XXXX	558	443	110
WY_G	XXXX	558	441	123
WY_G	XXXX	543	447	127
WY_G	WBGR	553	436	182
WY_G	ZZZZ	533	441	201
WY_G	WYBG	549	441	206
WY_G	XXXX	504	345	222
WY_G	JJJJ	486	322	225

WY_G	XXXX	500	321	228
WY_G	JJJJ	494	310	229
WY_G	PPPP	510	288	233
WY_G	OOOO	528	271	234
WY_G	PPPP	525	265	235
WY_G	PPPP	514	265	247
WY_G	WRWB	498	265	248
WY_G	WGG_	496	325	257
WY_G	MMMM	493	396	320
WY_G	WYBG	485	381	355
WY_G	MMMM	485	388	390
WY_G	MMMM	489	393	401
WY_G	MMMM	493	393	783
WY_G	AAAA	494	392	792
WY_G	AAAA	494	393	842
WY_G	XXXX	495	385	900
WY_G	AAAA	500	385	901
WY_G	AAAA	509	384	910
WY_G	XXXX	506	388	913
WY_G	MMMM	506	391	1185
WY_G	MMMM	506	389	1215
WY_G	MMMM	494	388	1230
WY_G	JJJJ	508	315	1246
WY_G	WBGG	508	293	1248
WY_G	AAAA	413	256	1253
WY_G	PPPP	519	261	1264
WY_G	AAAA	495	296	1267
WY_G	WBGG	466	234	1272
WY_G	WYG_	451	234	1274
WY_G	W_GY	388	224	1294
WY_G	WYG_	397	229	1298
WY_G	WYG_	404	233	1365
WY_G	WGGW	414	253	1369
WY_G	WYG_	414	270	1557
WY_G	XXXX	409	272	1558
WY_G	WBGG	408	236	1563
WY_G	W_GY	382	257	1599
WY_G	W_GY	398	246	1654
WY_G	W_RG	408	246	1679
WY_G	W_RG	390	307	1692
WY_G	WGGW	379	332	1698
WY_G	W_RG	381	355	1702
WY_G	WBGG	394	345	1705
XXXX	WY_G	564	422	103
XXXX	WYG_	566	415	107
XXXX	WYBG	512	267	181
XXXX	OOOO	550	305	224
XXXX	OOOO	535	291	305
XXXX	OOOO	539	298	417

XXXX	WRWB	539	298	417
XXXX	OOOO	519	277	505
XXXX	AAAA	502	275	665
XXXX	OOOO	519	277	669
XXXX	AAAA	509	267	676
XXXX	AAAA	505	267	709
XXXX	WYG_	510	264	864
XXXX	WW__	515	267	867
XXXX	WYG_	515	267	869
XXXX	PPPP	522	247	879
XXXX	OOOO	512	282	882
XXXX	WYG_	499	295	888
XXXX	WW__	445	256	920
XXXX	WW__	429	229	927
XXXX	WGG_	460	239	975
XXXX	WGG_	459	247	989
XXXX	WYG_	469	276	994
XXXX	WYG_	478	276	1006
XXXX	WBGG	448	285	1256
XXXX	IIII	452	384	1360
XXXX	IIII	447	384	1370
XXXX	WGGW	478	353	1399
XXXX	WWY_	542	400	1406
XXXX	VVVV	571	361	1433
XXXX	OOOO	520	303	1472
XXXX	PPPP	515	273	1478
XXXX	W_BG	524	251	1480
XXXX	WYG_	483	247	1487
XXXX	JJJJ	499	259	1493
XXXX	WYG_	495	456	1494
XXXX	MMMM	511	393	1540
WYY_	XXXX	598	444	154
WYY_	UUUU	604	440	157
WYY_	UUUU	604	442	166
WYY_	W_GW	615	467	312
WYY_	WGRB	601	461	317
WYY_	UUUU	627	446	331
WYY_	UUUU	636	446	348
WYY_	UUUU	600	464	564
WYY_	MMMM	542	431	661
WYY_	XXXX	558	441	1238
WYY_	WWY_	563	449	1392
WYY_	WWY_	574	445	1405
WYY_	WG__	553	464	1493
WYY_	WG__	545	446	1572
WYY_	WG__	573	462	1589
WYY_	WG__	574	468	1599
UUUU	WYY_	605	416	166
UUUU	WYY_	605	416	329

UUUU	WBWY	632	414	415
UUUU	W_GW	641	464	395
ZZZZ	UUUU	641	469	19
ZZZZ	W_GW	642	484	71
ZZZZ	W_GW	621	498	130
ZZZZ	WYY_	609	495	133
ZZZZ	WYY_	585	476	136
ZZZZ	WY_G	579	472	137
ZZZZ	W_GW	617	496	149
ZZZZ	WY_G	578	500	154
ZZZZ	WYY_	578	483	173
ZZZZ	WY_G	572	489	182
ZZZZ	WY_G	572	489	196
ZZZZ	WYBG	563	464	198
ZZZZ	WY_G	552	452	201
ZZZZ	JJJJ	484	336	207
ZZZZ	WRWB	465	290	211
ZZZZ	WYG_	417	207	214
ZZZZ	WYRW	356	150	217
ZZZZ	HHHH	370	131	219
ZZZZ	WBYY	367	76	221
ZZZZ	WBGW	269	68	239
ZZZZ	EEEE	260	68	240
ZZZZ	WBYY	310	65	251
ZZZZ	FFFF	310	63	254
ZZZZ	WBYY	321	66	255
ZZZZ	FFFF	301	72	257
ZZZZ	_R__	300	100	259
ZZZZ	WYYB	289	100	260
ZZZZ	WBGR	270	107	262
ZZZZ	WYYR	260	116	265
ZZZZ	EEEE	238	113	280
ZZZZ	EEEE	244	103	316
ZZZZ	WBYY	308	70	332
ZZZZ	WBGW	304	70	337
ZZZZ	WBYY	304	61	297
ZZZZ	WG_W	462	68	391
ZZZZ	WGRB	461	81	392
ZZZZ	WGRB	462	81	398
ZZZZ	WG_W	471	75	399
ZZZZ	LLLL	526	52	442
ZZZZ	WYWY	523	81	446
ZZZZ	WGRB	469	92	453
ZZZZ	WW_W	466	92	454
ZZZZ	WGRB	455	106	461
ZZZZ	WGWY	460	110	467
ZZZZ	WGRB	453	109	481
ZZZZ	WGWY	451	114	486
ZZZZ	WGRB	455	109	487

ZZZZ	WG_W	471	77	494
ZZZZ	WGRB	464	105	500
ZZZZ	W_RG	486	114	502
ZZZZ	WY__	498	142	504
ZZZZ	_YYY	504	152	505
ZZZZ	WYG_	478	173	508
ZZZZ	W__W	477	178	509
ZZZZ	W_BG	482	213	510
ZZZZ	WRWB	469	239	511
ZZZZ	XXXX	495	268	513
ZZZZ	WRWB	487	263	513
ZZZZ	JJJJ	474	274	521
ZZZZ	XXXX	474	283	522
ZZZZ	WGG_	492	258	524
ZZZZ	WRWB	463	259	529
ZZZZ	W__W	467	252	533
ZZZZ	WYG_	431	198	537
ZZZZ	W_W_	395	213	540
ZZZZ	WGGW	306	252	544
ZZZZ	W_GY	302	270	555
ZZZZ	WYYG	279	293	560
ZZZZ	WWR_	276	359	566
ZZZZ	WWR_	216	382	759
ZZZZ	W_GY	261	323	818
ZZZZ	WYYG	262	319	818
ZZZZ	W_GY	326	324	862
ZZZZ	W_GY	320	319	874
ZZZZ	W_RG	352	215	926
ZZZZ	WYRW	345	207	930
ZZZZ	WBGG	306	225	931
ZZZZ	W_W_	297	266	945
ZZZZ	W_GY	295	277	946
ZZZZ	WBGG	309	218	970
ZZZZ	W_W_	302	263	1032
ZZZZ	WYYB	306	216	1059
ZZZZ	WW__	316	240	1077
ZZZZ	WW__	278	196	1089
ZZZZ	WGYW	271	155	1099
ZZZZ	WWGY	271	155	1099
ZZZZ	WGYW	280	125	1131
ZZZZ	WBGW	305	125	1133
ZZZZ	WBYY	307	110	1133
ZZZZ	WGYW	307	103	1149
ZZZZ	WBGG	312	149	1167
ZZZZ	WYYB	298	166	1169
ZZZZ	W_RG	388	158	1239
ZZZZ	WBGG	473	216	1249
ZZZZ	JJJJ	478	222	1249
ZZZZ	WBGG	475	229	1250

ZZZZ	JJJJ	475	235	1252
ZZZZ	WGG_	503	160	1260
ZZZZ	WRWB	521	184	1262
ZZZZ	WGG_	521	177	1278
ZZZZ	WRWB	499	193	1285
ZZZZ	WGG_	453	198	1312
ZZZZ	WRWB	512	171	1319
ZZZZ	WW_W	493	148	1321
ZZZZ	WYWY	510	108	1323
ZZZZ	_YYY	517	108	1325
ZZZZ	WYWY	517	99	1342
ZZZZ	WW_W	479	104	1346
ZZZZ	WGRB	501	96	1365
ZZZZ	WYWY	514	89	1367
ZZZZ	WW_W	474	96	1376
ZZZZ	WY_R	427	81	1379
ZZZZ	WW__	456	72	1392
ZZZZ	WYRW	506	117	1402
ZZZZ	WYWY	523	97	1404
ZZZZ	_YYY	531	109	1411
ZZZZ	WGG_	435	188	1433
ZZZZ	JJJJ	487	222	1453
ZZZZ	XXXX	467	214	1471
ZZZZ	WGG_	430	200	1475
ZZZZ	WRWB	505	202	1534
ZZZZ	PPPP	533	242	1550
ZZZZ	WGRB	612	143	1655
ZZZZ	TTTT	608	230	1678
ZZZZ	TTTT	622	227	1685
ZZZZ	TTTT	617	230	1729
ZZZZ	W__W	570	192	1756
ZZZZ	W__W	585	188	1764
VVVV	AAAB	597	349	127
VVVV	AAAB	613	339	350
VVVV	WR__	589	320	370
VVVV	WR__	593	309	445
VVVV	AAAB	611	329	476
VVVV	XXXX	587	327	1450
VVVV	WR__	596	289	1768
W_GW	ZZZZ	639	482	85
W_GW	ZZZZ	635	484	129
W___	UUUU	624	436	655
W___	WYY_	574	421	730
W___	VVVV	620	372	934
W___	WBGR	672	388	1033
W___	UUUU	676	413	1217
W___	WBGR	676	375	1240
AAAB	WR__	618	300	371
AAAB	VVVV	647	330	1639

AAAB	WR__	625	296	1659
AAAB	VVVV	639	333	1759
AAAB	VVVV	639	316	1783
AAAB	__YR	655	307	1792
__YR	AAAB	675	294	294
_Y_B	WBWY	629	360	72
_Y_B	WW__	490	0	795
_Y_B	WG_W	490	0	796
_Y_B	FFFF	476	15	798
_Y_B	LLLL	476	15	798
_Y_B	WGRB	459	54	803
_Y_B	WGYW	453	54	804
_Y_B	WGRB	447	54	884
_Y_B	HHHH	443	51	899
_Y_B	WG_W	492	64	906
_Y_B	WYWY	492	64	906
_Y_B	LLLL	488	51	922
_Y_B	HHHH	472	55	932
_Y_B	HHHH	434	46	936
_Y_B	WG_W	454	63	944
_Y_B	HHHH	457	63	948
_Y_B	WBWY	630	326	1003
_Y_B	WBWY	630	372	1167
_Y_B	FFFF	485	0	1556
_Y_B	WW__	481	1	1557
_Y_B	WYRW	466	67	1561
_Y_B	WW__	451	67	1563
_Y_B	WYRW	451	67	1567
_Y_B	WW__	442	58	1587
_Y_B	WY_R	455	67	1629
_Y_B	WY_R	477	70	1654
_Y_B	WY_R	475	70	1655
_Y_B	WBWY	475	72	1661
_Y_B	WY_R	475	72	1671
_Y_B	_YYY	493	79	1674
_Y_B	PPPP	497	81	1712
_Y_B	WY_R	500	73	1717
_Y_B	PPPP	500	73	1719
_Y_B	WY_R	497	79	1720
_Y_B	PPPP	497	79	1720
_Y_B	WG_W	471	71	1721
_Y_B	WRGG	469	57	1722
_Y_B	WG_W	465	57	1722
_Y_B	WG_W	465	41	1742
_Y_B	WW__	478	38	1745
_Y_B	WYG_	481	24	1748
_Y_B	WW__	436	35	1750
_Y_B	WG_W	477	27	1751
WRGW	WYWW	724	283	50

WRGW	WYWW	724	278	62
WYWW	W__G	708	203	75
WYWW	W__G	720	211	93
WYWW	W__G	731	217	117
WYWW	TTTT	667	214	135
WYWW	WRR_	713	146	261
WYWW	W__G	657	184	267
WYWW	TTTT	670	225	366
WYWW	TTTT	670	225	403
WYWW	__YR	682	253	416
WYWW	W__G	719	184	973
WYWW	W__G	719	184	1358
WYWW	W__G	721	187	1375
W__G	WRR_	671	179	1
W__G	WRR_	683	185	10
W__G	WRR_	679	149	40
W__G	WY_R	630	149	51
W__G	WRR_	677	146	60
W__G	WYWW	713	189	122
W__G	WYWW	715	194	180
W__G	WRR_	683	173	186
W__G	WY_R	632	151	205
W__G	WRR_	650	145	234
W__G	WYWW	677	174	874
W__G	WRR_	630	167	881
W__G	WYWW	674	181	885
W__G	WRR_	674	162	891
W__G	WRR_	695	144	912
W__G	WYWW	676	184	983
W__G	_WRR	676	184	984
W__G	WRR_	672	174	986
W__G	WRR_	669	169	1000
W__G	WWRY	697	138	1090
W__G	WRR_	700	138	1118
W__G	WRR_	700	138	1135
W__G	_WRR	700	138	1266
W__G	_WRR	705	140	1279
W__G	_WRR	711	146	1298
W__G	WYWW	714	160	1304
W__G	WYWW	716	160	1358
W__G	WYWW	725	168	1384
W__G	WWRY	684	104	1440
W__G	_WRR	672	166	1551
W__G	WWGY	667	139	1645
W__G	WY_R	645	152	1652
W__G	_WRR	650	168	1657
W__G	WY_R	628	178	1665
W__G	WGWY	628	285	1667
W__G	WY_R	616	195	1673

W__G	WY_R	617	175	1686
W__G	W__W	608	165	1688
W__G	W_BG	592	169	1691
W__G	W__W	592	169	1693
W__G	W_BG	585	176	1696
W__G	W__W	582	175	1697
W__G	W_BG	557	180	1699
W__G	W__W	548	167	1704
W__G	HHHH	531	169	1710
W__G	WYBG	531	150	1716
W__G	HHHH	525	145	1718
W__G	WYBG	527	139	1721
W__G	WY__	506	133	1724
W__G	WRWB	467	145	1732
W__G	WW_W	471	143	1734
W__G	WRWB	471	147	1738
W__G	WYBG	489	133	1742
W__G	WW_W	484	121	1746
W__G	WYBG	498	117	1753
W__G	WGRB	531	122	1777
TTTT	WGWY	604	228	1608
TTTT	ZZZZ	605	228	1608
TTTT	WR__	625	256	1623
TTTT	WR__	632	364	1712
TTTT	WR__	632	253	1735
TTTT	W_BG	584	231	1751
TTTT	ZZZZ	619	212	1767
WR__	OOOO	575	281	1673
WR__	TTTT	612	284	1708
WR__	TTTT	622	282	1713
WR__	TTTT	632	374	1720
WR__	OOOO	564	255	1779
WR__	WYG_	548	255	1781
WR__	OOOO	552	261	1782
WR__	WYG_	552	261	1783
WR__	WYG_	505	283	1789
WR__	XXXX	508	337	1792
OOOO	XXXX	539	283	240
OOOO	XXXX	539	283	261
OOOO	XXXX	539	282	614
OOOO	XXXX	535	281	621
OOOO	XXXX	535	279	800
OOOO	XXXX	538	284	808
OOOO	XXXX	536	284	817
WRR_	W__G	683	144	908
WRR_	W__G	675	149	934
WRR_	W__G	671	138	953
WRR_	W__G	681	160	968
WRR_	WGRB	636	143	999

WRR_	TTTT	636	152	1001
WRR_	WGRB	630	148	1008
WRR_	WGRB	618	154	1017
WRR_	W__G	674	157	1034
WRR_	W__G	669	151	1059
WRR_	W__G	673	142	1116
WRR_	QQQQ	618	78	1398
WRR_	WWRY	642	68	1485
WRR_	WWRY	650	88	1497
WRR_	W__W	630	107	1529
WRR_	WWRY	647	79	1556
WRR_	WWRY	640	65	1602
WRR_	QQQQ	623	60	1608
WRR_	WGRB	633	95	1651
WRR_	WWRY	633	80	1656
WWRY	WY_R	650	60	35
WWRY	WY_R	650	60	94
WWRY	WRGG	645	68	327
WWRY	WYBG	660	51	933
WWRY	WRGG	661	51	942
WWRY	WYBG	661	51	947
WWRY	WY_R	661	43	1002
WWRY	WY_R	668	54	1015
WWRY	WY_R	669	51	1023
WWRY	WYBG	634	75	1058
WWRY	WY_R	634	72	1059
WWRY	WYBG	660	83	1069
WWRY	WY_R	637	61	1153
WWRY	_WRR	649	61	1196
WWRY	WRR_	645	77	1657
WWRY	WRR_	645	64	1676
WWRY	RRRR	674	48	1730
PPPP	WGG_	540	179	415
PPPP	WY__	489	165	450
PPPP	W_RG	492	143	452
PPPP	_YYY	519	144	461
PPPP	W_BG	497	205	497
PPPP	W_BG	556	223	1666
PPPP	WGWY	554	173	1677
PPPP	WYBG	526	87	1694
PPPP	_Y_B	516	81	1697
PPPP	WBGG	461	306	1762
PPPP	W_BG	494	247	1779
PPPP	WW_W	453	115	1793
WRWB	WG__	468	231	228
WRWB	W_BG	483	258	263
WRWB	W_BG	486	250	281
WRWB	W_BG	483	250	314
WRWB	WGG_	495	267	380

WRWB	W_BG	495	249	384
WRWB	WGG_	520	245	389
WRWB	OOOO	534	268	404
WRWB	OOOO	543	275	415
WRWB	XXXX	520	289	418
WRWB	JJJJ	473	271	468
WRWB	W__W	464	244	507
WRWB	W_W_	422	213	550
WRWB	W_GY	338	270	578
WRWB	W_GY	289	295	625
WRWB	WYYG	259	265	712
WRWB	WGGW	278	271	719
WRWB	WWGY	375	187	799
WRWB	WWGY	399	182	821
WRWB	W_RG	439	145	846
WRWB	WWGY	421	136	859
WRWB	W_W_	554	191	925
WRWB	_YYY	551	117	927
WRWB	W_W_	560	163	934
WRWB	_YYY	573	153	1036
WRWB	_YYY	571	155	1133
WRWB	W__W	568	155	1248
WRWB	ZZZZ	528	184	1277
WRWB	JJJJ	525	193	1369
WRWB	ZZZZ	494	158	1432
WRWB	AAAA	504	158	1490
WRWB	W_RG	502	158	1499
WRWB	WY_R	514	161	1600
WRWB	W_RG	505	158	1608
WRWB	WYG_	505	178	1668
WRWB	JJJJ	517	191	1671
WRWB	JJJJ	521	202	1682
WRWB	WYG_	479	183	1707
WRWB	WYG_	480	185	1719
WRWB	WGG_	440	163	1738
WRWB	WYRW	422	141	1745
WGWY	TTTT	621	203	894
WGWY	W_BG	621	203	1051
WGWY	TTTT	642	214	1231
WGWY	WRWB	589	178	1271
WGWY	_WRR	637	188	1460
WGWY	WR__	616	219	1484
WGWY	TTTT	627	212	1486
WGWY	_WRR	642	192	1492
WGWY	W__W	637	151	1500
WGWY	XXXX	580	204	1541
WGWY	W_BG	618	216	1654
WGWY	W_BG	593	209	1668
WGWY	W__W	578	180	1670

WGWY	WY_R	576	182	1671
WGWY	PPPP	569	182	1677
WGWY	W__W	565	176	1680
WGWY	JJJJ	480	225	1700
WGWY	IIII	416	414	1742
WGWY	WGGY	404	427	1743
WGWY	IIII	411	436	1744
WGWY	W_GW	613	472	1763
RRRR	QQQQ	597	39	111
RRRR	LLLL	520	39	149
RRRR	WY_R	611	102	237
RRRR	WY_R	570	108	253
RRRR	WYWY	524	108	267
RRRR	WRGG	524	108	270
RRRR	WYWY	524	111	291
RRRR	ZZZZ	534	89	445
RRRR	WYWY	534	90	447
RRRR	WYWY	538	92	479
RRRR	W_RG	538	92	482
RRRR	WYWY	538	92	485
RRRR	WYWY	536	92	501
RRRR	WYWY	538	92	525
RRRR	WYWY	538	92	563
RRRR	W_RG	533	102	570
RRRR	W_RG	538	98	590
RRRR	W_RG	535	98	631
RRRR	W_RG	541	102	641
RRRR	WRGG	567	99	648
RRRR	WY_R	567	99	649
RRRR	WRGG	568	95	664
RRRR	WY_R	568	97	665
RRRR	WYWY	535	98	754
RRRR	WRGG	553	79	760
RRRR	WRGG	567	72	816
RRRR	WYWY	546	75	821
RRRR	WG_W	546	75	822
RRRR	WY_R	569	95	839
RRRR	WYBG	567	98	883
RRRR	WYWY	566	98	905
RRRR	WYBG	566	96	908
RRRR	WYWY	569	90	1127
RRRR	WYWY	561	79	1132
RRRR	WY_R	570	65	1137
RRRR	WYWY	567	71	1142
RRRR	WYBG	567	87	1145
RRRR	WYWY	561	89	1147
RRRR	WYBG	565	86	1191
RRRR	WYWY	567	86	1193
RRRR	WYWY	565	84	1241

RRRR	WYWY	560	82	1278
RRRR	WYWY	560	84	1361
RRRR	WYWY	573	76	1389
RRRR	WYBG	766	79	1393
RRRR	WRR_	594	89	1398
RRRR	WYWY	584	58	1406
RRRR	WYBG	581	80	1417
RRRR	WYBG	583	89	1424
RRRR	WYBG	576	87	1439
RRRR	WYWY	585	64	1550
RRRR	WRR_	592	80	1559
RRRR	WRR_	597	65	1572
RRRR	WRR_	599	68	1591
RRRR	WWRY	629	45	1610
QQQQ	WRGG	563	66	37
QQQQ	LLLL	521	44	46
QQQQ	LLLL	517	41	69
QQQQ	WRGG	545	71	71
QQQQ	WG_W	509	48	98
QQQQ	RRRR	571	48	102
QQQQ	LLLL	522	41	118
QQQQ	WG_W	504	35	127
_WRR	WYBG	590	219	11
_WRR	WYBG	593	211	16
_WRR	W_RG	597	179	55
_WRR	WYBG	604	186	90
_WRR	WRGG	604	186	182
_WRR	WY_R	604	186	297
_WRR	WYBG	604	186	488
_WRR	W__W	604	186	908
_WRR	WRR_	630	169	933
_WRR	WYWW	665	184	946
_WRR	WYWW	687	226	1094
_WRR	__YR	685	247	1103
_WRR	WYWW	685	230	1109
_WRR	W__G	668	190	1115
_WRR	WRR_	666	185	1116
_WRR	W__G	673	185	1117
_WRR	WGWY	659	185	1122
_WRR	WRR_	657	179	1123
_WRR	W__G	666	178	1125
_WRR	WRR_	666	168	1126
_WRR	W__G	675	168	1129
_WRR	WRR_	654	168	1134
_WRR	WWRY	642	89	1152
_WRR	WY_R	633	89	1155
_WRR	WYBG	622	94	1158
_WRR	WWRY	660	79	1170
_WRR	WWRY	679	65	1213

_WRR	W__G	687	102	1255
_WRR	W__G	697	121	1260
_WRR	W__G	705	150	1357
_WRR	WYWW	672	184	1371
_WRR	WYWW	672	205	1413
WYG_	WY__	454	167	250
WYG_	W_BG	492	204	285
WYG_	WRWB	472	239	289
WYG_	_YYY	516	193	300
WYG_	_YYY	523	180	317
WYG_	W_BG	517	195	351
WYG_	W_BG	522	195	373
WYG_	W__W	482	195	382
WYG_	W_BG	475	212	401
WYG_	W__W	475	212	401
WYG_	WY__	458	185	410
WYG_	WG__	399	169	417
WYG_	WWGY	399	169	418
WYG_	WWGY	412	200	433
WYG_	PPPP	455	162	490
WYG_	FFFF	455	165	538
WYG_	W__W	456	165	543
WYG_	W_BG	484	200	572
WYG_	WW__	495	182	837
WYG_	XXXX	498	246	853
WYG_	XXXX	498	255	866
WYG_	WGG_	472	314	896
WYG_	XXXX	504	330	902
WYG_	WY_G	521	348	905
WYG_	JJJJ	537	362	913
WYG_	WY_G	506	374	947
WYG_	WGG_	489	330	964
WYG_	JJJJ	503	338	973
WYG_	WGG_	491	260	1000
WYG_	JJJJ	501	247	1002
WYG_	WWGY	482	163	1014
WYG_	W_RG	398	163	1031
WYG_	WY_G	423	219	1276
WYG_	WY_G	423	222	1303
WYG_	WWY_	478	245	1454
WYG_	WWY_	482	245	1480
WYG_	JJJJ	482	241	1488
WYG_	WRWB	505	184	1603
WYG_	W_RG	474	190	1622
WYG_	WRWB	499	184	1646
WYG_	WY_R	461	189	1652
WYG_	WGG_	428	172	1656
WYG_	WG_W	442	165	1683
WYG_	WWY_	480	223	1723

WYG_	WR__	531	252	1782
WY_R	WRGG	607	128	25
WY_R	WWRY	626	84	35
WY_R	WRGG	599	105	42
WY_R	WRGG	599	91	144
WY_R	W__G	621	128	206
WY_R	_WRR	612	147	226
WY_R	_WRR	614	154	269
WY_R	W__G	628	150	272
WY_R	W__G	624	153	287
WY_R	WRGG	611	136	293
WY_R	WRGG	588	107	304
WY_R	RRRR	570	83	324
WY_R	WRGG	586	78	334
WY_R	RRRR	571	82	341
WY_R	WRGG	587	96	365
WY_R	_YYY	549	130	438
WY_R	RRRR	544	112	448
WY_R	WRGG	613	103	468
WY_R	RRRR	588	116	649
WY_R	WRGG	588	116	805
WY_R	WYBG	595	118	835
WY_R	WRGG	595	118	867
WY_R	WYBG	595	118	896
WY_R	WYBG	602	118	917
WY_R	WGRB	602	114	938
WY_R	WRGG	612	91	946
WY_R	WWRY	638	83	955
WY_R	WRGG	631	81	956
WY_R	WWRY	637	76	958
WY_R	WWRY	637	86	974
WY_R	WWRY	670	49	1042
WY_R	WYBG	634	68	1076
WY_R	RRRR	601	72	1080
WY_R	WYBG	601	77	1083
WY_R	WYWY	565	67	1110
WY_R	WRGG	559	50	1118
WY_R	WYWY	568	61	1125
WY_R	WRGG	560	48	1140
WY_R	RRRR	579	65	1146
WY_R	_WRR	594	65	1147
WY_R	WYBG	598	73	1149
WY_R	WWRY	615	57	1153
WY_R	_WRR	617	65	1155
WY_R	W_BG	617	195	1226
WY_R	W_RG	407	180	1252
WY_R	WGYW	281	95	1321
WY_R	HHHH	292	70	1332
WY_R	WGYW	386	56	1357

WY_R	WGYW	408	66	1376
WY_R	WGYW	566	76	1408
WY_R	_YYY	548	90	1454
WY_R	WGYW	554	90	1509
WY_R	_Y_B	433	80	1611
WY_R	WW__	387	67	1639
WY_R	_Y_B	455	61	1653
WY_R	_Y_B	455	70	1662
WY_R	WW_W	440	94	1669
WY_R	_Y_B	449	82	1671
WY_R	WGYW	429	86	1715
WYBG	_YYY	565	176	4
WYBG	WRWB	512	262	33
WYBG	WR__	584	273	72
WYBG	_WRR	602	230	74
WYBG	_YYY	562	207	81
WYBG	W_RG	565	213	84
WYBG	_WRR	585	196	92
WYBG	_YYY	558	207	125
WYBG	_YYY	547	220	146
WYBG	OOOO	543	257	161
WYBG	WRWB	522	264	173
WYBG	WGG_	471	373	180
WYBG	MMMM	480	385	181
WYBG	XXXX	489	385	182
WYBG	WY_G	549	426	193
WYBG	WBGR	557	426	240
WYBG	WBGR	557	437	266
WYBG	MMMM	538	433	268
WYBG	WBGR	547	473	310
WYBG	WGG_	537	453	317
WYBG	WGG_	537	434	329
WYBG	WY_G	508	370	336
WYBG	WBGR	533	404	381
WYBG	MMMM	519	411	386
WYBG	WBGR	545	404	389
WYBG	XXXX	506	337	416
WYBG	JJJJ	480	311	420
WYBG	WRWB	492	300	422
WYBG	XXXX	506	294	441
WYBG	_WRR	577	194	489
WYBG	WGG_	558	173	502
WYBG	W_BG	563	165	737
WYBG	_YYY	563	169	792
WYBG	W_BG	565	169	796
WYBG	WY_R	582	142	835
WYBG	W__W	563	154	856
WYBG	RRRR	563	113	870
WYBG	_YYY	543	119	873

WYBG	RRRR	549	111	890
WYBG	W__W	559	144	904
WYBG	RRRR	548	114	906
WYBG	WYWY	548	114	906
WYBG	WGRB	548	114	907
WYBG	WY_R	571	114	909
WYBG	WRGG	579	114	911
WYBG	WY_R	579	114	912
WYBG	RRRR	576	114	913
WYBG	WY_R	583	111	914
WYBG	WRGG	583	101	915
WYBG	WY_R	609	97	918
WYBG	WWRY	645	84	932
WYBG	W__G	694	121	989
WYBG	WGRB	612	94	1040
WYBG	WY_R	609	97	1080
WYBG	WGRB	600	97	1085
WYBG	RRRR	586	99	1089
WYBG	_YYY	604	112	1090
WYBG	RRRR	579	114	1106
WYBG	_YYY	576	114	1119
WYBG	W__W	561	144	1614
WYBG	WRGG	564	112	1632
WYBG	WGRB	564	112	1644
WYBG	WRGG	557	108	1651
WYBG	_YYY	549	114	1653
WYBG	_YYY	519	109	1678
WYBG	_Y_B	503	109	1681
WYBG	WY__	503	99	1710
WYBG	WG_W	512	112	1714
WYBG	WY__	503	117	1718
WYBG	_Y_B	504	89	1722
WYBG	WRGG	510	106	1740
WYBG	WGRB	520	106	1754
WYBG	W__G	517	106	1755
WYBG	WGRB	520	103	1757
WYBG	WGG_	443	180	1769
WYBG	WBGG	411	218	1782
WYBG	WGG_	376	177	1796
_YYY	W_RG	530	194	65
_YYY	W_RG	542	190	111
_YYY	W_BG	541	195	190
_YYY	WRGG	541	195	197
_YYY	W_RG	538	189	263
_YYY	WYG_	516	198	286
_YYY	WYG_	540	193	299
_YYY	WYG_	542	186	315
_YYY	WYG_	540	189	367
_YYY	W_RG	526	164	377

_YYY	WYWY	527	136	399
_YYY	WWGY	531	121	941
_YYY	WYWY	541	110	953
_YYY	RRRR	559	110	964
_YYY	WGRB	561	110	966
_YYY	WGRB	566	125	1007
_YYY	W__W	588	137	1034
_YYY	WGRB	600	141	1068
_YYY	WYBG	606	133	1090
_YYY	WYBG	595	127	1118
_YYY	WY__	567	119	1150
_YYY	RRRR	559	104	1160
_YYY	WYWY	549	104	1169
_YYY	WGYW	542	104	1509
_YYY	WRGG	535	92	1585
_YYY	WY__	530	102	1590
_YYY	WG_W	526	120	1593
_YYY	WG_W	515	125	1641
_YYY	WRWB	515	130	1648
_YYY	WRGG	505	81	1664
_YYY	_Y_B	517	79	1680
_YYY	WYWY	542	67	1687
_YYY	_Y_B	519	67	1717
_YYY	WYWY	567	53	1755
_YYY	WYG_	522	42	1759
_YYY	WRGG	515	38	1780
W_RG	_YYY	520	208	12
W_RG	WY__	520	174	17
W_RG	WYWY	538	151	25
W_RG	WY__	538	164	34
W_RG	_YYY	542	188	48
W_RG	WYWY	523	156	60
W_RG	WRWB	480	204	70
W_RG	_YYY	557	192	84
W_RG	WYWY	541	142	98
W_RG	_YYY	541	169	116
W_RG	WYWY	523	143	129
W_RG	WYWY	495	114	454
W_RG	WW_W	478	94	612
W_RG	WYWY	493	94	614
W_RG	WG_W	499	75	796
W_RG	WW__	508	99	803
W_RG	WYWY	510	99	808
W_RG	WW__	502	103	811
W_RG	WW__	463	70	822
W_RG	_Y_B	461	70	823
W_RG	WGRB	443	95	829
W_RG	WRWB	427	127	846
W_RG	WW__	450	158	851

W_RG	JJJJ	403	266	863
W_RG	WRWB	423	181	873
W_RG	WWGY	418	174	875
W_RG	WWGY	392	173	891
W_RG	WYG_	378	192	1067
W_RG	WBGG	376	199	1199
W_RG	WGRB	376	173	1205
W_RG	WGRB	410	154	1444
W_RG	WGG_	406	154	1445
W_RG	WGRB	422	154	1453
W_RG	WRWB	494	168	1519
W_RG	WGG_	424	155	1566
W_RG	WW_W	441	121	1575
W_RG	WG_W	479	147	1605
W_RG	WWGY	479	165	1629
W_RG	WYG_	471	175	1634
W_RG	WY_G	413	223	1640
W_RG	WBGG	443	284	1645
W_RG	WWY_	409	278	1670
W_RG	WY_G	409	265	1674
W_RG	WGGW	377	327	1687
W_RG	WGGY	358	455	1773
W_RG	WGGW	321	410	1783
W_RG	WYYG	307	328	1790
W_BG	_YYY	518	188	186
W_BG	PPPP	495	240	245
W_BG	WRWB	486	237	309
W_BG	WRWB	489	230	464
W_BG	AAAA	480	203	638
W_BG	_YYY	550	138	716
W_BG	WY_R	565	124	731
W_BG	_YYY	550	133	755
W_BG	W__W	527	176	819
W_BG	W_RG	487	165	850
W_BG	WRWB	424	184	860
W_BG	WY__	491	186	981
W_BG	W__W	580	179	1007
W_BG	WGWY	601	185	1050
W_BG	WY__	534	151	1589
W_BG	WY__	522	151	1595
W_BG	PPPP	550	223	1608
W_BG	WRWB	525	196	1725
W_BG	HHHH	546	208	1738
W_BG	WR__	583	225	1780
WY__	WYWY	511	158	19
WY__	W_RG	516	163	36
WY__	WYWY	505	155	39
WY__	WYG_	462	177	46
WY__	WYWY	487	160	58

WY__	W_RG	485	160	73
WY__	WYWY	491	155	228
WY__	W_BG	491	165	232
WY__	WYWY	494	158	238
WY__	W_RG	499	154	244
WY__	W_RG	503	156	452
WY__	_YYY	513	161	457
WY__	_YYY	517	133	954
WY__	WWGY	515	151	975
WY__	HHHH	364	101	1014
WY__	WW_W	451	132	1069
WY__	JJJJ	494	169	1072
WY__	WYWY	555	116	1079
WY__	WW_W	478	102	1109
WY__	WGRB	521	151	1140
WY__	RRRR	544	106	1150
WY__	_YYY	552	117	1160
WY__	WRGG	533	122	1553
WY__	WRGG	514	113	1566
WY__	WG_W	516	113	1584
WY__	WY_R	535	113	1593
WY__	_YYY	512	118	1599
WY__	W_RG	508	140	1606
WY__	WG_W	473	137	1624
WY__	JJJJ	510	193	1665
WY__	WGWY	545	186	1671
WY__	W__W	548	163	1674
WY__	_Y_B	489	106	1682
WY__	WYBG	495	106	1710
WY__	_Y_B	486	101	1717
WY__	_Y_B	488	107	1734
WY__	WY_R	488	107	1734
WYWY	WY__	518	140	13
WYWY	W_RG	520	142	26
WYWY	WGRB	499	126	82
WYWY	WRGG	509	107	105
WYWY	WRGG	511	104	135
WYWY	WW_W	494	112	137
WYWY	RRRR	527	110	411
WYWY	WG_W	493	84	444
WYWY	RRRR	516	92	447
WYWY	HHHH	509	75	475
WYWY	RRRR	523	73	487
WYWY	WG_W	510	65	644
WYWY	WG_W	533	73	799
WYWY	RRRR	554	77	801
WYWY	W_RG	532	104	802
WYWY	WW__	519	112	826
WYWY	_Y_B	470	60	844

WYWY	WG_W	483	65	856
WYWY	RRRR	546	80	904
WYWY	WY__	555	94	1079
WYWY	RRRR	555	94	1083
WYWY	WRGG	558	67	1270
WYWY	RRRR	556	63	1277
WYWY	WRGG	555	59	1316
WYWY	RRRR	573	39	1549
WYWY	WRGG	558	65	1590
WYWY	WGRB	555	70	1626
WYWY	WRGG	557	68	1658
WYWY	_YYY	564	68	1687
WYWY	WRR_	576	68	1690
WYWY	_YYY	573	72	1692
WYWY	WRR_	590	76	1736
WYWY	_YYY	566	80	1745
WYWY	W__W	586	116	1767
WYWY	WYRW	566	109	1771
WYWY	W__W	605	140	1785
WYWY	XXXX	612	152	1787
WYWY	W__W	605	149	1793
WRGG	RRRR	569	106	7
WRGG	WYWY	554	106	11
WRGG	RRRR	575	91	26
WRGG	LLLL	520	52	37
WRGG	RRRR	544	66	40
WRGG	WY_R	567	106	42
WRGG	_YYY	541	122	49
WRGG	RRRR	535	90	72
WRGG	WY_R	578	112	80
WRGG	WYWY	526	102	89
WRGG	WGRB	520	95	91
WRGG	WG_W	502	67	96
WRGG	WGRB	490	63	98
WRGG	WG_W	490	53	103
WRGG	WW__	490	53	103
WRGG	WYWY	492	86	111
WRGG	WGRB	471	86	113
WRGG	WW_W	482	97	117
WRGG	WGRB	469	66	119
WRGG	WYWY	516	73	133
WRGG	WY_R	584	86	138
WRGG	_WRR	593	150	158
WRGG	W_RG	533	125	168
WRGG	WYWY	529	115	172
WRGG	_WRR	600	152	179
WRGG	_YYY	555	175	195
WRGG	WG__	533	171	244
WRGG	WY__	525	171	253

WRGG	WG__	512	163	264
WRGG	_YYY	519	176	266
WRGG	RRRR	523	131	269
WRGG	WY_R	605	112	285
WRGG	WWRY	622	98	296
WRGG	WY_R	595	77	306
WRGG	WWRY	630	68	315
WRGG	WY_R	601	80	335
WRGG	WY_R	604	82	468
WRGG	WY_R	613	91	624
WRGG	RRRR	579	86	640
WRGG	RRRR	573	69	760
WRGG	RRRR	576	86	786
WRGG	RRRR	594	71	912
WRGG	WYWY	546	62	984
WRGG	LLLL	544	52	987
WRGG	WYWY	541	71	1002
WRGG	WG_W	523	71	1152
WRGG	WGRB	513	53	1273
WRGG	WYWY	531	53	1352
WRGG	WYWY	528	53	1372
WRGG	XXXX	514	53	1374
WRGG	WW__	492	46	1395
WRGG	WW__	480	60	1423
WRGG	WGYW	493	94	1433
WRGG	_YYY	536	89	1519
WRGG	WYWY	543	70	1590
WRGG	WYBG	571	92	1592
WRGG	WRR_	611	96	1599
WRGG	WYBG	601	110	1600
WRGG	WYWY	574	82	1603
WRGG	_YYY	560	102	1613
WRGG	WYWY	533	65	1655
WRGG	_Y_B	517	59	1662
WRGG	_YYY	513	68	1664
WRGG	_Y_B	510	64	1667
WRGG	WBWY	468	50	1678
WRGG	_Y_B	472	50	1680
WRGG	WG_W	472	48	1719
WRGG	_Y_B	477	41	1723
WRGG	WG_W	477	41	1724
WRGG	_Y_B	484	56	1728
WRGG	WYBG	511	70	1732
WRGG	_Y_B	493	84	1736
WRGG	WYRW	465	91	1740
WRGG	WBWY	448	73	1743
WRGG	_Y_B	450	73	1744
WRGG	WYRW	451	81	1750
WRGG	WBWY	428	60	1753

WRGG	WYRW	431	54	1760
WRGG	WYG_	494	46	1768
WRGG	LLLL	490	24	1782
WGRB	WYWY	509	93	25
WGRB	WG_W	494	61	31
WGRB	WW__	465	65	96
WGRB	WW__	450	55	118
WGRB	WG_W	446	64	137
WGRB	WW__	453	68	142
WGRB	WG_W	467	68	165
WGRB	WW_W	460	89	175
WGRB	WG_W	468	78	304
WGRB	FFFF	460	78	365
WGRB	XXXX	448	78	458
WGRB	WG_W	461	65	477
WGRB	W_RG	450	90	613
WGRB	WW__	455	90	798
WGRB	_Y_B	425	56	852
WGRB	WWGY	430	120	876
WGRB	WYWY	479	86	887
WGRB	_Y_B	463	74	896
WGRB	WYWY	477	90	899
WGRB	_YYY	526	112	910
WGRB	WY_R	577	109	929
WGRB	WRWB	573	142	935
WGRB	WRR_	625	147	941
WGRB	_YYY	583	124	965
WGRB	WY_R	597	115	976
WGRB	RRRR	597	100	993
WGRB	_YYY	583	108	995
WGRB	_YYY	589	131	1008
WGRB	WYBG	602	117	1041
WGRB	RRRR	587	110	1080
WGRB	WY__	587	110	1081
WGRB	WYWY	553	106	1089
WGRB	WY__	530	127	1138
WGRB	WW_W	487	142	1147
WGRB	WBGG	399	160	1200
WGRB	W_RG	391	160	1211
WGRB	WW__	449	79	1246
WGRB	WRGG	481	79	1250
WGRB	WW__	467	68	1253
WGRB	WRGG	504	68	1272
WGRB	WYWY	516	73	1275
WGRB	WRGG	495	57	1284
WGRB	WW__	471	62	1310
WGRB	XXXX	478	96	1364
WGRB	WW_W	496	110	1384
WGRB	XXXX	431	95	1392

WGRB	W_RG	410	147	1404
WGRB	WYRW	410	147	1405
WGRB	WGG_	411	152	1406
WGRB	W_RG	430	141	1429
WGRB	WYRW	419	126	1432
WGRB	_R__	389	98	1460
WGRB	W_RG	418	119	1469
WGRB	WYRW	418	128	1470
WGRB	WGG_	418	145	1513
WGRB	WYYB	338	140	1520
WGRB	WWGY	351	122	1565
WGRB	WW__	385	62	1579
WGRB	_Y_B	425	66	1598
WGRB	WG_W	477	99	1605
WGRB	_YYY	515	93	1617
WGRB	WYWY	541	75	1621
WGRB	WRGG	543	78	1631
WGRB	WRR_	587	94	1637
WGRB	WYBG	591	101	1637
WGRB	XXXX	610	112	1639
WGRB	WRR_	616	112	1652
WGRB	W__W	606	130	1710
WGRB	WRR_	615	113	1718
WGRB	_YYY	552	113	1740
WGRB	WYBG	541	107	1744
WGRB	_YYY	535	89	1746
WGRB	LLLL	507	71	1754
WGRB	_YYY	519	71	1761
WGRB	WRGG	511	71	1774
WGRB	_YYY	519	62	1775
WGRB	WBWY	454	74	1785
WGRB	LLLL	448	73	1793
WG_W	QQQQ	491	9	128
WG_W	WW__	469	32	133
WG_W	WRGG	484	61	135
WG_W	WW__	464	51	136
WG_W	RRRR	514	55	221
WG_W	WW__	480	43	237
WG_W	WYWY	518	57	807
WG_W	WW__	512	52	819
WG_W	_Y_B	486	51	827
WG_W	WYWY	510	75	855
WG_W	_Y_B	497	46	859
WG_W	WYWY	509	61	867
WG_W	_Y_B	492	44	874
WG_W	_Y_B	459	81	942
WG_W	_Y_B	451	70	955
WG_W	_Y_B	631	63	989
WG_W	WBWY	633	317	1023

WG_W	_Y_B	603	354	1029
WG_W	_Y_B	626	358	1078
WG_W	WBWY	671	333	1109
WG_W	WGRB	493	128	1155
WG_W	WW_W	489	132	1193
WG_W	WW__	477	59	1195
WG_W	FFFF	498	29	1196
WG_W	WW__	471	46	1231
WG_W	FFFF	473	20	1234
WG_W	WBWY	672	296	1263
WG_W	FFFF	494	0	1576
WG_W	_Y_B	481	44	1578
WG_W	WRGG	509	60	1581
WG_W	_YYY	510	140	1594
WG_W	W_BG	510	140	1595
WG_W	W_BG	495	140	1634
WG_W	_YYY	497	140	1644
WG_W	WW_W	481	122	1652
WG_W	WRWB	502	134	1656
WG_W	WYG_	468	188	1668
WG_W	WYRW	468	147	1670
WG_W	WW_W	468	134	1672
WG_W	WYRW	453	142	1677
WG_W	WRGG	462	55	1718
WG_W	WBWY	449	55	1719
WG_W	WRGG	464	50	1724
WG_W	FFFF	481	0	1744
WG_W	WYG_	481	9	1747
LLLL	_Y_B	498	35	801
LLLL	_Y_B	484	16	836
LLLL	FFFF	491	16	900
LLLL	_Y_B	511	47	917
LLLL	FFFF	484	22	931
LLLL	FFFF	491	19	982
LLLL	WWGY	499	34	1058
LLLL	FFFF	496	34	1103
LLLL	_Y_B	477	37	1583
LLLL	WGYW	484	37	1702
LLLL	_Y_B	470	71	1749
LLLL	WBWY	463	76	1751
LLLL	WGRB	483	72	1752
LLLL	WYG_	474	37	1759
WBWY	_Y_B	642	380	26
WBWY	_Y_B	636	352	1004
WBWY	WG_W	667	314	1101
WBWY	_Y_B	622	367	1215
WBWY	WG_W	657	295	1264
WBWY	_Y_B	643	334	1305
WBWY	WG_W	691	364	1339

WBWY	_Y_B	614	395	1441
WBWY	LLLL	484	0	1652
WBWY	WW__	411	57	1667
WBWY	WYRW	422	71	1725
WBWY	WG_W	433	57	1727
WBWY	WRGG	430	57	1743
WBWY	_Y_B	439	55	1744

Antlist	InitialPosX	InitialPosY	ColonyArea	AntLength	MinX	MinY	MaxX	MaxY
WWR_	260	366	247446	50.1	196	0	746	510
WYYG	258	300						
AAAA	281	302						
W_W_	271	236						
W_WG	257	185						
WBGG	300	181						
WYRW	323	153						
WYYB	283	143						
WYYR	239	134						
WBGW	271	107						
EEEE	257	71						
WBYY	246	49						
FFFF	325	68						
WW__	370	62						
HHHH	364	86						
_R__	348	120						
WGGW	348	290						
WWY_	350	387						
W_GY	373	408						
WGGY	383	444						
IIII	453	420						
WGGG	474	461						
WGG_	443	372						
JJJJ	443	309						
WG__	423	237						
W__W	420	221						
WWGY	384	177						
WGYW	420	136						
WYG_	430	167						
WGRB	461	56						
LLLL	495	21						
WG_W	488	15						
WW_W	460	105						
WRWB	494	268						
MMMM	483	420						
WBGR	493	454						
XXXX	569	400						
WY_G	580	460						
OOOO	541	278						
PPPP	507	252						
WYWY	512	128						
WY__	513	166						
W_RG	513	203						
_YYY	546	193						
WYBG	576	181						
_WRR	589	217						
WY_R	617	138						
WRGG	567	111						
QQQQ	580	65						
RRRR	600	40						
WWRY	650	57						
WRR_	706	146						
W__G	671	184						
WGWY	621	206						
TTTT	630	262						
WR__	600	282						
WYWW	716	242						
WRGW	725	283						
__YR	686	310						
_Y_B	627	333						
UUUU	608	416						
VVVV	598	351						
W___	659	390						
WYY_	595	444						
ZZZZ	639	458						
W_GW	657	480						
WBWY	784	442						
W_BG	517	188						
AAAB	610	330						

Actor	Target	Time	ActorPosX	ActorPosY
___	WRG	44	0	0
___	WRG	365	0	0
___	WWR	58	0	0
___	WWR	73	0	0
___	WWR	351	0	0
___	WWR	421	0	0
___	WWR	480	0	0
___	WWR	651	0	0
___	WWR	791	0	0
___	WWR	838	0	0
___	YWW	35	0	0
___	YWW	47	0	0
_G_	RYW	165	0	0
_G_	RYW	197	0	0
_G_	RYW	452	0	0
_G_	WG_	610	0	0
_G_	WG_	1102	0	0
_G_	WGY	852	0	0
_G_	WRG	1139	0	0
_G_	YWW	406	0	0
_G_	YWW	469	0	0
RYW	_G_	167	0	0
RYW	_G_	202	0	0
RYW	_G_	451	0	0
RYW	WG_	241	0	0
RYW	WG_	255	0	0
RYW	WG_	873	0	0
RYW	Q	278	0	0
RYW	Q	1008	0	0
RYW	YWW	523	0	0
RYW	YWW	529	0	0
WG_	_G_	12	0	0
WG_	_G_	36	0	0
WG_	_G_	141	0	0
WG_	_G_	248	0	0
WG_	_G_	609	0	0
WG_	_G_	636	0	0
WG_	RYW	244	0	0
WG_	RYW	256	0	0
WG_	RYW	870	0	0
WG_	YGG	749	0	0
WG_	YGG	846	0	0
WG_	YWW	439	0	0
WGG	YWW	304	0	0
WGG	YWW	317	0	0
WGY	_G_	843	0	0
WGY	_G_	970	0	0
WGY	WWR	129	0	0

WGY	YWW	270	0	0
WRG	___	365	0	0
WRG	WGY	1115	0	0
Q	_G_	1033	0	0
Q	RYW	278	0	0
Q	RYW	1008	0	0
Q	RYW	1047	0	0
WWR	___	183	0	0
WWR	___	314	0	0
WWR	___	472	0	0
WWR	___	650	0	0
WWR	___	835	0	0
WWR	WGY	118	0	0
WWR	WGY	125	0	0
WWR	WGY	275	0	0
WWR	WRG	158	0	0
YGG	_G_	710	0	0
YGG	RYW	775	0	0
YGG	WG_	749	0	0
YGG	Q	515	0	0
YWW	___	38	0	0
YWW	_G_	406	0	0
YWW	_G_	470	0	0
YWW	RYW	523	0	0
YWW	RYW	531	0	0
YWW	WG_	448	0	0
YWW	WGG	301	0	0
YWW	WGG	316	0	0
YWW	WGY	83	0	0
YWW	WGY	269	0	0

Antlist	InitialPosX	InitialPosY	ColonyArea	AntLength	MinX	MinY	MaxX	MaxY
___	NA	NA	325637	75.5	24	36	768	539
_G_	NA	NA						
RYW	NA	NA						
WG_	NA	NA						
WGG	NA	NA						
WGY	NA	NA						
Q	NA	NA						
WWR	NA	NA						
YGG	NA	NA						
YWW	NA	NA						
WRG	NA	NA						

Actor	Target	Time	ActorPosX	ActorPosY
Q	YGG	1110	0	0
Q	YGG	1124	0	0
Q	YGG	1166	0	0
Q	YGG	1281	0	0
Q	RYW	1283	0	0
Q	YGG	1318	0	0
Q	YGG	1377	0	0
YGG	___	51	0	0
YGG	Q	1110	0	0
YGG	Q	1125	0	0
YGG	Q	1170	0	0
YGG	RYW	1201	0	0
YGG	Q	1335	0	0
YGG	Q	1382	0	0
YGG	WG_	1425	0	0
___	YGG	2	0	0
___	WG_	5	0	0
___	Q	33	0	0
___	RYW	90	0	0
___	RYW	121	0	0
___	Q	1152	0	0
___	WG_	1324	0	0
WG_	YGG	5	0	0
WG_	WWR	90	0	0
WG_	RYW	95	0	0
WG_	RYW	106	0	0
WG_	WWR	299	0	0
WG_	RYW	428	0	0
WG_	WWR	433	0	0
WG_	WWR	442	0	0
WG_	WWR	457	0	0
WG_	WWR	475	0	0
WG_	RYW	527	0	0
WG_	WWR	576	0	0
WG_	RYW	1214	0	0
WG_	WWR	1244	0	0
WG_	RYW	1283	0	0
WG_	RYW	1297	0	0
WG_	RYW	1300	0	0
WG_	___	1310	0	0
WG_	Q	1328	0	0
WG_	Q	1343	0	0
WG_	RYW	1353	0	0
WG_	WWR	1375	0	0
WG_	RYW	1391	0	0
WG_	WWR	1397	0	0
WG_	WWR	1412	0	0
WG_	Q	1420	0	0

WG_	YGG	1424	0	0
WWR	WG_	8	0	0
WWR	RYW	26	0	0
WWR	WG_	90	0	0
WWR	WG_	100	0	0
WWR	RYW	107	0	0
WWR	WG_	120	0	0
WWR	WG_	296	0	0
WWR	WG_	300	0	0
WWR	WG_	431	0	0
WWR	WG_	440	0	0
WWR	WG_	444	0	0
WWR	WG_	453	0	0
WWR	WG_	459	0	0
WWR	WG_	577	0	0
WWR	RYW	1227	0	0
WWR	RYW	1230	0	0
WWR	WG_	1244	0	0
WWR	RYW	1293	0	0
WWR	RYW	1323	0	0
WWR	RYW	1326	0	0
WWR	RYW	1350	0	0
WWR	RYW	1366	0	0
WWR	RYW	1380	0	0
WWR	RYW	1396	0	0
RYW	WG_	5	0	0
RYW	WG_	25	0	0
RYW	___	32	0	0
RYW	___	84	0	0
RYW	WG_	95	0	0
RYW	WG_	105	0	0
RYW	WWR	108	0	0
RYW	___	117	0	0
RYW	___	143	0	0
RYW	WG_	235	0	0
RYW	WG_	435	0	0
RYW	WWR	440	0	0
RYW	YGG	452	0	0
RYW	WG_	526	0	0
RYW	YGG	1200	0	0
RYW	YGG	1204	0	0
RYW	WG_	1214	0	0
RYW	WWR	1225	0	0
RYW	WWR	1230	0	0
RYW	WWR	1250	0	0
RYW	WWR	1271	0	0
RYW	WWR	1275	0	0
RYW	WG_	1282	0	0
RYW	WWR	1313	0	0

RYW	WWR	1322	0	0
RYW	WWR	1325	0	0
RYW	WWR	1327	0	0
RYW	WWR	1348	0	0
RYW	WWR	1365	0	0
RYW	WG_	1391	0	0
RYW	WWR	1395	0	0

Antlist	InitialPosX	InitialPosY	ColonyArea	AntLength	MinX	MinY	MaxX	MaxY
Q	NA	NA	110432	78.1	80	0	598	386
WG_	NA	NA						
RYW	NA	NA						
YGG	NA	NA						
WWR	NA	NA						
___	NA	NA						

Actor	Target	ActorPosX	ActorPosY	Time
RWY_	WWYW	190	253	1186
RWY_	WWYW	155	281	1188
RWY_	_WWR	174	246	1190
RWY_	WWYW	106	288	1210
RWY_	WWYW	112	264	1277
RWY_	____(3)	228	234	1327
RWY_	_BWW	381	302	1390
RWY_	____(1)	379	267	1418
RWY_	____(3)	284	242	1431
RWY_	GG_W	382	287	1440
RWY_	WWGW	378	310	1446
RWY_	____(1)	395	285	1452
RWY_	WWGW	381	281	1457
RWY_	YWG_	337	369	1465
RWY_	____(3)	323	377	1480
RWY_	_BWW	336	355	1509
RWY_	_BWW	446	335	1864
RWY_	YYWW	446	335	1866
G___	RWY_	100	301	1
G___	WWYW	115	332	1251
_GBB	____(3)	103	410	288
_GBB	RWY_	101	346	315
_GBB	G___	110	308	353
_GBB	____(1)	160	415	398
_GBB	____(1)	190	496	428
_GBB	YWG_	267	496	441
_GBB	__B_	409	223	455
_GBB	WYWG	432	229	478
_GBB	____(2)	452	274	493
_GBB	____(3)	422	268	506
_GBB	____(1)	327	341	517
_GBB	WWYG	373	378	689
_GBB	YWG_	318	396	822
_GBB	____(3)	312	300	842
_GBB	__B_	427	215	904
_GBB	BBRR	249	312	968
_GBB	WWYW	262	338	975
_GBB	YYWW	345	339	996
_GBB	BBRR	369	305	1002
_GBB	YYWW	416	323	1007
_GBB	WWYW	432	279	1018
_GBB	____(2)	549	287	1023
_GBB	WW__	615	287	1033
_GBB	WWYG	656	218	1045
_GBB	WYBB	750	419	1105
_GBB	WWGR (Q)	816	393	1110
_GBB	WW__	872	334	1115
_GBB	WWYG	707	239	1129

_GBB	____(1)	646	250	1145
_GBB	WYWG	701	229	1222
_GBB	____(3)	535	228	1400
_GBB	WYWG	533	212	1427
_GBB	__G_	533	212	1430
_GBB	YYWW	548	248	1442
_GBB	WWRB	541	207	1658
_GBB	_WWR	668	290	1683
_GBB	____(2)	656	279	1696
_GBB	WBYR	745	224	1852
WWYW	WGGR	181	334	68
WWYW	WGGR	315	313	180
WWYW	_BWW	315	313	181
WWYW	WGGR	161	344	345
WWYW	WGGR	237	340	511
WWYW	WGGR	192	329	552
WWYW	YYWW	260	330	573
WWYW	_WWR	561	331	600
WWYW	WWGB (Q)	622	310	610
WWYW	WWRB	622	310	610
WWYW	BBRR	648	483	634
WWYW	YWWB	648	385	635
WWYW	WWGB (Q)	648	385	636
WWYW	WWYG	642	301	644
WWYW	WWGR (Q)	681	234	649
WWYW	WWYG	650	287	652
WWYW	_WWR	612	335	654
WWYW	WGRY	598	332	750
WWYW	WYWG	598	257	754
WWYW	GG_W	318	309	793
WWYW	WGGR	175	243	828
WWYW	RWY_	137	299	870
WWYW	YYWW	231	470	883
WWYW	WGGR	231	470	885
WWYW	BBRR	236	621	968
WWYW	YWG_	318	390	1027
WWYW	_WW_	304	411	1091
WWYW	BBRR	484	355	1559
WWYW	GG_W	498	307	1562
WWYW	____(1)	461	301	1568
WWYW	_BWW	477	317	1616
WWYW	BBRR	452	333	1620
WWYW	RWY_	452	333	1620
WWYW	WWGW	419	300	1627
WWYW	YWG_	329	405	1634
WWYW	BBRR	139	322	1852
____(1)	WWYW	236	337	228
____(1)	WGGR	226	340	233
____(1)	_GBB	174	412	404

____(1)	WGGR	225	353	431
____(1)	WWYW	197	355	443
____(1)	____(3)	330	357	515
____(1)	WYWG	526	273	590
____(1)	_WWR	547	289	592
____(1)	WBYR	698	244	608
____(1)	WW__	774	232	625
____(1)	__YY	705	216	704
____(1)	WWYG	651	231	748
____(1)	_RYY	646	223	755
____(1)	WWYW	593	294	840
____(1)	____(3)	576	244	854
____(1)	WYWG	602	257	939
____(1)	____(3)	607	258	964
____(1)	WWRB	611	256	983
____(1)	WGGR	623	244	1132
____(1)	BBRR	497	306	1212
____(1)	____(2)	434	234	1499
____(1)	WWYG	444	262	1516
____(1)	_BWW	460	257	1524
____(1)	WWYW	429	274	1567
____(1)	WWRB	439	226	1660
WGGR	WWYW	168	369	75
WGGR	WWYW	272	306	187
WGGR	_BWW	356	271	209
WGGR	_BWW	318	314	223
WGGR	WWYW	280	300	226
WGGR	____(1)	224	323	235
WGGR	WWYW	191	334	249
WGGR	WWYW	209	331	545
WGGR	YYWW	278	282	570
WGGR	YYWW	224	339	653
WGGR	YYWW	270	345	898
WGGR	WWRB	339	292	1056
WGGR	RWY_	193	242	1204
WGGR	__YY	244	229	1228
WGGR	_BWW	385	234	1244
WGGR	____(1)	388	287	1248
WGGR	GG_W	343	343	1259
WGGR	____(1)	421	308	1271
WGGR	WYWG	742	232	1287
WGGR	WBYR	783	248	1329
WGGR	WW__	705	238	1326
WGGR	WWYG	638	262	1331
WGGR	WGRY	584	320	1341
WGGR	____(1)	463	309	1348
WGGR	____(3)	238	240	1365
WGGR	RWY_	291	255	1415
WGGR	WWRB	636	247	1440

WGGR	_BWW	478	288	1466
WGGR	____(1)	465	292	1468
WGGR	GG_W	371	309	1479
WGGR	WWYW	224	345	1742
WGGR	YWG_	317	446	1750
WGGR	WW__	334	447	1751
WGGR	RWY_	417	311	1757
WGGR	BBRR	424	319	1759
_BWW	WGGR	312	322	223
_BWW	WWRB	361	301	241
_BWW	_RYY	341	294	247
_BWW	_RYY	311	257	444
_BWW	YYWW	348	340	474
_BWW	____(3)	405	223	508
_BWW	____(3)	357	310	522
_BWW	_RYY	368	242	698
_BWW	WWYW	323	253	793
_BWW	_RYY	331	240	820
_BWW	____(1)	552	278	909
_BWW	____(2)	466	278	920
_BWW	RWY_	102	254	989
_BWW	G___	102	254	990
_BWW	_WW_	412	230	1019
_BWW	WWRB	469	261	1034
_BWW	WWRB	384	267	1060
_BWW	____(2)	517	306	1084
_BWW	WWRB	635	219	1134
_BWW	WYWG	673	248	1146
_BWW	____(1)	556	286	1168
_BWW	____(1)	441	253	1304
_BWW	WYWG	443	245	1316
_BWW	RWY_	401	235	1365
_BWW	WWGW	485	305	1377
_BWW	YYWW	488	301	1427
_BWW	____(1)	471	289	1432
_BWW	WGGR	346	319	1490
_BWW	____(1)	396	294	1496
_BWW	WWYG	417	306	1518
_BWW	YYWW	417	306	1520
_BWW	____(1)	460	274	1532
_BWW	WBYR	743	227	1561
_BWW	_RRR	767	257	1583
_BWW	GG_W	532	300	1614
_BWW	WW__	544	278	1621
_BWW	WGRY	574	331	1656
_BWW	WW__	401	242	1739
_BWW	_WWR	593	255	1757
_BWW	____(3)	528	249	1762
_BWW	RWY_	428	321	1852

_BWW	__B_	456	242	1884
YWG_	____(1)	369	408	557
YWG_	_WWR	378	419	1365
YWG_	GG_W	378	419	1365
YWG_	RWY_	367	406	1494
YWG_	WW__	343	413	1834
WGRY	WW__	397	363	196
WGRY	GG_W	397	363	229
WGRY	WW__	403	403	326
WGRY	WWYG	443	375	351
WGRY	GG_W	472	405	463
WGRY	WWYG	469	394	565
WGRY	_WWR	546	372	687
WGRY	WWGB (Q)	639	334	755
WGRY	____(2)	657	291	986
WGRY	____(1)	662	285	1001
WWRB	WW__	428	297	128
WWRB	____(2)	482	314	260
WWRB	WYWG	437	282	303
WWRB	_RYY	354	257	418
WWRB	____(1)	422	304	452
WWRB	__YY	409	325	761
WWRB	____(1)	580	263	986
WWRB	WBYR	590	247	1000
WWRB	BBRR	485	292	1021
WWRB	WGGR	350	262	1059
WWRB	WWGW	459	306	1113
WWRB	____(1)	605	262	1136
WWRB	WBYR	689	285	1194
WWRB	__B_	581	219	1206
WWRB	_BWW	593	244	1214
WWRB	WYWG	711	282	1224
WWRB	WWGR (Q)	724	297	1230
WWRB	WWYG	614	282	1271
WWRB	WBYR	652	258	1408
WWRB	GG_W	607	275	1525
WWRB	____(1)	485	248	1609
WWRB	____(3)	517	198	1654
WWRB	____(1)	461	238	1726
_WWR	____(1)	513	326	20
_WWR	YYWW	576	302	57
_WWR	GG_W	539	320	162
_WWR	WWYW	594	342	619
_WWR	WGRY	555	355	690
_WWR	WWGR (Q)	679	303	735
_WWR	____(1)	674	254	759
_WWR	WBYR	709	235	857
_WWR	WW__	697	262	917
_WWR	WWYG	681	246	947

_WWR	____(1)	617	241	983
_WWR	GG_W	328	355	1148
_WWR	_WW_	286	340	1158
_WWR	G___	127	341	1217
_WWR	WWYW	148	338	1280
_WWR	__YY	244	388	1315
_WWR	_WW_	335	328	1328
_WWR	YYWW	438	408	1374
_WWR	BBRR	507	405	1442
_WWR	WBYR	722	260	1533
_WWR	WWGB (Q)	669	362	1636
_WWR	__YY	658	309	1866
WYWG	__B_	461	220	239
WYWG	_GBB	431	223	475
WYWG	____(2)	504	217	507
WYWG	WWGW	511	245	522
WYWG	____(3)	564	234	570
WYWG	__B_	481	228	759
WYWG	____(1)	621	221	775
WYWG	____(1)	619	263	1079
WYWG	WWRB	571	244	1088
WYWG	_BWW	657	288	1150
WYWG	____(1)	591	287	1160
WYWG	WW__	593	213	1191
WYWG	__B_	687	221	1218
WYWG	WBYR	685	302	1234
WYWG	_BWW	478	263	1312
WYWG	____(1)	469	270	1317
WYWG	WWRB	497	233	1452
WW__	WGRY	376	346	240
WW__	GG_W	474	388	331
WW__	WWYG	493	403	337
WW__	WGRY	460	390	353
WW__	____(3)	691	283	448
WW__	____(1)	791	238	627
WW__	_WW_	822	349	732
WW__	WWGB (Q)	729	363	767
WW__	_RRR	724	335	779
WW__	WBYR	724	240	814
WW__	____(1)	611	279	983
WW__	WWYG	694	256	1081
WW__	WWRB	685	242	1162
WW__	____(3)	714	227	1195
WW__	WBYR	700	271	1273
WW__	_RRR	712	275	1447
WW__	WGGR	732	216	1463
WW__	_WWR	590	280	1495
WW__	____(2)	612	275	1614
WW__	WGGR	546	265	1633

WW__	____(1)	440	260	1661
WW__	RWY_	416	313	1678
WWGW	_G__	522	222	270
WWGW	YYWW	346	256	540
WWGW	WGGR	444	282	555
WWGW	____(1)	444	282	564
WWGW	WWYW	547	291	810
WWGW	____(1)	599	272	965
WWGW	____(1)	511	241	1116
WWGW	__YY	418	298	1162
WWGW	WBYR	698	298	1251
WWGW	____(1)	464	303	1272
WWGW	GG_W	420	314	1445
WWGW	__YY	214	400	1519
WWGW	GG_W	503	279	1551
WWGW	BBRR	530	336	1559
WWGW	RWY_	410	309	1578
WWGW	YYWW	382	378	1618
WWGW	____(1)	401	295	1629
WWGW	__B_	412	212	1659
WWGW	WWYG	373	241	1671
WWGW	__G_	370	223	1716
__B_	WYWG	478	213	762
__B_	____(1)	592	242	966
__B_	____(1)	638	227	1099
__B_	_BWW	660	279	1161
__B_	____(1)	444	243	1460
__B_	____(1)	475	225	1878
WBYR	_RYY	634	279	2
WBYR	_RYY	707	225	45
WBYR	_RYY	713	218	127
WBYR	____(3)	713	218	174
WBYR	WW__	733	230	816
WBYR	____(2)	724	243	933
WBYR	WWYG	711	240	1046
WBYR	WW__	705	254	1049
WBYR	_RRR	693	314	1057
WBYR	____(1)	643	284	1065
WBYR	_WW_	691	312	1256
WBYR	_RRR	739	224	1512
_RYY	__G_	592	282	16
_RYY	____(3)	646	229	118
_RYY	WBYR	694	227	125
_RYY	YYWW	590	294	176
_RYY	WWYG	585	323	181
_RYY	GG_W	585	323	186
_RYY	__G_	555	242	209
_RYY	____(1)	209	324	272
_RYY	_WW_	400	386	321

_RYY	____(1)	417	320	383
_RYY	YYWW	359	250	507
_RYY	__B_	416	211	760
_RYY	____(3)	383	223	783
_RYY	_BWW	444	281	924
_RYY	WW__	583	287	1000
_RYY	____(1)	622	238	1030
_RYY	____(2)	565	258	1049
_RYY	WWRB	535	245	1082
_RYY	WW__	519	229	1178
_RYY	_G__	501	231	1210
_RRR	WBYR	712	291	19
_RRR	GG_W	712	288	27
_RRR	WWGR (Q)	710	298	175
_RRR	WBYR	710	296	282
_RRR	WW_W	704	313	299
_RRR	WWGB (Q)	683	347	476
_RRR	WW_W	696	324	585
_RRR	WWGR (Q)	703	319	595
_RRR	WW_W	703	326	710
_RRR	WWYG	718	296	997
_RRR	WW_W	729	311	1006
_RRR	WWGB (Q)	688	366	1027
_RRR	WBYR	696	349	1160
_RRR	WYBB	696	349	1161
_RRR	WWGB (Q)	696	349	1163
_RRR	WGRY	673	348	1380
_RRR	WWGB (Q)	723	339	1389
_RRR	WBYR	682	278	1455
_RRR	WW__	770	244	1467
_RRR	WWGR (Q)	802	275	1550
_RRR	WWGB (Q)	726	321	1609
GG_W	WWGR (Q)	808	406	5
GG_W	_RRR	736	229	25
GG_W	WBYR	730	253	29
GG_W	WW__	656	285	38
GG_W	YYWW	597	292	48
GG_W	__G_	611	277	51
GG_W	WWYG	602	303	54
GG_W	_WWR	594	309	74
GG_W	WWYG	604	320	103
GG_W	WWGW	548	288	158
GG_W	_WWR	551	313	162
GG_W	WGRY	422	382	227
GG_W	WWYG	478	410	317
GG_W	____(1)	112	246	551
GG_W	WWGW	406	284	651
GG_W	_RYY	395	235	654
GG_W	WWYG	521	298	668

GG_W	__YY	516	282	671
GG_W	WYWG	527	273	708
GG_W	__G_	593	276	716
GG_W	_RYY	593	276	717
GG_W	WWYG	533	293	735
GG_W	_GBB	328	328	755
GG_W	YYWW	331	386	896
GG_W	WGGR	352	356	910
GG_W	__YY	359	328	925
GG_W	_WW_	374	364	1316
GG_W	_WWR	320	379	1326
GG_W	YWG_	320	393	1339
GG_W	RWY_	388	335	1426
GG_W	____(1)	419	308	1433
GG_W	WW__	396	253	1481
GG_W	_BWW	414	323	1500
GG_W	WW__	598	297	1517
GG_W	WWRB	600	292	1522
GG_W	WW__	603	301	1532
GG_W	____(1)	487	264	1555
GG_W	WWYW	505	291	1580
GG_W	BBRR	507	309	1595
GG_W	WWYW	481	277	1602
GG_W	____(1)	471	274	1607
GG_W	BBRR	518	315	1623
GG_W	_RYY	432	248	1799
GG_W	_BWW	358	258	1813
GG_W	__YY	532	331	1826
WWGR (Q)	GG_W	808	392	6
WWGR (Q)	WYBB	759	389	21
WWGR (Q)	GG_W	759	328	25
WWGR (Q)	_RRR	753	327	30
WWGR (Q)	_RRR	746	339	308
WWGR (Q)	_WW_	779	391	441
WWGR (Q)	_RRR	754	314	455
WWGR (Q)	WYBB	727	330	465
WWGR (Q)	WWGB (Q)	722	333	472
WWGR (Q)	WW_W	696	330	525
WWGR (Q)	____(1)	694	291	615
WWGR (Q)	WWYG	692	287	639
WWGR (Q)	_WWR	689	387	825
WWGR (Q)	WW_W	686	307	890
WWGR (Q)	_WW_	682	295	929
WWGR (Q)	WW_W	750	368	1030
WWGR (Q)	_RRR	720	338	1039
WWGR (Q)	WW__	712	302	1079
WWGR (Q)	_RRR	716	323	1089
WWGR (Q)	_GBB	778	382	1107
WWGR (Q)	WWRB	749	317	1234

WWGR (Q)	_RRR	756	314	1445
WWGR (Q)	WWGB (Q)	733	354	1718
WWGR (Q)	WBYR	750	276	1738
WWGR (Q)	WYBB	852	313	1754
WWGR (Q)	WBYR	783	269	1781
WYBB	WWGB (Q)	721	371	470
WYBB	_RRR	736	367	736
WYBB	WWGB (Q)	735	360	740
WYBB	WW_W	710	400	1029
WYBB	WWGB (Q)	700	413	1037
WYBB	WW_W	702	375	1058
WYBB	_RRR	714	390	1216
WYBB	WWGB (Q)	703	400	1300
WYBB	WW_W	725	408	1434
WYBB	YWWB	667	404	1470
WYBB	WW_W	742	407	1495
WYBB	WWGR (Q)	826	359	1549
BBRR	__YY	594	428	2
BBRR	YWWB	652	416	635
BBRR	WYBB	687	416	673
BBRR	_WW_	742	360	688
BBRR	WW_W	679	318	702
BBRR	WGRY	565	330	711
BBRR	WWYG	505	296	724
BBRR	____(1)	465	325	730
BBRR	RWY_	153	259	831
BBRR	G___	153	259	834
BBRR	__YY	375	301	988
BBRR	WWRB	485	308	1025
BBRR	WGRY	538	365	1084
BBRR	YYWW	470	364	1197
BBRR	WWGW	508	386	1397
BBRR	YYWW	488	381	1421
BBRR	GG_W	474	335	1728
BBRR	RWY_	474	335	1731
BBRR	WWGW	460	314	1758
BBRR	WGGR	383	312	1765
BBRR	G___	134	258	1842
__YY	YWWB	660	419	361
__YY	WYBB	692	407	606
__YY	BBRR	681	407	634
__YY	WWYG	636	325	650
__YY	_WWR	601	328	654
__YY	__G_	555	290	660
__YY	____(1)	683	229	708
__YY	GG_W	580	294	724
__YY	WWYG	571	309	730
__YY	GG_W	368	330	922
__YY	WWGW	397	308	1160

__YY	GG_W	346	341	1204
__YY	_WW_	318	300	1220
__YY	WGGR	265	239	1228
__YY	YWG_	320	400	1244
__YY	WWYW	235	300	1765
__YY	_WW_	235	300	1767
__YY	RWY_	464	323	1775
__YY	_WWR	639	291	1866
WW_W	_RRR	695	336	1003
WW_W	WWGB (Q)	695	336	1005
WW_W	WYBB	726	380	1145
WW_W	_RRR	726	380	1205
_WW_	YYWW	628	362	249
_WW_	YWWB	673	384	332
_WW_	____(2)	649	265	371
_WW_	WBYR	710	280	388
_WW_	_RRR	727	330	396
_WW_	_G__	774	390	420
_WW_	WWGR (Q)	770	364	442
_WW_	_RRR	730	316	698
_WW_	WW_W	760	326	773
_WW_	WWGB (Q)	680	326	833
_WW_	WGRY	600	331	910
_WW_	WW__	697	268	924
_WW_	BBRR	445	282	1015
_WW_	RWY_	133	312	1036
_WW_	WWYW	263	347	1096
_WW_	_WWR	269	309	1169
_WW_	GG_W	287	339	1206
_WW_	__YY	317	311	1217
_WW_	BBRR	515	311	1240
_WW_	WGRY	592	321	1248
_WW_	WBYR	649	332	1256
_WW_	_RRR	676	322	1271
_WW_	GG_W	359	354	1319
_WW_	____(1)	413	315	1350
_WW_	YWG_	285	386	1371
_WW_	WWYW	241	327	1443
_WW_	WGGR	336	318	1637
_WW_	WWYW	336	321	1640
_WW_	RWY_	386	331	1656
_WW_	YWG_	331	393	1665
_WW_	RWY_	385	348	1841
YWWB	__YY	660	403	552
YWWB	BBRR	658	396	611
YWWB	WWGB (Q)	668	386	712
YWWB	WGRY	673	347	996
YWWB	WYBB	667	400	1016
YWWB	WWGB (Q)	674	404	1029

YWWB	WYBB	655	417	1436
YWWB	WWGB (Q)	675	390	1511
WWYG	GG_W	563	329	177
WWYG	____(3)	563	329	180
WWYG	GG_W	484	368	270
WWYG	WGRY	487	374	575
WWYG	_WWR	528	313	622
WWYG	WWGR (Q)	653	275	631
WWYG	WBYR	693	254	642
WWYG	WW_W	673	298	645
WWYG	_WWR	570	310	652
WWYG	WGRY	472	368	662
WWYG	__YY	562	297	727
WWYG	WBYR	697	275	745
WWYG	WWGR (Q)	783	274	753
WWYG	_WW_	804	312	788
WWYG	BBRR	774	342	793
WWYG	WWGR (Q)	772	292	841
WWYG	WBYR	762	249	852
WWYG	____(1)	675	235	862
WWYG	WWGW	522	281	1233
WWYG	WWRB	292	299	1273
WWYG	WBYR	647	310	1314
WWYG	WGGR	629	270	1336
WWYG	WWRB	680	247	1408
WWYG	YYWW	600	290	1440
WWYG	____(1)	454	286	1480
__G_	_RYY	567	231	211
__G_	WWGW	565	232	244
__G_	____(2)	523	372	259
__G_	_GBB	592	251	294
__G_	WWGW	523	248	305
__G_	____(2)	500	250	504
__G_	WYWG	525	250	506
__G_	WWGW	525	260	520
__G_	WYWG	575	236	638
__G_	____(3)	583	288	717
__G_	____(1)	613	239	937
__G_	WWYG	625	245	941
__G_	_RYY	420	222	955
__G_	____(1)	555	245	974
__G_	____(3)	555	245	976
__G_	WW__	560	240	977
__G_	_WWR	620	218	990
__G_	____(1)	680	270	998
__G_	WWRB	560	290	1020
__G_	WWGW	525	175	1057
YYWW	__G_	565	285	5
YYWW	WWYG	557	312	11

YYWW	____(3)	611	251	22
YYWW	_RYY	604	270	25
YYWW	WWYG	550	290	44
YYWW	GG_W	608	309	160
YYWW	_WW_	611	335	248
YYWW	WWGB (Q)	636	339	289
YYWW	WWYW	280	308	576
YYWW	WGGR	262	298	580
YYWW	GG_W	270	400	892
YYWW	WWYG	580	290	1442
YYWW	_BWW	533	303	1464
YYWW	BBRR	484	358	1476
YYWW	GG_W	434	344	1496
YYWW	BBRR	484	345	1525
YYWW	RWY_	450	373	1549
YYWW	_WWR	577	290	1765
YYWW	WWGR (Q)	744	295	1786
YYWW	WYBB	789	372	1801
YYWW	WWGB (Q)	714	344	1820
YYWW	RWY_	435	317	1918
____(2)	WW__	505	289	31
____(2)	YYWW	525	284	47
____(2)	WWGW	535	374	56
____(2)	_WWR	530	307	249
____(2)	WWRB	506	296	278
____(2)	_BWW	377	306	314
____(2)	__G_	533	395	956
____(2)	____(1)	684	282	967
____(2)	WWYG	651	241	973
____(2)	WGRY	606	302	1011
____(2)	__G_	617	302	1016
____(2)	WWRB	543	293	1031
____(2)	____(3)	546	269	1102
____(2)	BBRR	523	313	1120
____(2)	____(1)	472	289	1232
____(2)	_BWW	462	283	1251
____(2)	____(3)	293	223	1320
____(2)	G___	115	207	1387
____(2)	GG_W	547	286	1517
____(2)	WW__	622	310	1550
____(2)	GG_W	568	307	1675
____(2)	_WWR	638	294	1686
____(2)	_RRR	638	294	1690
____(2)	_GBB	641	280	1694
____(2)	WWRB	498	262	1825
____(2)	WWYG	398	234	1859
____(3)	WBYR	691	212	29
____(3)	YYWW	617	238	162
____(3)	_GBB	616	224	276

____(3)	WBYR	664	246	363
____(3)	_WW_	664	246	373
____(3)	WYWG	584	221	578
____(3)	____(1)	642	241	613
____(3)	WWYG	690	238	644
____(3)	____(1)	647	241	652
____(3)	__G_	586	218	664
____(3)	____(1)	637	211	759
____(3)	_RYY	410	222	780
____(3)	_GBB	410	222	783
____(3)	____(1)	595	241	966
____(3)	WYWG	515	233	999
____(3)	____(1)	630	231	1097
____(3)	WWYG	660	215	1110
____(3)	_BWW	678	267	1155
____(3)	____(1)	439	246	1470

Antlist	InitialPosX	InitialPosY	ColonyArea	AntLength	MinX	MinY	MaxX	MaxY
RWY_	104	282	191259	63.4	58	0	899	390
G___	100	301						
_GBB	114	419						
WGGR	223	256						
WWYW	230	315						
_BWW	302	270						
YWG_	304	422						
WGRY	401	362						
WWRB	409	294						
WYWG	476	223						
WW__	475	269						
__B_	457	212						
____(1)	185	408						
____(2)	520	299						
WWGW	528	235						
YYWW	565	286						
__G_	561	228						
WWYG	573	315						
BBRR	597	421						
__YY	625	401						
_WW_	634	361						
____(3)	631	246						
_RYY	643	274						
WBYR	666	235						
WW_W	681	319						
WWGB (Q)	657	337						
YWWB	659	406						
WYBB	731	363						
_RRR	714	287						
WWGR (Q)	753	337						
GG_W	777	482						
_WWR	455	307						
_G__	0	0						

Actor	Target	ActorPosX	ActorPosY	Time
WGGR	__YY	194	253	27
WGGR	__YY	174	257	59
WGGR	YYWW	185	254	247
WGGR	__YY	219	238	425
WGGR	_WWR	265	260	492
WGGR	WWYW	237	283	510
WGGR	YWRG	230	336	531
WGGR	WWGR (Q)	254	338	534
WGGR	_WWR	254	338	536
WGGR	_WWR	211	292	582
WGGR	WGRY	324	393	686
WGGR	YWWB	457	366	810
WGGR	____(4)	422	318	897
WGGR	____(1)	408	328	902
WGGR	____(4)	422	316	1217
WGGR	GG_W	421	300	1241
WGGR	WWRG	417	297	1247
WGGR	____(1)	403	341	1257
WGGR	YWWB	400	356	1266
WGGR	WGRY	400	356	1270
WGGR	____(4)	420	280	1288
WGGR	____(1)	420	280	1340
WGGR	_WWR	268	262	1361
WGGR	GG_W	194	211	1372
WGGR	YYWW	134	324	1381
YYWW	__YY	237	317	31
YYWW	WWYW	215	312	99
YYWW	WGGR	172	304	118
YYWW	YWRG	173	335	132
YYWW	_WWR	241	284	244
YYWW	YWRG	174	354	290
YYWW	__YY	151	357	378
YYWW	WWYW	167	317	1402
YWRG	WGGR	213	326	572
YWRG	YYWW	212	342	828
YWRG	_WWR	254	324	930
YWRG	YYWW	169	364	980
WWGR (Q)	____(1)	333	319	1264
WWGR (Q)	WGRY	356	361	1329
WWGR (Q)	____(1)	385	342	1336
WWGR (Q)	WYBB	450	304	1346
WWGR (Q)	_WW_	468	374	1389
WWGR (Q)	____(6)	480	324	1586
____(1)	WWGR (Q)	335	316	1273
____(1)	WGRY	393	353	1324
WGRY	WWYW	346	384	402
WGRY	WGGR	345	383	655
WGRY	____(2)	345	383	748

WGRY	WWGR (Q)	345	384	1324
____(2)	WGRY	338	394	762
____(3)	_RYY	421	225	123
____(3)	__YY	420	230	331
____(3)	GG_W	453	221	447
____(3)	WWRG	451	246	523
____(3)	WYBB	463	233	782
____(3)	WWRG	473	233	871
____(3)	__YY	473	224	973
____(3)	GG_W	466	223	1200
____(3)	WWRG	431	221	1205
____(3)	GG_W	453	229	1211
____(3)	_WW_	518	380	1261
____(3)	_GB_	781	228	1320
____(3)	RWY_	702	282	1324
____(3)	_GB_	822	230	1336
____(3)	YWWB	556	352	1359
____(3)	WY__	488	300	1417
YWWB	WYBB	469	296	16
YWWB	WYBB	456	271	205
YWWB	WWGR (Q)	477	336	1345
YWWB	____(4)	542	317	1357
YWWB	WWGR (Q)	552	319	1381
WYBB	YWWB	469	303	2
WYBB	____(4)	498	289	27
WYBB	WWRG	477	259	101
WYBB	____(4)	469	263	1227
WYBB	GG_W	460	274	1240
WYBB	YWWB	448	320	1258
WYBB	WGGR	440	339	1260
WYBB	_WW_	474	319	1274
WYBB	____(6)	605	332	1340
WYBB	____(6)	597	343	1439
_WW_	__YY	476	402	13
_WW_	_G__	476	402	323
_WW_	WWYW	476	402	378
_WW_	YWWB	527	384	1358
_RYY	_GB_	500	176	99
_RYY	WY__	472	200	128
_RYY	_GB_	485	200	159
_RYY	__YY	147	198	202
_RYY	WY__	503	200	223
_RYY	white legs	495	176	230
_RYY	_BWW	516	165	965
_RYY	WWRG	528	173	1430
_RYY	_BWW	520	165	1435
_RYY	WWRG	543	196	1447
_RYY	_BWW	525	182	1452
_RYY	_G__	526	132	1530

_RYY	__YY	500	146	1546
_RYY	__YY	497	231	1572
_RYY	_BWW	490	213	1577
_RYY	WY__	459	200	1580
WWRG	_WWR	549	285	5
WWRG	WW_W	549	285	6
WWRG	__YY	523	252	92
WWRG	WYBB	494	246	102
WWRG	RWY_	638	214	139
WWRG	____(6)	846	203	170
WWRG	_G__	846	215	194
WWRG	BBRR	806	297	227
WWRG	_RRR	627	273	240
WWRG	WY__	594	227	243
WWRG	WWYW	572	227	258
WWRG	RWY_	651	208	293
WWRG	WY__	600	208	378
WWRG	____(4)	549	245	402
WWRG	GG_W	494	204	532
WWRG	__YY	434	221	627
WWRG	WWBG	471	245	885
WWRG	__YY	478	216	990
WWRG	GG_W	452	222	1199
WWRG	GG_W	400	245	1220
WWRG	_GB_	548	226	1269
WWRG	_RRR	543	246	1295
WWRG	WY__	516	210	1358
WWRG	_RYY	538	206	1490
____(4)	WY__	506	236	114
____(4)	white legs	511	246	141
____(4)	_G__	501	299	354
____(4)	WYBB	501	296	424
____(4)	__YY	380	234	517
____(4)	____(1)	393	298	549
____(4)	WWRG	356	248	1248
____(4)	_RRR	540	255	1267
____(4)	YWWB	495	315	1277
____(4)	WGGR	440	271	1289
____(4)	YWWB	509	344	1317
____(4)	_GB_	752	203	1332
____(4)	RWY_	735	265	1386
____(4)	____(5)	660	315	1414
____(4)	WWGR (Q)	467	303	1603
____(4)	____(1)	377	300	1626
____(4)	WWGR (Q)	415	338	1640
____(4)	_WW_	495	385	1698
____(4)	WWGR (Q)	413	375	1707
_WWR	WWYW	550	346	20
_WWR	WW_W	600	320	95

_WWR	__YY	605	380	101
_WWR	_G__	610	377	125
_WWR	WWYW	535	320	182
_WWR	WGGR	252	297	507
WWYW	_WWR	561	345	25
WWYW	WYBB	490	290	67
WWYW	WGRY	360	350	71
WWYW	WWGR (Q)	360	350	72
WWYW	YYWW	237	347	80
WWYW	_WW_	442	375	110
WWYW	_WWR	532	342	188
WWYW	____(4)	530	285	245
WWYW	WYBB	514	293	249
WWYW	WWRG	550	250	260
WWYW	WW_W	605	355	272
WWYW	____(5)	684	310	362
WWYW	_WW_	505	388	377
WWYW	WGRY	368	358	388
WWYW	WWGR (Q)	320	370	470
WWYW	YWRG	165	344	490
WWYW	YYWW	165	344	493
WWYW	_WWR	236	316	515
WWYW	YYWW	158	294	609
WWYW	__YY	208	236	1265
WWYW	WGGR	197	245	1368
WWYW	YYWW	170	291	1401
WWYW	_WWR	235	285	1516
WWYW	YWRG	215	315	1532
WWYW	_WWR	250	305	1610
__YY	WWYW	574	362	3
__YY	_WWR	580	331	6
__YY	WW_W	598	310	8
__YY	WGGR	204	283	30
__YY	YYWW	205	293	32
__YY	YWRG	216	369	50
__YY	WGGR	144	277	55
__YY	YWWB	446	319	74
__YY	WYBB	473	273	87
__YY	WW__	670	386	103
__YY	BBRR	738	364	110
__YY	____(6)	847	270	143
__YY	WY__	515	232	180
__YY	_RYY	190	216	191
__YY	GG_W	463	209	330
__YY	YYWW	140	340	378
__YY	WGGR	220	226	425
__YY	WWRG	251	196	667
__YY	WWYW	224	204	953
__YY	_GB_	600	200	1006

__YY	RWY_	675	245	1047
__YY	_GB_	600	226	1215
__YY	GG_W	377	221	1272
__YY	WWRG	404	204	1285
__YY	_RYY	513	226	1577
_BWW	WWBG	554	206	54
_BWW	____(4)	520	200	140
_BWW	white legs	520	206	152
_BWW	_RYY	526	200	225
_BWW	GG_W	517	197	339
_BWW	WWRG	543	202	470
_BWW	__YY	522	176	690
_BWW	WWBG	512	161	864
_BWW	WY__	510	160	1320
_BWW	_RYY	503	136	1482
_BWW	WY__	485	206	1576
_BWW	__YY	525	215	1583
GG_W	__YY	570	250	94
GG_W	_RRR	585	250	104
GG_W	white legs	560	260	116
GG_W	WWRG	565	283	119
GG_W	_RRR	571	291	139
GG_W	____(4)	532	267	154
GG_W	_BWW	516	240	191
GG_W	WYBB	500	250	200
GG_W	__YY	486	204	333
GG_W	WWRG	491	208	573
GG_W	_GB_	561	196	599
GG_W	WWRG	384	283	648
GG_W	_RRR	537	263	665
GG_W	____(6)	670	210	680
GG_W	WY__	670	210	683
GG_W	_GB_	610	204	687
GG_W	WY__	600	218	861
GG_W	WWRG	490	215	1200
GG_W	____(3)	490	215	1202
GG_W	WY__	490	215	1203
GG_W	WGGR	420	260	1242
GG_W	WWRG	425	237	1250
GG_W	____(4)	400	235	1256
GG_W	WGGR	300	253	1353
GG_W	_WWR	256	237	1358
GG_W	WY__	407	231	1721
WW_W	__YY	594	304	98
WW_W	_WWR	596	346	131
WW_W	_G__	617	373	155
WW_W	WWYW	560	365	292
WW_W	____(5)	600	335	433
WW_W	_RRR	583	309	755

WW_W	WW__	600	390	788
_RRR	GG_W	609	247	103
_RRR	WWRG	597	244	125
_RRR	WWYW	572	265	260
_RRR	WWRG	578	257	411
_RRR	_BWW	555	258	660
_RRR	WY__	558	258	930
____(5)	WW_W	630	305	3
____(5)	_RRR	660	295	23
____(5)	__YY	662	297	163
____(5)	WWRG	662	292	234
____(5)	WWYW	650	285	363
____(5)	RWY_	652	287	855
____(5)	__YY	653	283	1063
____(5)	RWY_	640	271	1753
____(5)	WWRG	641	272	1755
RWY_	WWRG	670	240	237
RWY_	WW__	705	214	349
RWY_	____(6)	660	222	484
RWY_	WW__	693	226	503
RWY_	GG_W	716	254	792
RWY_	____(6)	676	238	884
RWY_	__YY	710	247	1072
RWY_	____(6)	705	229	1098
RWY_	____(5)	671	280	1530
_G__	RWY_	727	259	9
_G__	WY__	816	205	19
_G__	WWYG	841	330	53
_G__	BBRR	797	351	65
_G__	WWYG	825	399	76
_G__	__YY	738	389	107
_G__	_WWR	630	392	124
_G__	WW_W	619	389	172
_G__	_WW_	475	400	317
_G__	WW_W	573	316	333
_G__	____(4)	527	226	346
_G__	WWRG	507	257	444
WW__	WWYG	842	380	64
WW__	_G__	842	378	72
WW__	BBRR	742	361	103
WW__	BBRR	727	333	620
WW__	WW_W	600	378	701
WW__	WW_W	680	390	998
BBRR	WW__	750	345	103
BBRR	__YY	755	343	110
BBRR	WWYG	836	334	309
BBRR	WWYG	838	350	494
BBRR	_GB_	788	335	500
BBRR	WW__	723	344	690

BBRR	RWY_	758	306	716
WWYG	WW__	860	368	42
WWYG	_G__	844	366	96
WY__	____(6)	831	197	3
WY__	_G__	832	200	22
WY__	RWY_	631	206	47
WY__	_BWW	614	209	69
WY__	_RRR	625	228	76
WY__	_RYY	489	211	95
WY__	_GB_	504	218	160
WY__	_BWW	504	227	169
WY__	_GB_	562	223	595
WY__	WWRG	559	222	605
WY__	_GB_	565	225	625
WY__	GG_W	626	206	688
WY__	GG_W	550	216	874
WY__	_BWW	526	207	915
WY__	_GB_	530	218	1135
WY__	GG_W	509	232	1199
WY__	__YY	410	215	1270
WY__	_BWW	488	201	1283
WY__	WWRG	480	205	1363
WY__	____(4)	510	230	1373
WY__	_RYY	478	208	1495
WY__	_BWW	475	215	1515
WY__	__YY	478	214	1553
WY__	_RYY	455	267	1591
____(6)	__YY	860	240	141
____(6)	WWRG	824	215	171
____(6)	_GB_	820	210	175
____(6)	WWRG	812	203	196
____(6)	WW_W	622	355	229
____(6)	WW__	750	255	290
____(6)	WWRG	629	230	298
____(6)	WY__	591	220	301
____(6)	_BWW	560	230	317
____(6)	GG_W	521	621	321
____(6)	____(4)	526	250	343
____(6)	GG_W	522	267	365
____(6)	WWYW	525	367	379
____(6)	WWYG	833	353	385
____(6)	RWY_	667	225	391
____(6)	__YY	678	208	1068
WWBG	_RYY	505	159	47
WWBG	_BWW	535	200	53
WWBG	WY__	577	208	81
WWBG	white legs	555	201	107
WWBG	_BWW	493	161	861
WWBG	__YY	431	206	873

WWBG	WWYW	213	195	905
WWBG	_RYY	518	110	1477
white legs	WWBG	526	184	98
white legs	_BWW	566	225	107
white legs	WWRG	526	238	118
white legs	_RYY	507	184	133
white legs	_G__	507	184	133
white legs	WWRG	578	195	148
white legs	_GB_	543	204	1283
white legs	WWRG	506	220	1286
_GB_	_RYY	500	162	97
_GB_	white legs	515	154	101
_GB_	_BWW	530	170	147
_GB_	WWRG	730	246	165
_GB_	____(6)	829	215	172
_GB_	BBRR	864	269	311
_GB_	WW__	777	244	319
_GB_	RWY_	734	284	436
_GB_	WY__	592	206	594
_GB_	GG_W	586	202	674
_GB_	_BWW	565	202	689
_GB_	WY__	554	203	1020
_GB_	WY__	548	208	1135
_GB_	_BWW	546	201	1148
_GB_	____(6)	722	206	1378

Antlist	InitialPosX	InitialPosY	ColonyArea	AntLength	MinX	MinY	MaxX	MaxY
WGGR	188	247	170688	62.5	97	0	883	381
YYWW	240	310						
YWRG	224	342						
WWGR (Q)	334	315						
____(1)	335	315						
WGRY	341	375						
____(2)	359	395						
____(3)	420	219						
YWWB	470	263						
WYBB	480	294						
_WW_	483	393						
_RYY	500	168						
WWRG	547	275						
____(4)	510	234						
_WWR	555	317						
WWYW	561	371						
__YY	575	374						
_BWW	551	200						
GG_W	580	242						
WW_W	580	283						
_RRR	615	243						
____(5)	624	337						
RWY_	675	235						
_G__	733	250						
WW__	758	386						
BBRR	798	363						
WWYG	842	377						
WY__	797	198						
____(6)	858	212						
WWBG	0	0						
white legs	0	0						
_GB_	0	0						
